# ﻿An updated checklist of fishes of Dongsha Island, Taiwan, northern South China Sea

**DOI:** 10.3897/zookeys.1220.131100

**Published:** 2024-12-09

**Authors:** Shing-Lai Ng, Hsin-Wei Liu, Dominique P. Mediodia, Yen-Ting Lin, Chieh-Hsuan Lee, Ching-Feng Lin, Shih-Pin Huang, Siao-Man Wu, Chih-Ren Tung, Hsuan-Ching Ho, Chien-Hsiang Lin

**Affiliations:** 1 Biodiversity Research Center, Academia Sinica, Taipei, Taiwan; 2 Department of Environmental Biology and Fisheries Science, National Taiwan Ocean University, Keelung, Taiwan; 3 Department of Life Science, National Taiwan Normal University, Taipei, Taiwan; 4 Biodiversity Program, Taiwan International Graduate Program, Academia Sinica and National Taiwan Normal University, Taipei, Taiwan; 5 Institute of Marine Fisheries and Oceanology, College of Fisheries and Ocean Sciences, University of the Philippines Visayas, Miagao, Philippines; 6 Department of Theatrical Design and Technology, Taipei National University of the Arts, Taipei, Taiwan; 7 Biodiversity Research Museum, Biodiversity Research Center, Academia Sinica, Taipei, Taiwan; 8 Department and Graduate Institution of Aquaculture, National Kaohsiung University of Science and Technology, Kaohsiung, Taiwan

**Keywords:** Biodiversity, ichthyofauna, ichthyology, Pratas Island, taxonomy

## Abstract

Dongsha Island, situated in the northern part of the South China Sea, is surrounded by coral reefs and deep-sea habitats. The coastal areas of the atoll, a marine protected area, serve as important nursery habitats for many reef fish species. At the same time, the offshore deep-sea continental slopes are historically important fishing grounds. Although previous inventories primarily focused on coral reef fishes within the atoll listing 652 species from 73 families, comprehensive surveys of fishes from deeper waters have been incomplete. In this study, the species composition of the fish fauna around Dongsha was updated by analyzing large volumes of frozen bycatch from commercial deep-sea trawlers operating in the area for the past four years and reviewing the existing literature. The species list increased to 1087 species belonging to 167 families, including several documented as new records and potentially undescribed species. This updated checklist also includes images of each species and most of their sagittal otoliths. This will assist further taxonomic work and significantly enhance understanding of marine biodiversity in the South China Sea.

## ﻿Introduction

The South China Sea is an arm of the western Pacific Ocean that borders the Southeast Asian mainland. Renowned for its significant marine biodiversity (Ng and Tan 2000; Liu 2013), the South China Sea also plays a crucial role in regional economic development and serves as a major fishing ground in bordering countries (Teh et al. 2019; Pauly and Liang 2020; Prince et al. 2023). Dongsha Island (also known as Dongsha Atoll or Pratas Island) is situated on the northern margin of the South China Sea, at the midpoint between Taiwan, Hong Kong, and Philippines. Dongsha is known for its coral reef ecosystems which support a diverse array of reef and seagrass-associated fishes (Chen et al. 1995; Lee et al. 2015). The island is governed by the Dongsha Atoll National Park of Taiwan and represents a critical marine biodiversity hotspot crucial for conservation and scientific studies (Dai 2004; Tkachenko and Soong 2017; Nieder et al. 2019).

Besides its coral reef ecosystems and associated fishes inhabiting Dongsha Atoll, the surrounding waters are also areas for commercial deep-sea fishing. Notably, the commercial trawl fishery, active in the deeper slope areas since the early 2000s, yields high volumes of deep-sea organisms. These catches, including a wide range of fish and invertebrate species (Komai et al. 2022, 2023), are highly diverse and are used primarily for aquaculture feeds. Despite their high biodiversity, research on these commercial species has only recently begun to receive substantial attention and there has been a rapid increase in the reporting of new fish species and records from these commercial trawl fishery sites in recent years (Ho and Lin 2022; Ho et al. 2023; Mediodia et al. 2024). However, new discoveries have overwhelmed current species lists, and there is a need to update these lists to accurately reflect the true baseline of fish diversity around Dongsha Island.

**Figure 1.**
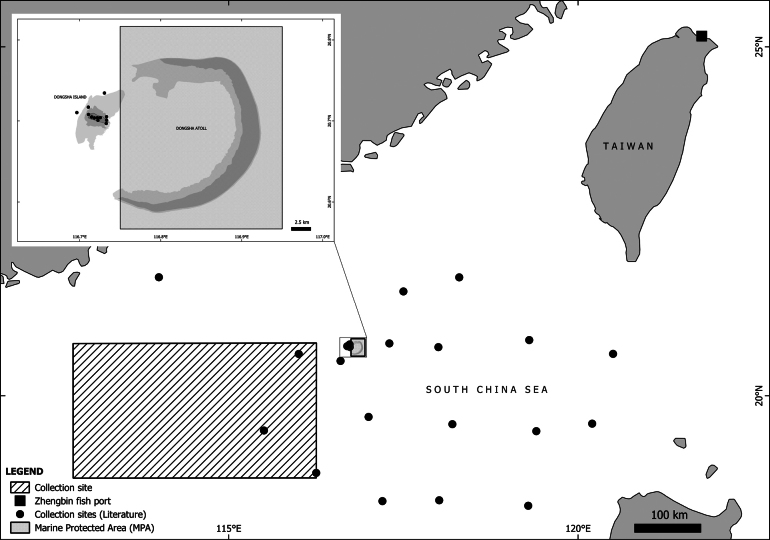
Map of Dongsha Atoll and the surrounding waters of the northern South China Sea showing the collection sites.

The purpose of this paper is to review and compile existing data and literature to provide a comprehensive overview of the fish fauna in and around Dongsha Island. While the list of coral and seagrass-associated fishes is derived from previous surveys outside of the marine protected area (Fig. 1), the list of deep-sea fish collected from the commercial trawl fishery is primarily based on our systematic sampling conducted over the past four years. In this updated checklist, we include records from both data sources but images of fresh specimens and otoliths are restricted to the deep-sea fishes. By updating this checklist, we aim to significantly enhance understanding of the region’s marine biodiversity, and make a crucial contribution to support the sustainable management of its marine resources.

## ﻿Materials and methods

Our study used two distinct datasets: occurrence records from the literature on coral and seagrass-associated shallow-water fishes and newly collected occurrence data from deep-water fishes sampled via commercial bottom trawlers. Both datasets represent different ecological settings with distinct sampling techniques. For the former, we reviewed existing literature (Chen et al. 1995; Shao et al. 2008; Shao et al. 2011; Chang et al. 2012; Ebert et al. 2013; Xu et al. 2019) and validated nomenclatures (Fricke et al. 2024). From February to early July each year from 2021 to 2024, we obtained frozen samples from fishermen at Zhengbin fish port in Keelung, Taiwan that were collected from the commercial bottom trawlers operating in the waters off Dongsha Island at depths of approximately 300–600 meters (Fig. 1; coordinates 18°49'N to 20°45'N and 112°46'E to 116°15'E). These samples consisted mainly of fishes and other invertebrates and weighed approximately 30 kg each. The total samples (*n* = 198) processed collectively weighed more than 6,000 kg. We collected samples bi-monthly, carefully selecting specimens to ensure they represented the broadest possible diversity within the catch. These samples were then transferred to our lab and defrosted sequentially.

Each fish was measured for standard length (**SL**), total length (**TL**), and weight (**WT**), with preanal length (**PAL**) recorded when necessary. Specimens were photographed before muscle tissue was subsampled from the right caudal region. These tissue samples were preserved in 95% ethyl alcohol and deposited at the National Academy of Marine Research in Kaohsiung for future reference.

For images of teleostean otoliths, the left sagittal otoliths were primarily used; right otoliths were reversed for consistency and noted with an (**R**) in each caption (Lin and Chang 2012). These otoliths were coated with a thin layer of gold using an ion sputter machine (MCM-100P, Sec, South Korea) to enhance the visibility of the sulcus. In addition, images of small-sized otoliths were captured using a scanning electron microscope (JSM-7100FLV, Jeol, Japan). Otoliths were archived at the Marine Paleontology Lab, Biodiversity Research Center, Academia Sinica, registered under the code CHLOL. We follow Nelson et al. (2016) for general classification and incorporate recent modifications from newly published research (Smith et al. 2022; Near and Thacker 2024). Scientific names and taxonomic attributions adhere to the latest edition of the Catalog of Fishes (Fricke et al. 2024). Our remarks provide information on species distribution and abundance estimates around Dongsha Island.

## ﻿Results

The occurrence dataset listed below was uploaded to the Global Biodiversity Information Facility (GBIF) and was published (Lin and Wu 2024). Representative fish specimens and their otoliths are presented in Figs 2–73.

**Figure 2.**
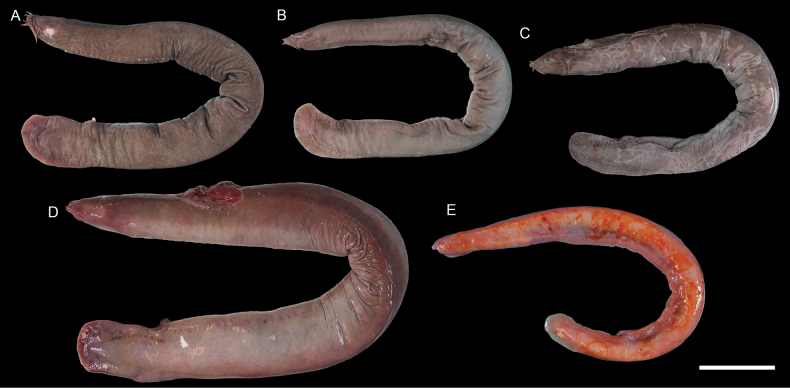
Images of fresh specimens collected around Dongsha Island **A***Eptatretusokinoseanus*, 335.37 mm TL**B***Eptatretussheni*, 344.32 mm TL**C***Eptatretusfernholmi*, 348.43 mm TL**D***Eptatretustaiwanae*, 416.49 mm TL**E***Rubicundusrubicundus*, 316.99 mm TL. Scale bar: 5 cm.

### ﻿Species recently described, redescribed, or first recorded around Dongsha Island (2021–2024)

***Apristurusnakayai* Iglésias, 2012**

Fig. 4E

This species was originally described from New Caledonia, with one record from Papua New Guinea, and was recently recorded around Dongsha Island (Ng et al. 2023b). It is common in the area.

***Iagogarricki* Fourmanoir & Rivaton, 1979**

Fig. 4J

This species is restricted to the tropical western Pacific, and is recently redescribed based on five specimens around Dongsha Island (Ng et al. 2022a).

***Etmopteruslii* Ng, Liu & Joung, 2024**

Fig. 5D

This species was recently described from the northern South China Sea, where we collected our samples. Juveniles are more common around Dongsha Island, while adults are rare, suggesting that the latter may inhabit deeper waters.

***Okamejeipicta* Ng, Ho, Joung & Liu, 2023**

Fig. 3H

This species was described around Dongsha Island, based on two specimens (Ng et al. 2023a). No additional specimens were collected after the description.

**Figure 3.**
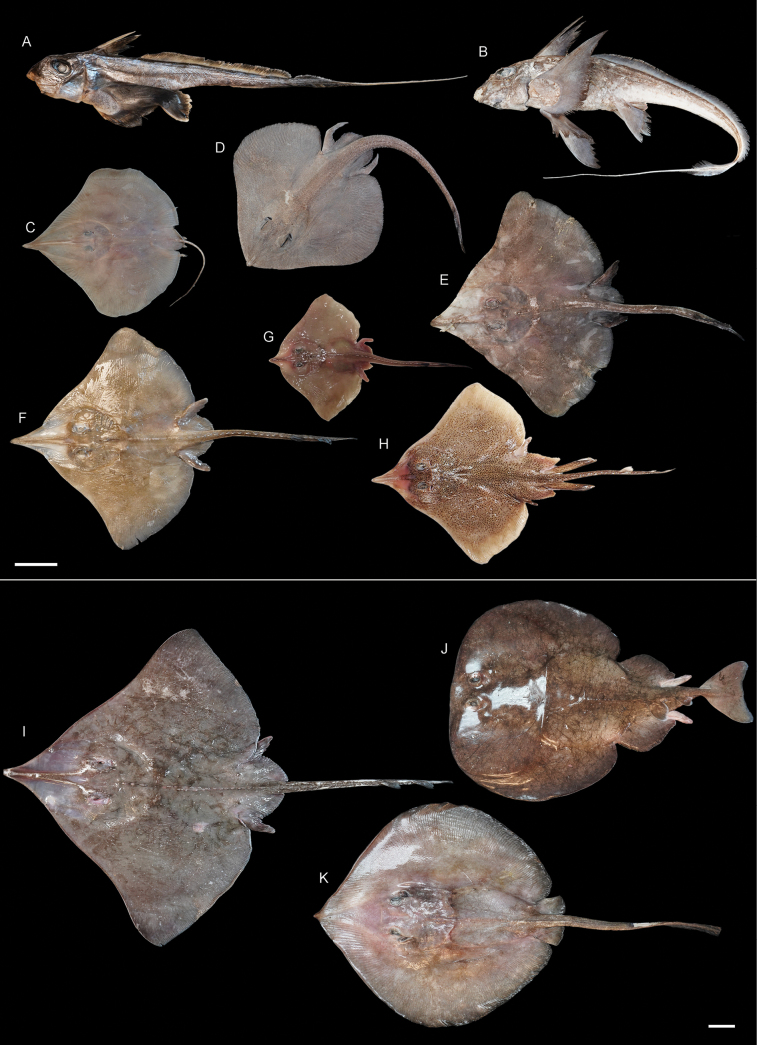
Images of fresh specimens collected around Dongsha Island **A***Chimaeraphantasma*, 511 mm TL**B***Hydrolagusmitsukurii*, 633 mm TL**C***Sinobatisborneensis*, 338 mm TL**D***Notorajatobitukai*, 448 mm TL**E***Dipturusgigas*, 404 mm TL**F***Dipturustengu*, 400 mm TL**G***Okamejei* sp., 266 mm TL**H***Okamejeipicta*, 429 mm TL**I***Dipturuswuhanlingi*, 753 mm TL**J***Tetronarcetokionis*, 576 mm TL**K***Plesiobatisdaviesi*, 801 mm TL. Scale bars: 5 cm.

**Figure 4.**
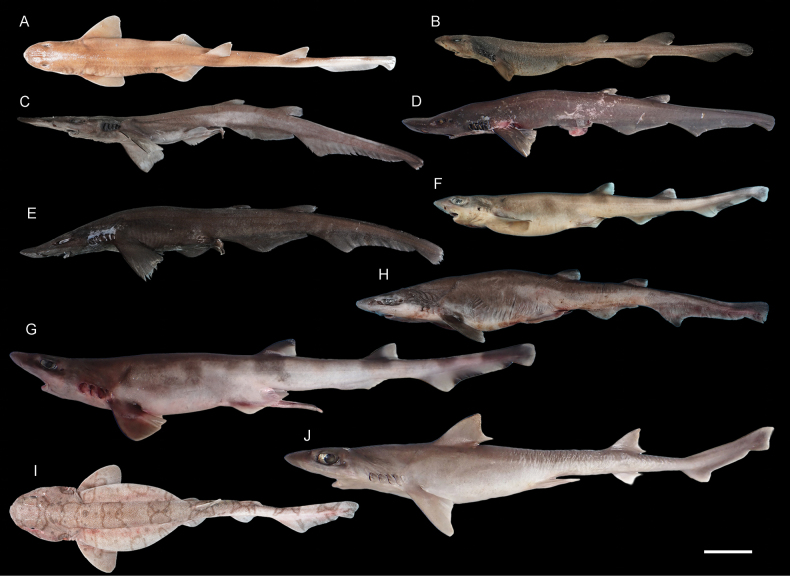
Images of fresh specimens collected around Dongsha Island **A***Cirrhoscylliumformosanum*, 380 mm TL**B***Dichichthysmelanobranchus*, 320 mm TL**C***Apristurusherklotsi*, 413 mm TL**D***Apristurusmacrostomus*, 361 mm TL**E***Apristurusnakayai*, 421 mm TL**F***Galeuseastmani*, 342 mm TL**G***Galeusnipponensis*, 566 mm TL**H***Galeussauteri*, 408 mm TL**I***Cephaloscylliumfasciatum*, 356 mm TL**J***Iagogarricki*, 515 mm TL. Scale bar: 5 cm.

**Figure 5.**
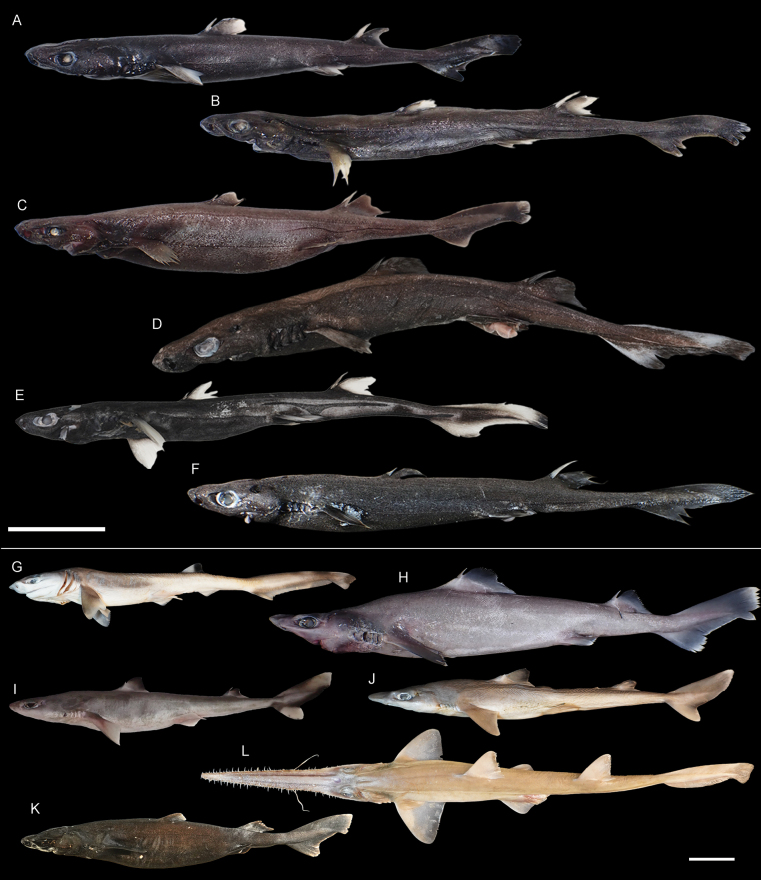
Images of fresh specimens collected around Dongsha Island **A***Etmopterusbigelowi*, 261 mm TL**B***Etmopterusbrachyurus*, 268 mm TL**C***Etmopterusdecacuspidatus*, 270 mm TL**D***Etmopteruslii*, 296 mm TL**E**Etmopteruscf.molleri, 264 mm TL**F***Etmopteruslucifer*, 287 mm TL**G***Heptranchiasperlo*, 383 mm TL**H***Centrophorustessellatus*, 613 mm TL**I***Squalusmontalbani*, 371 mm TL**J***Squalusjaponicus*, 410 mm TL**K***Zameussquamulosus*, 356 mm TL**L***Pristiophorusjaponicus*, 622 mm TL. Scale bars: 5 cm.

***Ophichthuskusanagi* Hibino, McCosker & Tashiro, 2019**

Fig. 9G

This species was described from Japan and reported around Dongsha Island (Ho et al. 2022). It is rare around Dongsha Island.

***Ophichthuspratasensis* Ho, Ng & Lin, 2022**

Figs 9E, 10A

This species was described around Dongsha Island (Ho et al. 2022) and is rare in the area.

***Congriscusmaldivensis* (Norman, 1939)**

Figs 11B, 12B

This species is widespread in the Indo-West Pacific and was recently recorded by Huang et al. (in press.) around Dongsha Island. This species is common in the area but not found around Taiwan.

***Lestrolepisphilippina* (Fowler, 1934)**

Figs 25G, 26F

This species was commonly misidentified as *Lestrolepisjaponica* over the past few decades (e.g., Ho et al. (2019b), till Ho and Kawai (2024) verified their identities.

***Kuronezumiamacronema* (Smith & Radcliffe, 1912)**

Figs 34B, 37B

This species was originally described from the Philippines and was recorded around Dongsha Island (Ng et al. 2022b). No other specimen was collected after this record.

***Amarsipuscarlsbergi* Haedrich, 1969**

Figs 55E, 56B

This species is widespread in the Indo-West Pacific and was reported as a new record around Dongsha Island by Ho et al. (2023). It is very rare in the area.

***Synagropsatrumoris* Mediodia & Lin, 2024**

Figs 57F, 58C

This recently described new species (Mediodia et al. 2024) is not common in our sampling, and it is often difficult to distinguish from its sympatric congener, *S.japonicus* (Döderlein, 1883).

***Lophiodeslugubris* (Alcock, 1894)**

Figs 67B, 68D

This species is widely distributed in the Indo-West Pacific and was recently redescribed by Ho and Lin (2022).

***Lophiodestriradiatus* (Lloyd, 1909)**

Figs 67E, 68F

This deepwater species was only found around Dongsha Island and was not recorded around Taiwan. Ho and Lin (2022) reported larger specimens from Dongsha Island, which are larger than those reported from other countries.

### ﻿New records for Dongsha Island

***Eptatretussheni* (Kuo, Huang & Mok, 1994)**

Fig. 2B

This species was described from Taiwan. Though common around Taiwan, it was not reported in other regions. Our records around Dongsha Island show a substantial range extension. This species is rare around Dongsha Island.

***Eptatretustaiwanae* (Shen & Tao, 1975)**

Fig. 2D

This species was described from Taiwan and is common in the area. It was recognized as endemic. Our first record around Dongsha Island suggests a broader distribution range. This species is rare around Dongsha Island.

***Rubicundusrubicundus* (Kuo, Lee & Mok, 2010)**

Fig. 2E

This attractive cuskeel species was described from Taiwan and is considered endemic. The sole specimen we collected represents the first record around Dongsha Island. This species is very rare around Dongsha Island.

***Centrophorustessellatus* Garman, 1906**

Fig. 5H

Some species of the genus *Centrophorus* are very difficult to identify, including a long-snout species group. Within this group, two species occur in the northwestern Pacific, *C.tessellatus* and *C.isodon* Chu, Meng & Liu, 1981. We tentatively identified the long-snout specimens as *C.tessellatus*, which is not uncommon in southwestern Taiwan and is recorded in Japan. This species is uncommon around Dongsha Island, and most specimens are juveniles.

***Etmopterusbigelowi* Shirai & Tachikawa, 1993**

Fig. 5A

This species is one of the circumglobal lanternshark species, yet records from the northwestern Pacific were limited to off Japan. Five specimens were collected in our study, confirming their occurrence around Dongsha Island, South China Sea.

***Squalusmontalbani* Whitley, 1931**

Fig. 5I

One of the widespread spurdog species, having records from northeastern Taiwan to northern Australia. Yet, no records are known from the South China Sea. This species is common around Dongsha Island. Large individuals are sold separately.

***Atractodenchelysbrevitrunca* Vo & Ho, 2020**

Figs 6I, 7I

This species was recently described in central Vietnam by Vo and Ho (2020). The novel record based on several specimens around Dongsha Island we examined suggests that this species has a broad distribution range in the South China Sea.

**Figure 6.**
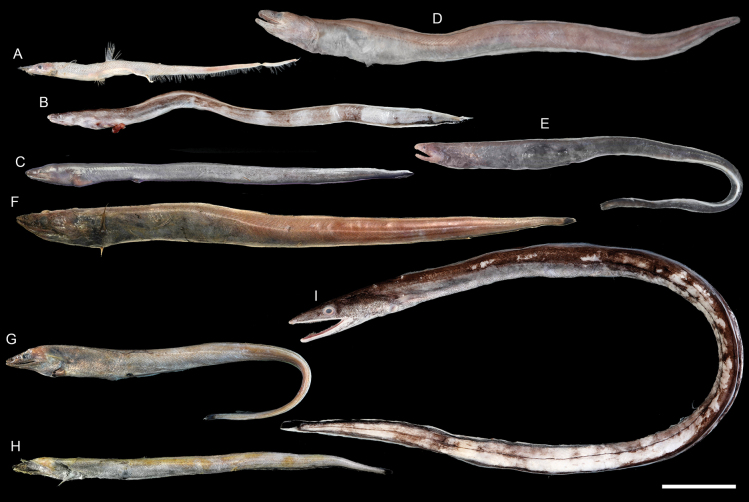
Images of fresh specimens collected around Dongsha Island **A***Aldrovandiaaffinis*, 188.65 mm TL**B***Dysommapolycatodon*, 241.19 mm TL**C***Dysommaanguillare*, 225.71 mm TL**D***Dysomminaorientalis*, 290.99 mm TL**E***Dysommadolichosomatum*, 290.86 mm TL**F***Synaphobranchuskaupii*, 330.85 mm TL**G***Synaphobranchusoligolepis*, 252.15 mm TL**H***Synaphobranchusaffinis*, 215.86 mm TL**I***Atractodenchelysbrevitrunca*, 605.05 mm TL. Scale bar: 5 cm.

***Dysommapolycatodon* Karrer, 1983**

Figs 6B, 7G

This species is widely distributed on continental shelf in the Indo-West Pacific, and is common around Taiwan. Only one specimen was recorded around Dongsha Island, suggesting the shallower habitat of this species.

**Figure 7.**
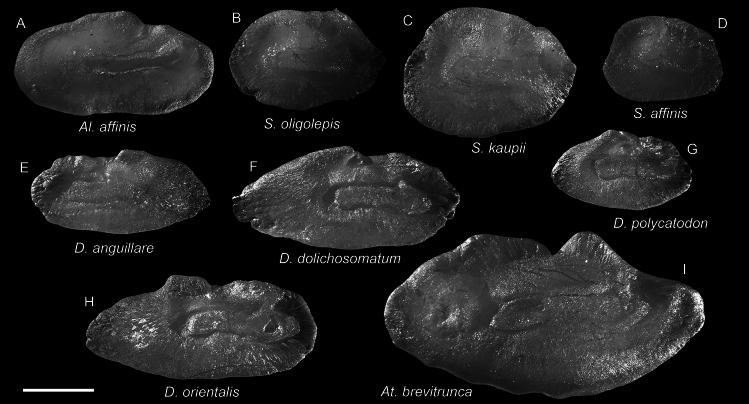
Otolith images of fresh specimens collected around Dongsha Island **A***Aldrovandiaaffinis*, CHLOL 20601, 204.25 mm TL**B***Synaphobranchusoligolepis*, CHLOL 23597, 275.98 mm TL**C***Synaphobranchuskaupii*, CHLOL 23598, 330.85 mm TL (R) **D***Synaphobranchusaffinis*, CHLOL 26162, 215.86 mm TL**E***Dysommaanguillare*, CHLOL 25422, 225.71 mm TL (R) **F***Dysommadolichosomatum*, CHLOL 20664, 322.95 mm TL**G***Dysommapolycatodon*, CHLOL 9815, 241.19 mm TL**H***Dysomminaorientalis*, CHLOL 20492, 294.48 mm TL**I***Atractodenchelysbrevitrunca*, CHLOL 25213, 494.47 mm TL. Scale bar: 1 mm.

***Synaphobranchusoligolepis* Ho, Hong & Chen, 2018**

Figs 6G, 7B

This species was recently described around Taiwan. It is common near the type locality but rare around Dongsha Island.

***Colocongermaculatus* Ho & Tang, 2021**

Figs 8A, 10H

This species was recently described around Taiwan based on a single specimen. Our specimen collected around Dongsha Island is the second known specimen.

**Figure 8.**
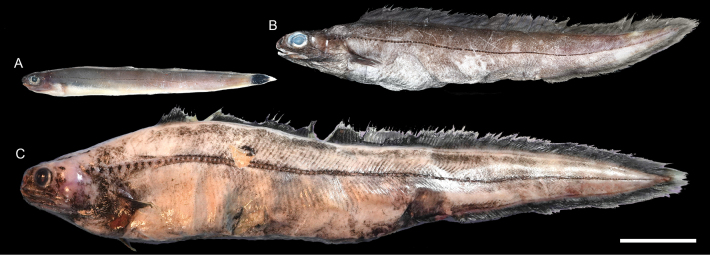
Images of fresh specimens collected around Dongsha Island **A***Colocongermaculatus*, 171.06 mm TL**B***Colocongerscholesi*, 274.87 mm TL**C***Colocongerraniceps*, 461.33 mm TL. Scale bar: 5 cm.

***Colocongerraniceps* Alcock, 1889**

Figs 8C, 10I

This species is widespread in the Indo-West Pacific, but is not well presented in museums. It is rare around Dongsha Island.

***Ophichthusmegalops* Asano, 1987**

Figs 9H, 10B

This species was originally described from Japan. It is occasionally found in bycatches of bottom trawl around Taiwan, but it is rare around Dongsha Island.

**Figure 9.**
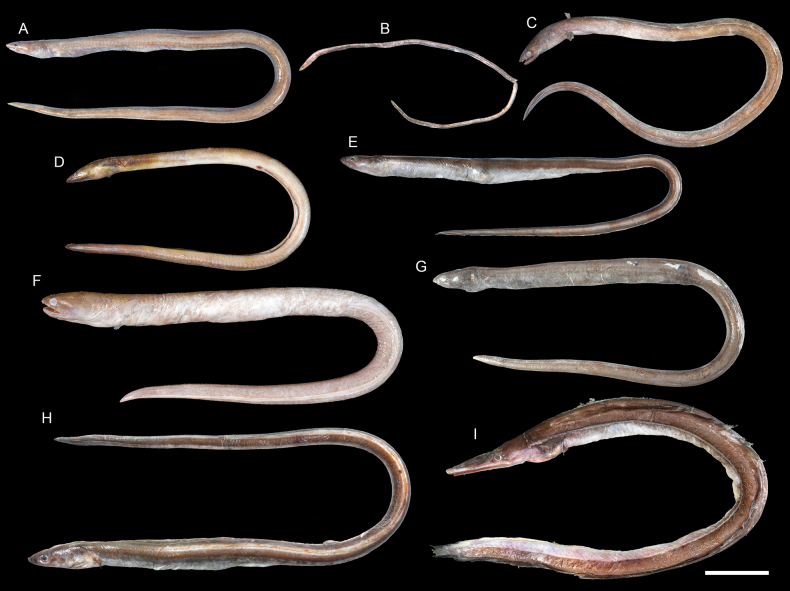
Images of fresh specimens collected around Dongsha Island **A***Pisodonophisboro*, 476.99 mm TL**B***Neenchelys* sp., 311.24 mm TL**C***Ophichthusobtusus*, 467.93 mm TL**D***Ophichthus* sp., 448.31 mm TL**E***Ophichthuspratasensis*, 860.23 mm TL**F***Ophichthusurolophus*, 549.79 mm TL**G***Ophichthuskusanagi*, 504.80 mm TL**H***Ophichthusmegalops*, 592.09 mm TL**I***Nettastomasolitarium*, 557.13 mm TL. Scale bar: 5 cm.

**Figure 10.**
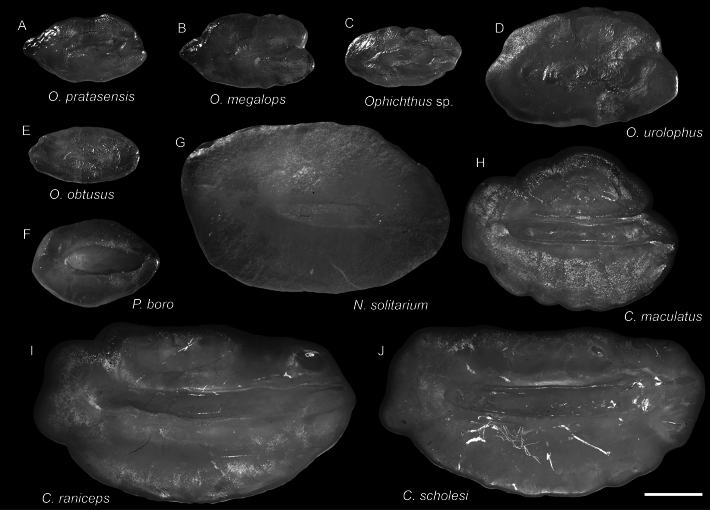
Otolith images of fresh specimens collected around Dongsha Island **A***Ophichthuspratasensis*, CHLOL 20629, 495.21 mm TL**B***Ophichthusmegalops*, CHLOL 22042, 592.09 mm TL**C***Ophichthus* sp., CHLOL 17929, 442.76 mm TL**D***Ophichthusurolophus*, CHLOL 9821, 577.00 mm TL**E***Ophichthusobtusus*, CHLOL 19481, 453.22 mm TL**F***Pisodonophisboro*, CHLOL 21208, 476.99 mm TL**G***Nettastomasolitarium*, CHLOL 29404, 648.86 mm TL**H***Colocongermaculatus*, CHLOL 20620, 171.06 mm TL**I***Colocongerraniceps*, CHLOL 27101, 207.50 mm TL**J***Colocongerscholesi*, CHLOL 27100, 191.54 mm TL. Scale bar: 1 mm.

***Ophichthusobtusus* McCosker, Ide & Endo, 2012**

Figs 9C, 10E

This species is restricted to the northwestern Pacific and has records from Japan, Taiwan, and Vietnam. It is common around Dongsha Island and Taiwan.

***Acromycternezumi* (Asano, 1958)**

Figs 11F, 14A

This species is restricted to the northwestern Pacific. It is common around Dongsha Island but rare around Taiwan.

**Figure 11.**
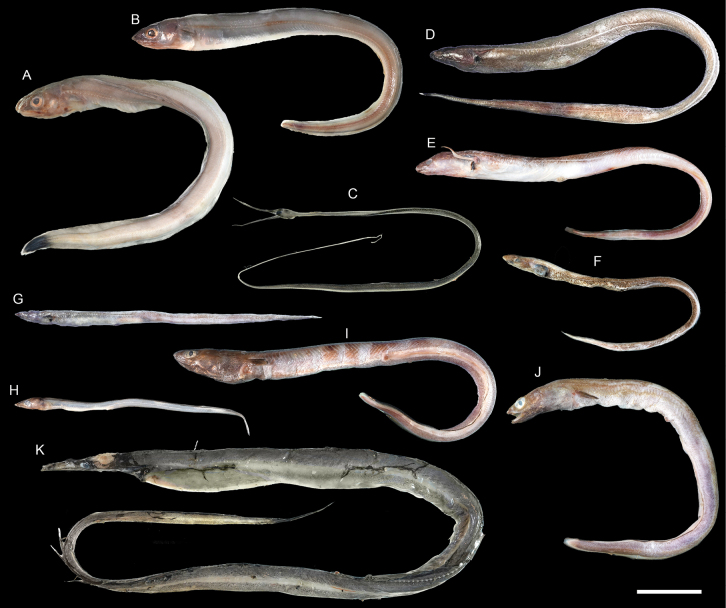
Images of fresh specimens collected around Dongsha Island **A***Congriscusmegastoma*, 348.09 mm TL**B***Congriscusmaldivensis*, 312.06 mm TL**C***Nemichthysscolopaceus*, 507.37 mm TL**D***Macrocephenchelysbrachialis*, 461.81 mm TL**E***Macrocephenchelysbrevirostris*, 36.052 mm TL**F***Acromycternezumi*, 272.56 mm TL**G***Blacheaxenobranchialis*, 227.95 mm TL**H***Gnathophisheterognathos*, 183.87 mm TL**I***Ariosomameeki*, 367.31 mm TL**J***Ariosomaemmae*, 329.76 mm TL**K***Gavialicepstaiwanensis*, 690.19 mm TL. Scale bar: 5 cm.

***Ariosomaemmae* Smith & Ho, 2018**

Figs 11J, 12I

This species was described from southwestern Taiwan. It is rare around Dongsha Island.

**Figure 12.**
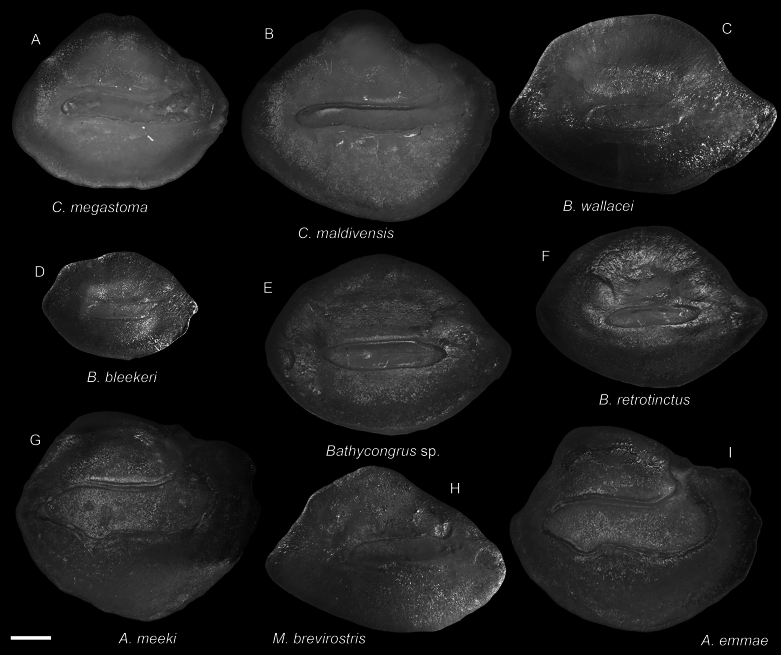
Otolith images of fresh specimens collected around Dongsha Island **A***Congriscusmegastoma*, CHLOL 27848, 307.35 mm TL**B***Congriscusmaldivensis*, CHLOL 27847, 217.73 mm TL**C***Bathycongruswallacei*, CHLOL 20479, 333.05 mm TL**D***Bathycongrusbleekeri*, CHLOL 19487, 179.51 mm TL**E***Bathycongrus* sp., CHLOL 20668, 315.03 mm TL**F***Bathycongrusretrotinctus*, CHLOL 22039, 300.22 mm TL**G***Ariosomameeki*, CHLOL 20626, 326.77 mm TL**H***Macrocephenchelysbrevirostris*, CHLOL 9816, 360.52 mm TL**I***Ariosomaemmae*, CHLOL 21649, 302.42 mm TL. Scale bar: 1 mm.

***Bathycongrusbimaculatus* Smith & Ho, 2018**

Fig. 13D

This species was described from southwestern Taiwan. The sole specimen around Dongsha Island, suggests a broader distribution in the South China Sea.

**Figure 13.**
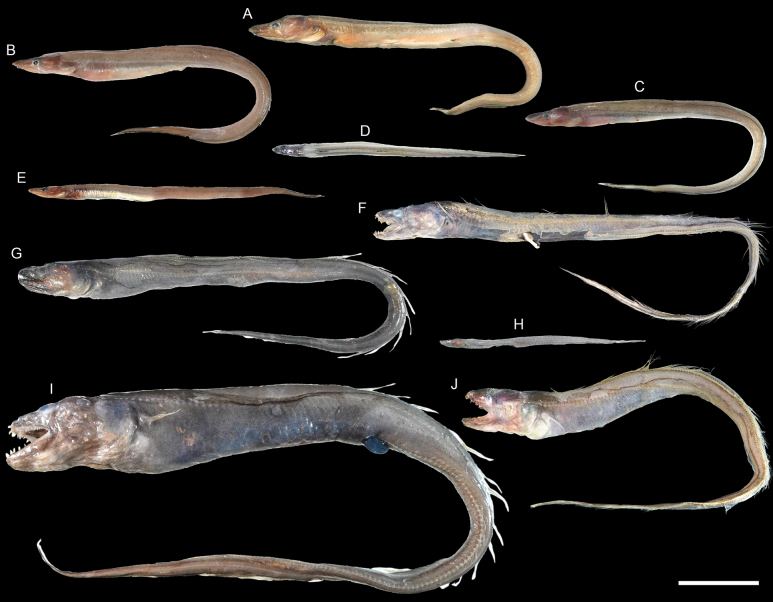
Images of fresh specimens collected around Dongsha Island **A***Bathycongrusretrotinctus*, 254.48 mm TL**B***Bathycongruswallacei*, 273.51 mm TL**C***Bathycongrus* sp., 275.39 mm TL**D***Bathycongrusbimaculatus*, 153.61 mm TL**E***Bathycongrusbleekeri*, 179.51 mm TL**F**Bathyurocongercf.vicinus, 378.46 mm TL**G***Bathyurocongerparvibranchialis*, 729.12 mm TL**H***Bathyurocongeralbus*, 127.79 mm TL**I***Bathyuroconger* sp., 494.24 mm TL**J***Bathyurocongerfowleri*, 375.93 mm TL. Scale bar: 5 cm.

***Bathycongrusbleekeri* Fowler, 1934**

Figs 12D, 13E

This species is restricted to the northwestern Pacific. It is rare around Dongsha Island.

***Bathyurocongerparvibranchialis* (Fowler, 1934)**

Figs 13G, 14I

This species was originally described from the Philippines. It was recently documented in Taiwan by Ho et al. (2015) and redescribed by Smith et al. (2018). It is rare around Dongsha Island.

**Figure 14.**
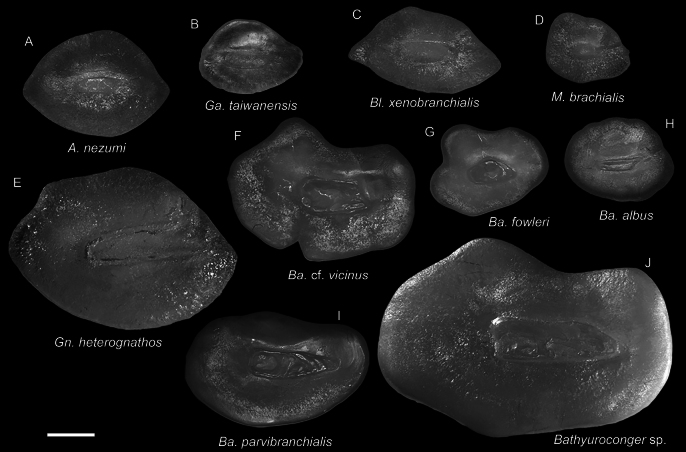
Otolith images of fresh specimens collected around Dongsha Island **A***Acromycternezumi*, CHLOL 20456, 208.92 mm TL**B***Gavialicepstaiwanensis*, CHLOL 21638, 620.48 mm TL**C***Blacheaxenobranchialis*, CHLOL 22753, 257.88 mm TL**D***Macrocephenchelysbrachialis*, CHLOL 25636, 461.81 mm TL**E***Gnathophisheterognathos*, CHLOL 9812, 183.87 mm TL**F**Bathyurocongercf.vicinus, CHLOL 27098, 378.46 mm TL**G***Bathyurocongerfowleri*, CHLOL 22668, 234.89 mm TL**H***Bathyurocongeralbus*, CHLOL 22038, 127.79 mm TL**I***Bathyurocongerparvibranchialis*, CHLOL 22077, 729.12 mm TL**J***Bathyuroconger* sp., CHLOL 16037, 494.24 mm TL. Scale bar: 1 mm.

***Blacheaxenobranchialis* Karrer & Smith, 1980**

Figs 11G, 14C

This species is widespread in the Indo-West Pacific and was previously reported from Taiwan by Ho and Shao (2010) based on a single specimen. We additionally collected two specimens around Dongsha Island, confirming its occurrence in the South China Sea.

***Congriscusmegastoma* (Günther, 1877)**

Figs 11A, 12A

This species is restricted to the northwestern Pacific. It is not uncommon around Dongsha Island.

***Macrocephenchelysbrachialis* Fowler, 1934**

Figs 11D, 14D

This species is widespread in the Indo-West Pacific. It is common around Taiwan, yet only one specimen was collected around Dongsha Island.

***Rouleinasquamilatera* (Alcock, 1898)**

Figs 15E, 16D

This species has scattered distribution in the Indo-West Pacific. It is rare around Dongsha Island.

**Figure 15.**
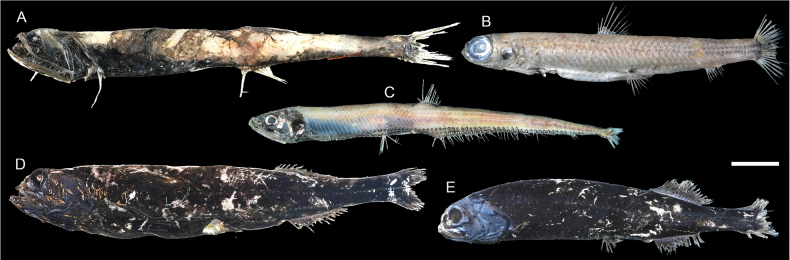
Images of fresh specimens collected around Dongsha Island **A***Sigmopselongatus*, 146.75 mm SL**B***Nanseniaardesiaca*, 187.71 mm SL**C***Diplophosvicinia*, 138.84 mm SL**D***Rouleinawatasei*, 221.15 mm SL**E***Rouleinasquamilatera*, 202.20 mm SL. Scale bar: 3 cm.

**Figure 16.**
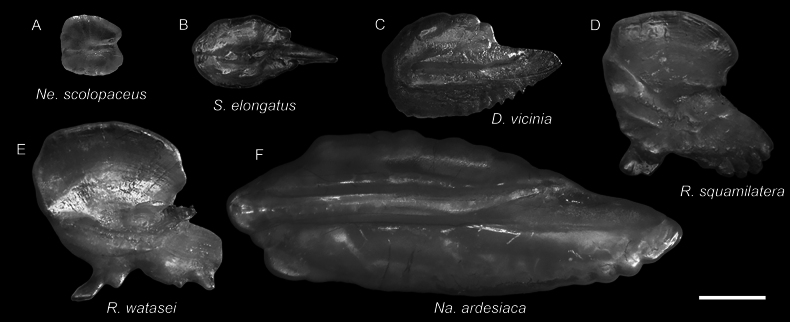
Otolith images of fresh specimens collected around Dongsha Island **A***Nemichthysscolopaceus*, CHLOL 23020, 641.46 mm TL**B***Sigmopselongatus*, CHLOL 15420, 146.75 mm SL**C***Diplophosvicinia*, CHLOL 19048, 138.84 mm SL**D***Rouleinasquamilatera*, CHLOL 15938 166.50 mm SL**E***Rouleinawatasei*, CHLOL 16466, 221.15 mm SL**F***Nanseniaardesiaca*, CHLOL 20443, 142.76 mm SL. Scale bar: 1 mm.

***Diplophosvicinia* Koeda & Ho, 2019**

Figs 15C, 16C

This species was recently described from southern Taiwan and Papua New Guinea. The specimens we collected around Dongsha Island, suggest that this species is also found in the South China Sea and is sympatric with the morphologically similar congener *D.taenia*.

***Polyipnusmatsubarai* Schultz, 1961**

Figs 17A, 18A

This species has been reported from Japan and Hawaii (Emperor Seamounts). The occurrence around Dongsha Island suggests its broad distribution in the northwestern Pacific to Hawaii.

**Figure 17.**
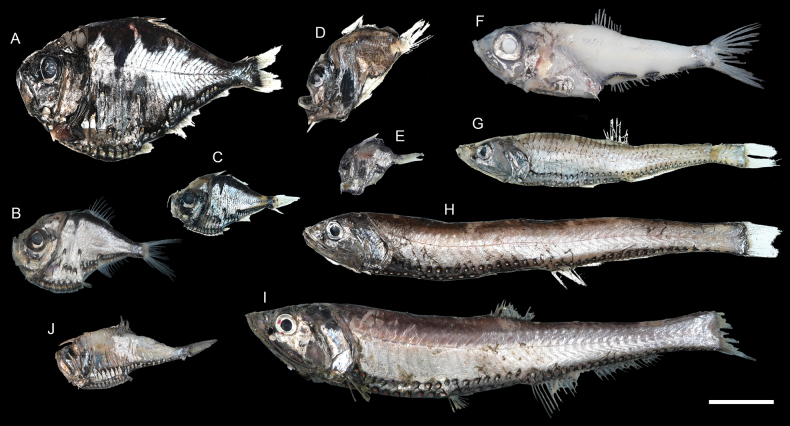
Images of fresh specimens collected around Dongsha Island **A***Polyipnusmatsubarai*, 79.97 mm SL**B***Polyipnusspinifer*, 54.97 mm SL**C***Polyipnusstereope*, 37.93 mm SL**D***Sternoptyxpseudobscura*, 24.90 mm SL**E***Sternoptyxdiaphana*, 24.19 mm SL**F***Argyripnus* sp., 87.00 mm SL**G***Polymetmesurugaensis*, 115.57 mm SL**H***Polymetmeelongata*, 124.14 mm SL**I***Polymetmecorythaeola*, 214.45 mm SL**J***Argyropelecusaffinis*, 46.60 mm SL. Scale bar: 3 cm.

**Figure 18.**
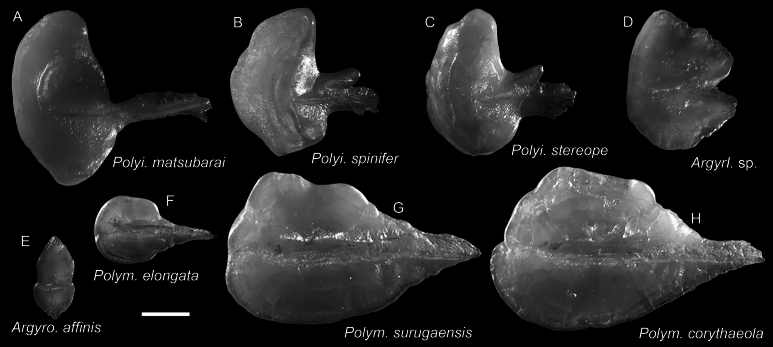
Otolith images of fresh specimens collected around Dongsha Island **A***Polyipnusmatsubarai*, CHLOL 16404, 79.97 mm SL**B***Polyipnusspinifer*, CHLOL 21531, 52.83 mm SL**C***Polyipnusstereope*, CHLOL 20639, 43.47 mm SL**D***Argyripnus* sp., CHLOL 3446, 86.97 mm SL**E***Argyropelecusaffinis*, CHLOL 15486, 60.46 mm SL**F***Polymetmeelongata*, CHLOL 20610, 154.19 mm SL**G***Polymetmesurugaensis*, CHLOL 20580, 187.44 mm SL**H***Polymetmecorythaeola*, CHLOL 29482, 214.45 mm SL. Scale bar: 1 mm.

***Polyipnusspinifer* Borodulina, 1979**

Figs 17B, 18B

This species is widespread in the western Pacific. It is not uncommon around Taiwan, especially in mid-water trawlers, but rare around Dongsha Island.

***Polymetmecorythaeola* (Alcock, 1898)**

Figs 17I, 18H

This species can be found in the western Pacific. It is very common around Dongsha Island but rare around Taiwan.

***Polymetmesurugaensis* (Matsubara, 1943)**

Figs 17G, 18G

This species is widespread in the western Pacific. It is common in mid-water trawlers around Taiwan but rare around Dongsha Island.

***Borostomiaselucens* (Brauer, 1906)**

Figs 21A, 22G

While having circumglobal distribution in tropical and temperate oceans, this species is rare around Dongsha Island.

***Borostomiaspacificus* (Imai, 1941)**

Figs 19I, 22H

This species is restricted to the northwestern Pacific and is rare around Dongsha Island.

**Figure 19.**
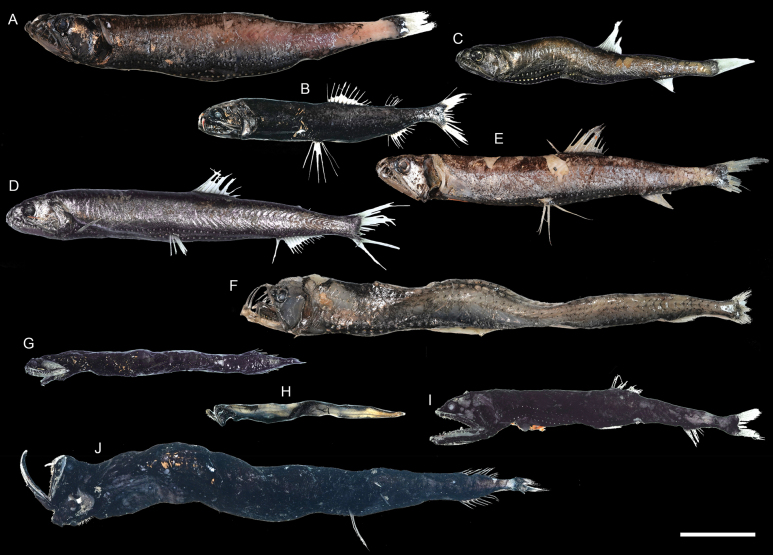
Images of fresh specimens collected around Dongsha Island **A***Astronestheslucifer*, 100.27 mm SL**B***Astronesthesindica*, 84.31 mm SL**C***Astronestheschrysophekadion*, 96.90 mm SL**D***Astronesthesindopacifica*, 129.70 mm SL**E***Astronesthestrifibulata*, 123.68 mm SL**F***Chauliodussloani*, 176.27 mm SL**G***Photostomiastantillux*, 105.26 mm SL**H***Stomiasnebulosus*, 76.28 mm SL**I***Borostomiaspacificus*, 113.63 mm SL**J***Photonectesalbipennis*, 191.94 mm SL. Scale bar: 3 cm.

***Photostomiastantillux* Kenaley, 2009**

Figs 19G, 20C

This species is widespread in the Pacific. Only one specimen was collected around Dongsha Island.

**Figure 20.**
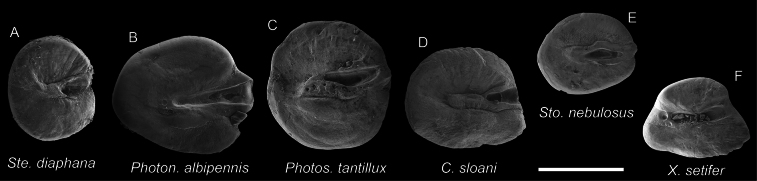
Otolith images of fresh specimens collected around Dongsha Island. Scanning electron microscope images of **A***Sternoptyxdiaphana*, CHLOL 21042, 24.19 mm SL**B***Photonectesalbipennis*, CHLOL 17271, 191.94 mm SL**C***Photostomiastantillux*, CHLOL 20618, 105.26 mm SL**D***Chauliodussloani*, CHLOL 20056, 159.87 mm SL**E***Stomiasnebulosus*, CHLOL 29697 92.64 mm SL**F***Xiphasiasetifer*, CHLOL 26196, 312.83 mm TL. Scale bar: 0.5 mm.

**Figure 21.**
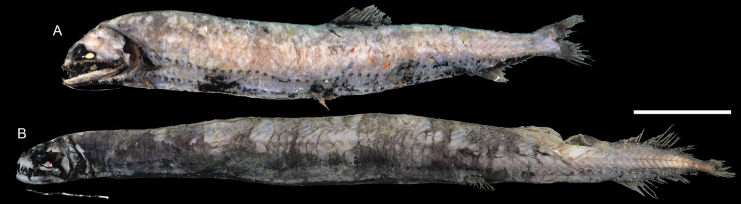
Images of fresh specimens collected around Dongsha Island **A***Borostomiaselucens*, 245.61 mm SL**B***Leptostomiasrobustus*, 340.30 mm SL. Scale bar: 5 cm.

**Figure 22.**
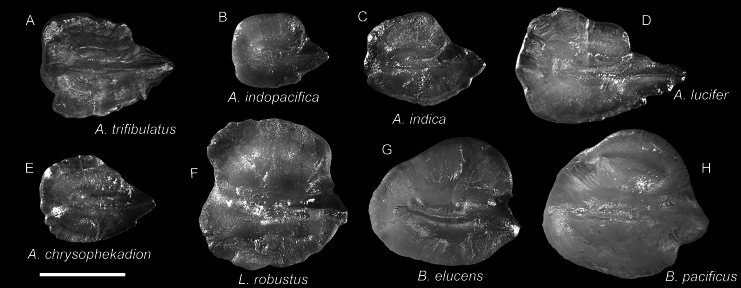
Otolith images of fresh specimens collected around Dongsha Island **A***Astronesthestrifibulata*, CHLOL 15414, 123.68 mm SL**B***Astronesthesindopacifica*, CHLOL 22771, 74.26 mm SL**C***Astronesthesindica*, CHLOL 21597, 84.31 mm SL**D***Astronestheslucifer*, CHLOL 26368, 118.29 mm SL**E***Astronestheschrysophekadion*, CHLOL 23215, 96.90 mm SL**F***Leptostomiasrobustus*, CHLOL 27092, 338.66 mm SL**G***Borostomiaselucens*, CHLOL 27093, 245.61 mm SL**H***Borostomiaspacificus*, CHLOL 28799, 223.81 mm SL. Scale bar: 1 mm.

***Chlorophthalmuspectoralis* Okamaura & Doi, 1984**

Figs 23B, 24B

This species is found in the western Pacific. It is common around Dongsha Island but rare around Taiwan.

**Figure 23.**
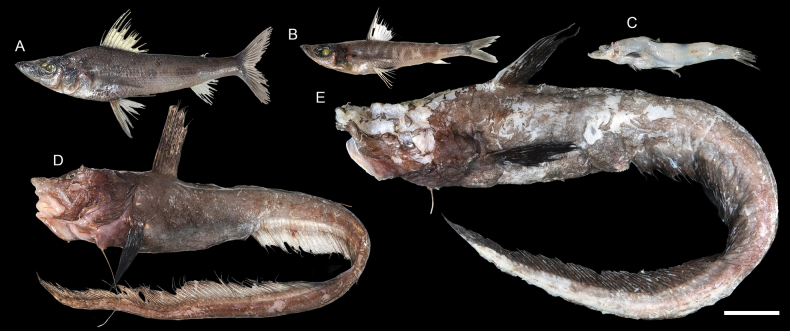
Images of fresh specimens collected around Dongsha Island **A***Chlorophthalmusacutifrons*, 204.23 mm SL**B***Chlorophthalmuspectoralis*, 145.18 mm SL**C***Rosenblattichthysalatus*, 121.03 mm SL**D***Ateleopusjaponicus*, 621.62 mm TL**E***Ijimaiadofleini*, 706.63 mm SL. Scale bar: 5 cm.

**Figure 24.**
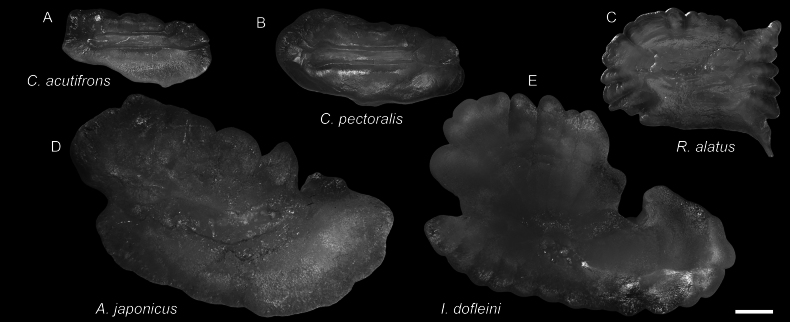
Otolith images of fresh specimens collected around Dongsha Island **A***Chlorophthalmusacutifrons*, CHLOL 20303, 104.77 mm SL**B***Chlorophthalmuspectoralis*, CHLOL 21197, 159.37 mm SL**C***Rosenblattichthysalatus*, CHLOL 17910, 121.03 mm SL**D***Ateleopusjaponicus*, CHLOL 21595, 233.22 mm SL**E***Ijimaiadofleini*, CHLOL 19918, 727.27 mm SL. Scale bar: 1 mm.

***Dolichosudisfuliginosa* Post, 1969**

Figs 25I, 26A

This species is widely distributed in the Atlantic and Pacific. Ho et al. (2019a) reported this species from southern Taiwan. It is rare around Dongsha Island.

**Figure 25.**
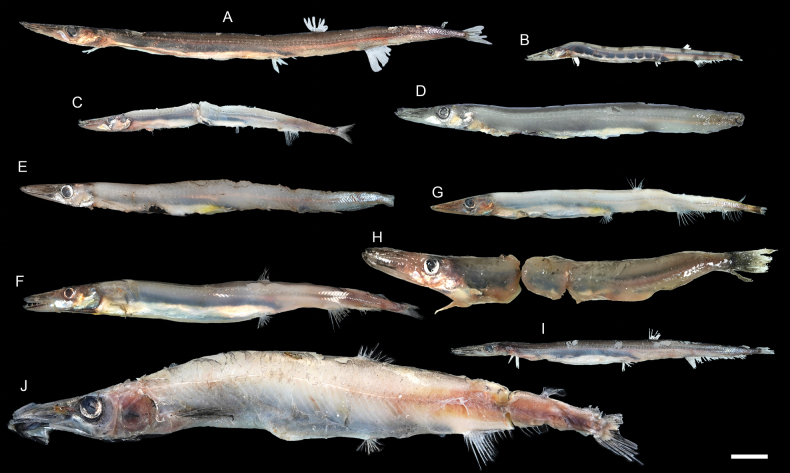
Images of fresh specimens collected around Dongsha Island **A**Stemonosudiscf.siliquiventer, 242.07 mm SL**B***Stemonosudisrothschildi*, 172.22 mm SL**C***Lestidiops* sp., 146.08 mm SL**D***Lestidiumlongilucifer*, 190.84 mm TL**E***Lestidiumprolixum*, 209.71 mm SL**F***Lestidiumorientale*, 210.45 mm SL**G***Lestrolepisphilippina*, 191.77 mm SL**H***Sudis* sp., 191.00 mm SL**I***Dolichosudisfuliginosa*, 241.49 mm SL**J***Magnisudis* sp., 318.06 mm SL. Scale bar: 3 cm.

**Figure 26.**
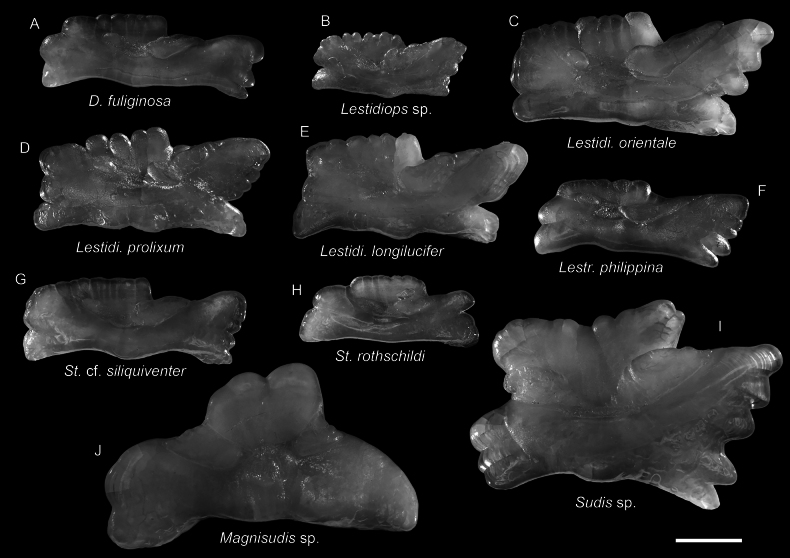
Otolith images of fresh specimens collected around Dongsha Island **A***Dolichosudisfuliginosa*, CHLOL 18720, 249.25 mm SL**B***Lestidiops* sp., CHLOL 16047, 146.08 mm SL**C***Lestidiumorientale*, CHLOL 24778, 227.03 mm SL (R) **D***Lestidiumprolixum*, CHLOL 22028, 222.87 mm SL (R) **E***Lestidiumlongilucifer*, CHLOL 20615, 199.43 mm TL (R) **F***Lestrolepisphilippina*, CHLOL 20260, 191.46 mm SL (R) **G**Stemonosudiscf.siliquiventer, CHLOL 23015, 227.89 mm SL**H***Stemonosudisrothschildi*, CHLOL 23330, 154.29 mm TL**I***Sudis* sp., CHLOL 16187, 191.00 mm SL**J***Magnisudis* sp., CHLOL22413, 294.11 mm SL. Scale bar: 1 mm.

***Lestidiumlongilucifer* Ho, Graham & Russell, 2020**

Figs 25D, 26E

This species was recently described from southern Taiwan and Australia. It is rare around Dongsha Island and Taiwan.

***Lestidiumorientale* Ho, Tsai & Li, 2019**

Figs 25F, 26C

This species, recently described around southwestern Taiwan, is restricted to the northwestern Pacific. It is rare around Dongsha Island, but common around Taiwan.

***Lestidiumprolixum* Harry, 1953**

Figs 25E, 26D

This species is restricted to the northwestern Pacific. It is rare around Dongsha Island, but common around Taiwan (Ho et al. 2019b).

***Stemonosudisrothschildi* Richards, 1967**

Figs 25B, 26H

This species is almost circumglobally distributed in tropical oceans, except eastern Pacific. Ho et al. (2019a, b) documented this species from the southern Taiwan Islands. It is common around Dongsha Island. However, it is necessary to compare the western Pacific population with their Atlantic siblings, there are significant differences between these two populations (H-CH, pers. obs.).

***Dasyscopelusselenops* (Tåning, 1928)**

Figs 27G, 29I

This species is circumglobally distributed through tropical and warm temperate oceans, except eastern Pacific. It is rare around Dongsha Island, and was recently recorded off southwestern Taiwan (Ng et al. 2024).

**Figure 27.**
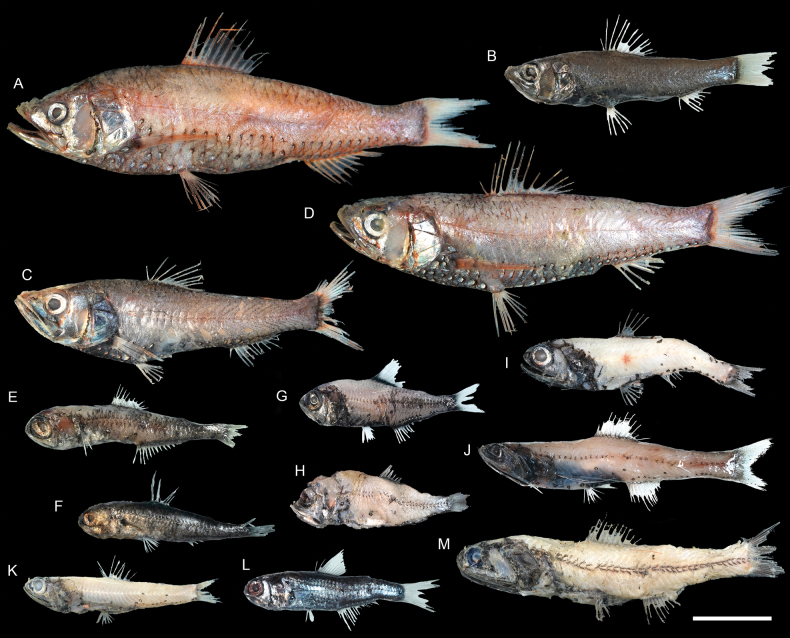
Images of fresh specimens collected around Dongsha Island **A***Neoscopelusporosus*, 147.05 mm SL**B***Neoscopelusmicrochir*, 87.87 mm SL**C***Neoscopelusmacrolepidotus*, 110.69 mm SL**D***Neoscopelus* sp., 132.66 mm SL**E***Dasyscopelusobtusirostris*, 75.20 mm SL**F***Dasyscopelusasper*, 62.70 mm SL**G***Dasyscopelusselenops*, 59.36 mm SL**H***Electronarisso*, 57.32 mm SL**I***Bolinichthys* sp., 72.95 mm SL**J***Lampanyctus* sp., 97.39 mm SL**K***Ceratoscopelus* sp., 63.30 mm SL**L***Benthosemafibulatum*, 58.90 mm SL**M***Lampadenaluminosa*, 108.84 mm SL. Scale bar: 3 cm.

***Diaphusadenomus* Gilbert, 1905**

Figs 28K, 32A

Although widespread in the Pacific and Atlantic, this species has never been reported in the tropical northwestern Pacific. The two specimens collected in the present study represent the first record in the South China Sea.

**Figure 28.**
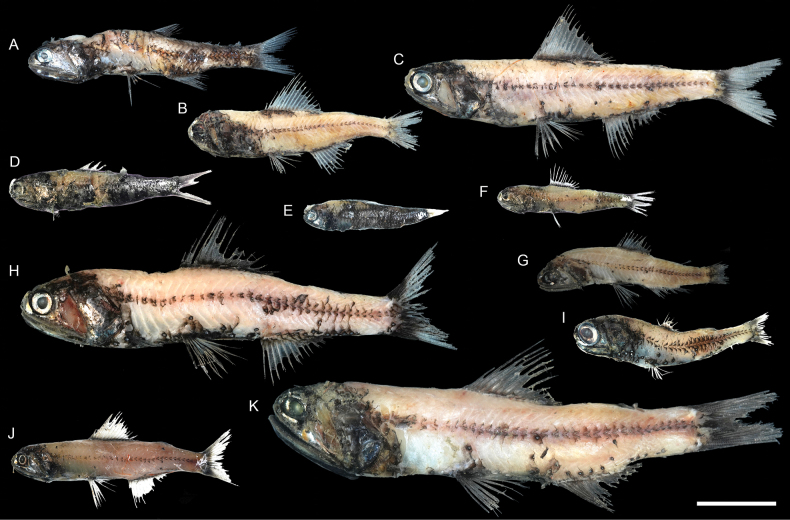
Images of fresh specimens collected around Dongsha Island **A***Diaphussplendidus*, 83.65 mm SL**B***Diaphuslucidus*, 77.38 mm SL**C***Diaphusfragilis*, 118.97 mm SL**D***Diaphusperspicillatus*, 63.58 mm SL**E***Diaphusparri*, 47.04 mm SL**F***Diaphusgarmani*, 51.20 mm SL**G***Diaphussuborbitalis*, 62.73 mm SL**H***Diaphuswatasei*, 132.33 mm SL**I***Diaphusluetkeni*, 64.93 mm SL**J***Diaphusproblematicus*, 70.60 mm SL**K***Diaphusadenomus*, 161.15 mm SL. Scale bar: 3 cm.

**Figure 29.**
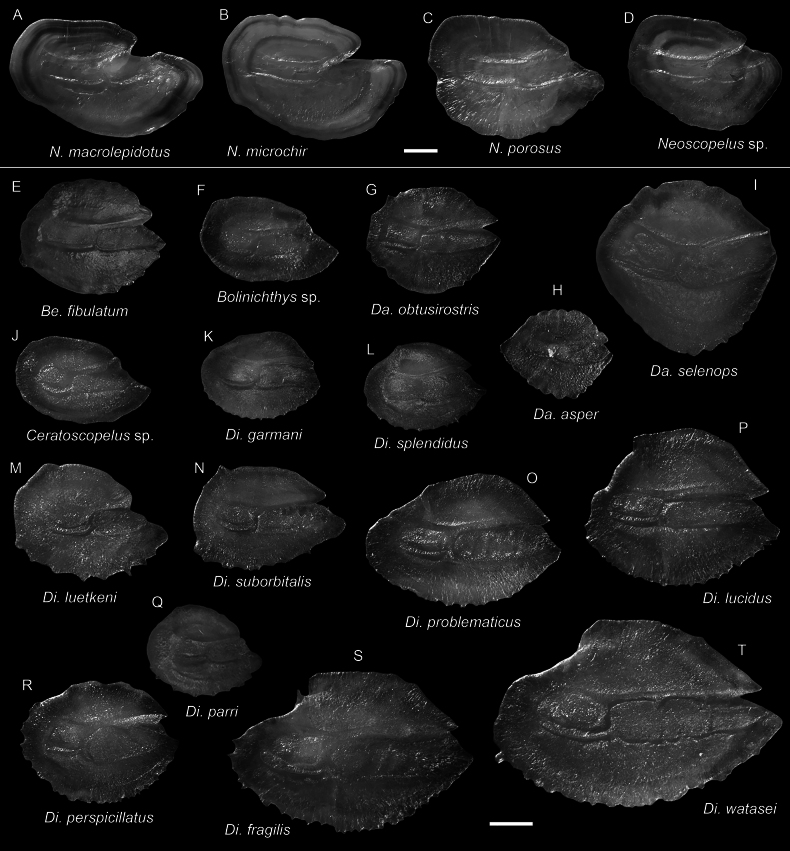
Otolith images of fresh specimens collected around Dongsha Island **A***Neoscopelusmacrolepidotus*, CHLOL 22410, 107.12 mm SL**B***Neoscopelusmicrochir*, CHLOL 20187, 107.30 mm SL**C***Neoscopelusporosus*, CHLOL 20608, 129.94 mm SL**D***Neoscopelus* sp., CHLOL 20517, 83.60 mm SL**E***Benthosemafibulatum*, CHLOL 9210, 57.15 mm SL**F***Bolinichthys* sp., CHLOL 29700, 60.66 mm SL**G***Dasyscopelusobtusirostris*, CHLOL 22678, 75.20 mm SL**H***Dasyscopelusasper*, CHLOL 24112, 62.13 mm SL**I***Dasyscopelusselenops*, CHLOL 20183, 63.61 mm SL**J***Ceratoscopelus* sp., CHLOL 20651 61.32 mm SL**K***Diaphusgarmani*, CHLOL 22594, 48.91 mm SL**L***Diaphussplendidus*, CHLOL 29230, 33.81 mm SL**M***Diaphusluetkeni*, CHLOL 17523, 64.93 mm SL**N***Diaphussuborbitalis*, CHLOL 22680, 73.40 mm SL**O***Diaphusproblematicus*, CHLOL 22774, 81.69 mm SL**P***Diaphuslucidus*, CHLOL 16019, 91.21 mm SL**Q***Diaphusparri*, CHLOL 20570, 47.04 mm SL**R***Diaphusperspicillatus*, CHLOL 22485, 56.48 mm SL**S***Diaphusfragilis*, CHLOL 20604, 108.61 mm SL**T***Diaphuswatasei*, CHLOL 3480, 114.45 mm SL. Scale bars: 1 mm.

***Zenionjaponicum* Kamohara, 1934**

Figs 30I, 32N

This species is widespread in the Pacific. Only one specimen was collected around Dongsha Island, yet it appears to be more common around Taiwan.

**Figure 30.**
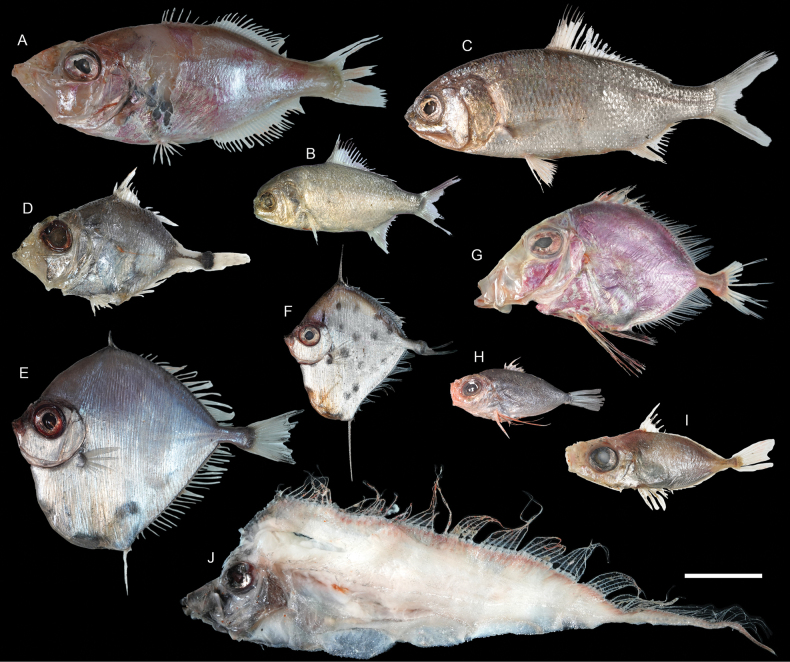
Images of fresh specimens collected around Dongsha Island **A***Parazenpacificus*, 89.49 mm SL**B***Polymixialongispina*, 64.33 mm SL**C***Polymixiaberndti*, 115.2 mm SL**D***Cyttomimusaffinis*, 75.63 mm SL**E**Xenolepidichthyscf.dalgleishi, 90.42 mm SL**F***Xenolepidichthysdalgleishi*, 51.16 mm SL**G***Cyttopsisrosea*, 147.47 mm SL**H***Zenion* sp., 42.07 mm SL**I***Zenionjaponicum*, 61.92 mm SL**J***Zucristatus*, 216.98 mm SL. Scale bar: 3 cm.

**Figure 31.**
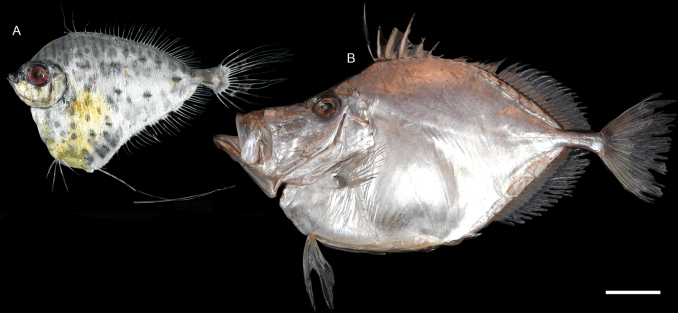
Images of fresh specimens collected around Dongsha Island **A***Grammicolepisbrachiusculus*, 159.37 mm SL**B***Zenopsisnebulosa*, 378.54 mm SL. Scale bar: 3 cm.

**Figure 32.**
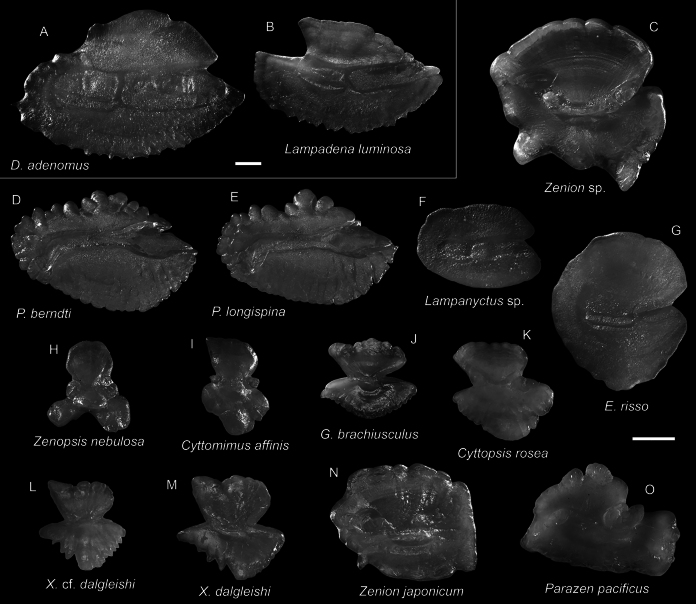
Otolith images of fresh specimens collected around Dongsha Island **A***Diaphusadenomus*, CHLOL 29085, 161.15 mm SL**B***Lampadenaluminosa*, CHLOL 17521, 89.71 mm SL**C***Zenion* sp., CHLOL 30182, 82.69 mm SL (R) **D***Polymixiaberndti*, CHLOL 29480, 71.65 mm SL**E***Polymixialongispina*, CHLOL 20312, 76.05 mm SL**F***Lampanyctus* sp., CHLOL 18810, 97.39 mm SL (R) **G***Electronarisso*, CHLOL 16387, 57.32 mm SL**H***Zenopsisnebulosa*, CHLOL 5787, 378.54 mm SL**I***Cyttomimusaffinis*, CHLOL 23366, 56.18 mm SL**J***Grammicolepisbrachiusculus*, CHLOL 20638, 140.27 mm SL**K***Cyttopsisrosea*, CHLOL 29192, 99.27 mm SL**L**Xenolepidichthyscf.dalgleishi, CHLOL 17498, 97.39 mm SL**M***Xenolepidichthysdalgleishi*, CHLOL 21836, 104.22 mm SL**N***Zenionjaponicum*, CHLOL 30180, 61.92 mm SL (R) **O***Parazenpacificus*, CHLOL 18765, 122.83 mm SL. Scale bars: 1 mm.

***Coelorinchusmacrorhynchus* Smith & Radcliffe, 1912**

Figs 33E, 34H

This species is widespread from southwestern Taiwan to western Australia, but has never been recorded around Dongsha Island, until the present study. It is not uncommon around Dongsha Island, yet most of the specimens collected are small. The largest specimen examined was 302 mm TL.

**Figure 33.**
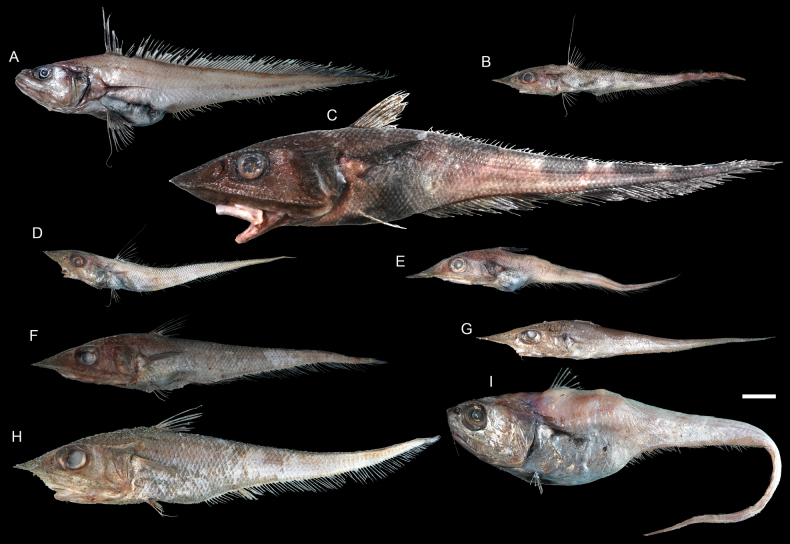
Images of fresh specimens collected around Dongsha Island **A***Gadomuscolletti*, 114.77 mm PAL**B***Coelorinchuscingulatus*, 88.53 mm PAL**C***Coelorinchussheni*, 184.66 mm PAL**D***Coelorinchusbrevirostris*, 76.92 mm PAL**E***Coelorinchusmacrorhynchus*, 80.41 mm PAL**F***Coelorinchus* sp., 125.80 mm PAL**G***Coelorinchuslongissimus*, 95.10 mm PAL**H***Coelorinchussmithi*, 138.37 mm PAL**I***Malacocephalusnipponensis*, 100.03 mm PAL. Scale bar: 3 cm.

**Figure 34.**
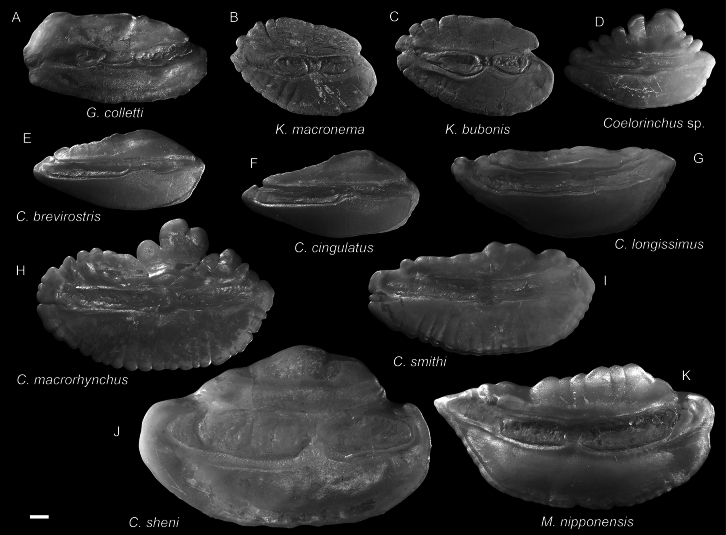
Otolith images of fresh specimens collected around Dongsha Island **A***Gadomuscolletti*, CHLOL 20823, 103.93 mm PAL**B***Kuronezumiamacronema*, CHLOL 27490, 208.00 mm TL (R) **C***Kuronezumiabubonis*, CHLOL 27491, 51.66 mm PAL**D***Coelorinchus* sp., CHLOL 20068, 112.86 mm PAL**E***Coelorinchusbrevirostris*, CHLOL 15969, 83.09 mm PAL**F***Coelorinchuscingulatus*, CHLOL 17475, 79.90 mm PAL**G***Coelorinchuslongissimus*, CHLOL 15397, 105.80 mm PAL**H***Coelorinchusmacrorhynchus*, CHLOL 15763, 126.55 mm PAL**I***Coelorinchussmithi*, CHLOL 17376, 125.91 mm PAL**J***Coelorinchussheni*, CHLOL 19815, 214.53 mm PAL**K***Malacocephalusnipponensis*, CHLOL 28535, 106.91 mm PAL. Scale bar: 1 mm.

***Coelorinchussheni* Chiou, Shao & Iwamoto, 2004**

Figs 33C, 34J

Only one specimen was collected from Dongsha Island. It is also very rare around Taiwan and Japan (Nakayama 2020). The present record is a distributional range extension from southeastern Taiwan. This species inhabits rocky bottoms in deep waters, where trawl fisheries rarely operate (Iwamoto et al. 2015).

***Coelorinchussmithi* Gilbert & Hubbs, 1920**

Figs 33H, 34I

This species is widely distributed in the central Indo-West Pacific, and is rare around Dongsha Island and Taiwan. Nakayama (2020) noted different body colorations between specimens from the Philippines and Japan. The specimens around Dongsha Island resemble those from Japan, having the ventral side of the body distinctly paler than the lateral side.

***Kuronezumiabubonis* Iwamoto, 1974**

Figs 34C, 37A

This species has a scattered distribution in the western Atlantic, western Indian Ocean, and the southwestern Pacific. Only one specimen was collected around Dongsha Island, representing the first record from the northwestern Pacific.

***Spicomacruruskuronumai* (Kamohara, 1938)**

Figs 36L, 37K

This species was previously known only from Japan to Taiwan. The present record around Dongsha Island suggests its broad distribution in the northwestern Pacific.

**Figure 35.**
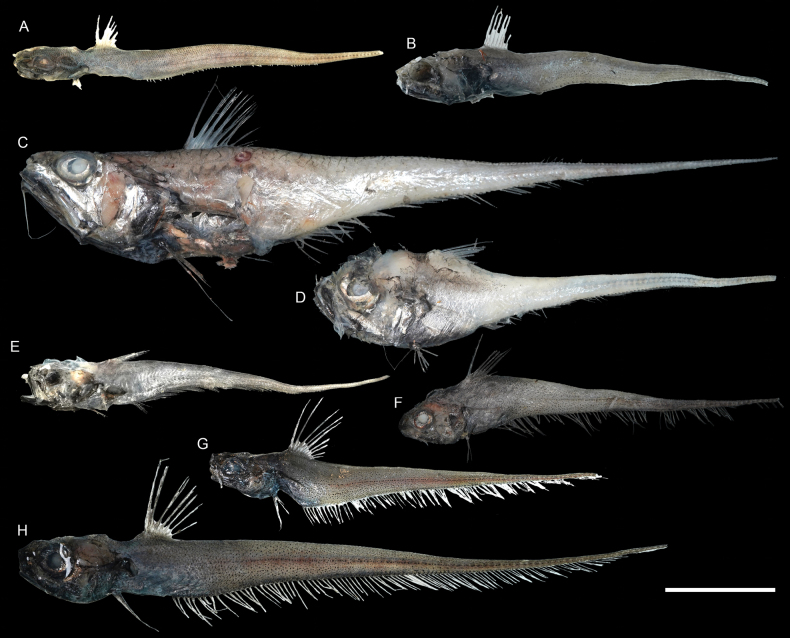
Images of fresh specimens collected around Dongsha Island **A***Sphagemacruruspumiliceps*, 23.47 mm PAL**B***Hymenocephaluslethonemus*, 37.27 mm PAL**C***Hymenocephaluslongibarbis*, 199.32 mm TL**D***Hymenocephalusstriatissimus*, 39.66 mm PAL**E**Pseudocetonuruscf.septifer, 25.13 mm PAL**F***Kumbagymnorhynchus*, 22.12 mm PAL**G***Kumbajaponica*, 18.44 mm PAL**H***Kumbapunctulata*, 21.27 mm PAL. Scale bar: 3 cm.

**Figure 36.**
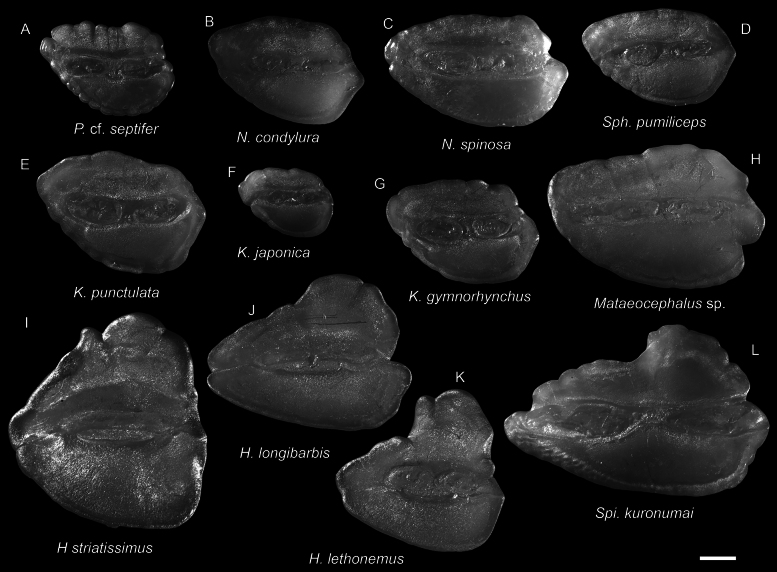
Otolith images of fresh specimens collected around Dongsha Island **A**Pseudocetonuruscf.septifer, CHLOL 22917, 25.13 mm PAL**B***Nezumiacondylura*, CHLOL 28936, 27.50 mm PAL**C***Nezumiaspinosa*, CHLOL 28000, 38.12 mm PAL**D***Sphagemacruruspumiliceps*, CHLOL 20138, 23.13 mm PAL**E***Kumbapunctulata*, CHLOL 20826, 34.22 mm PAL**F***Kumbajaponica*, CHLOL 29704, 15.49 mm PAL**G***Kumbagymnorhynchus*, CHLOL 27679, 22.12 mm PAL**H***Mataeocephalus* sp., CHLOL 28763, 156.82 mm TL**I***Hymenocephalusstriatissimus*, CHLOL 20135, 33.43 mm PAL**J***Hymenocephaluslongibarbis*, CHLOL 20012, 58.81 mm PAL**K***Hymenocephaluslethonemus*, CHLOL 20137, 39.68 mm PAL**L***Spicomacruruskuronumai*, CHLOL 23002, 52.10 mm PAL. Scale bar: 1 mm.

**Figure 37.**
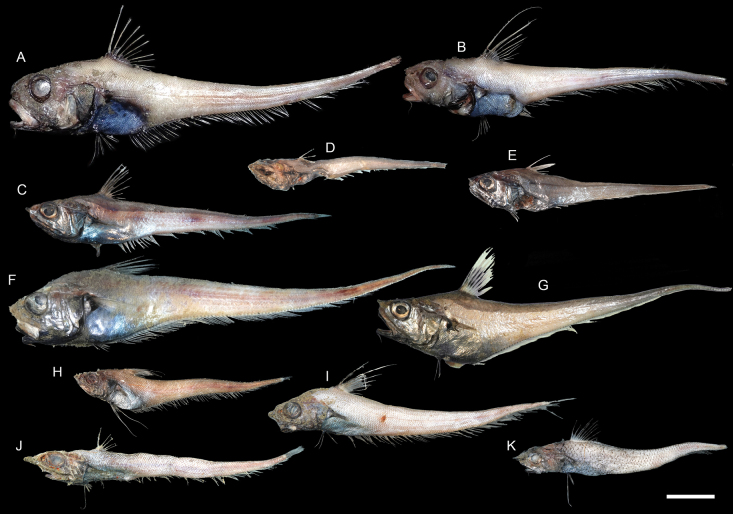
Images of fresh specimens collected around Dongsha Island **A***Kuronezumiabubonis*, 51.66 mm PAL**B***Kuronezumiamacronema*, 208 mm TL**C***Ventrifossalongibarbata*, 41.13 mm PAL**D***Ventrifossajohnboborum*, 35.24 mm PAL**E***Ventrifossasazonovi*, 166.76 mm TL**F***Ventrifossadivergens*, 56.05 mm PAL**G**Ventrifossacf.longibarbata, 52.96 mm PAL**H***Nezumiacondylura*, 30.56 mm PAL**I***Nezumiaspinosa*, 45.92 mm PAL**J***Mataeocephalus* sp., 54.96 mm PAL**K***Spicomacruruskuronumai*, 37.02 mm PAL. Scale bar: 3 cm.

***Ventrifossajohnboborum* Iwamoto, 1982**

Figs 37D, 38A

This species is widespread in the central Indo-West Pacific and the southeastern Pacific. Only two small individuals were collected around Dongsha Island.

**Figure 38.**
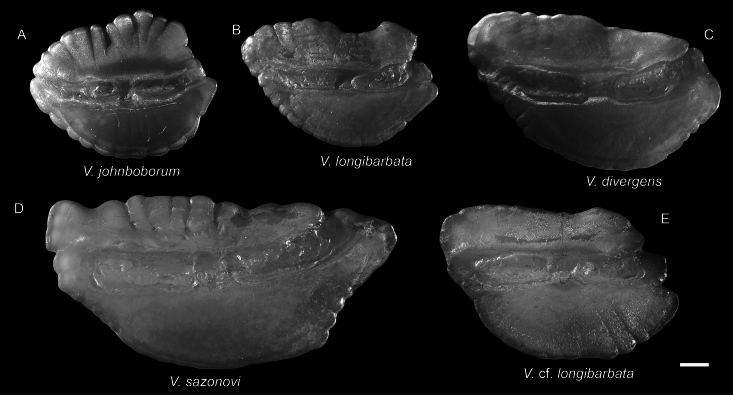
Otolith images of fresh specimens collected around Dongsha Island **A***Ventrifossajohnboborum*, CHLOL 27105, 35.24 mm PAL**B***Ventrifossalongibarbata*, CHLOL 22754, 48.88 mm PAL**C***Ventrifossadivergens*, CHLOL 25656, 69.58 mm PAL**D***Ventrifossasazonovi*, CHLOL 20707, 56.86 mm PAL**E**Ventrifossacf.longibarbata, CHLOL 17064, 47.15 mm PAL. Scale bar: 1 mm.

***Gadellajordani* (Böhlke & Mead, 1951)**

Figs 39D, 40A

This species is widespread in the Indo-West Pacific and is one the most common species in deepwater trawl fisheries around Taiwan. However, this species appears to be less common around Dongsha Island.

**Figure 39.**
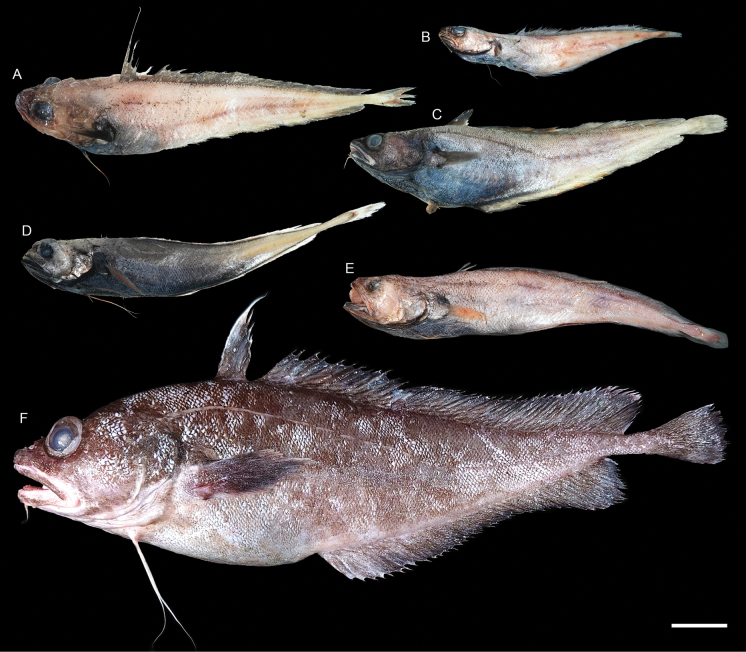
Images of fresh specimens collected around Dongsha Island **A***Physiculuschigodarana*, 211.21 mm TL**B***Physiculus* sp. 1, 129.64 mm TL**C***Physiculusjaponicus*, 203.06 mm TL**D***Gadellajordani*, 192.16 mm TL**E***Physiculus* sp. 2, 195.32 mm TL**F***Laemonemarobustum*, 391.00 mm TL. Scale bar: 3 cm.

**Figure 40.**
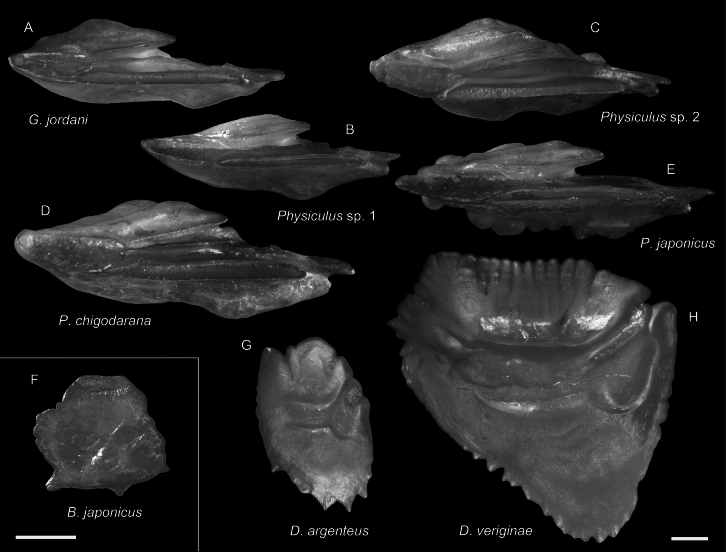
Otolith images of fresh specimens collected around Dongsha Island **A***Gadellajordani*, CHLOL 27463, 205.96 mm SL (R) **B***Physiculus* sp. 1, CHLOL 27649, 112.77 mm SL**C***Physiculus* sp. 2, CHLOL 4203, 56.27 mm PAL**D***Physiculuschigodarana*, CHLOL 22372, 168.71 mm SL**E***Physiculusjaponicus*, CHLOL 18348, 57.47 mm PAL**F***Bregmacerosjaponicus*, CHLOL 15755, 62.54 mm SL**G***Diretmusargenteus*, CHLOL 22570, 52.10 mm SL. **H***Diretmoidesveriginae*, CHLOL 18692, 118.47 mm SL Scale bars: 1 mm.

***Laemonemarobustum* Johnson, 1862**

Fig. 39F

This species has a circumglobal distribution in tropical and temperate oceans. It is known from one specimen captured by a deepsea longline fishery around Dongsha Island.

***Physiculuschigodarana* Paulin, 1989**

Figs 39A, 40D

This species is restricted to the northwestern Pacific. It is common around Taiwan but rare around Dongsha Island. Based on our observations, this species usually occurs in shallower waters.

***Hoplostethusroseus* Su, Lin & Ho, 2022**

Figs 41G, 42D

This species was recently described from Taiwan and is widespread in the central Indo-West Pacific. We found that this species is not uncommon around Dongsha Island, suggesting its broad distribution in the South China Sea.

**Figure 41.**
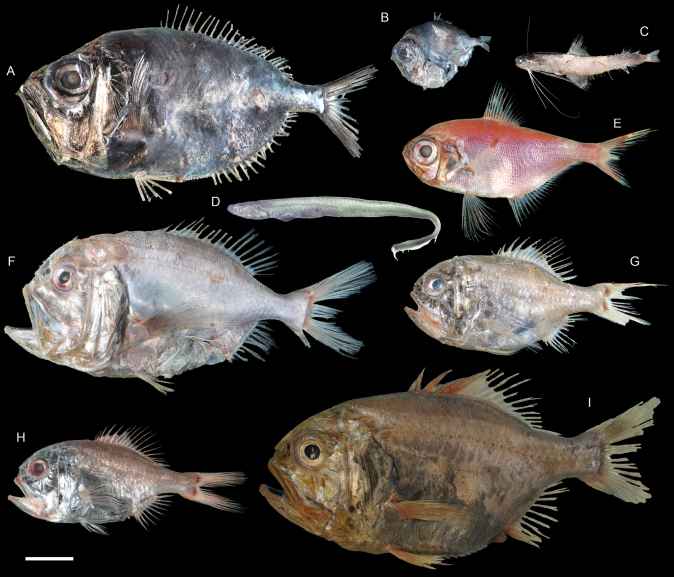
Images of fresh specimens collected around Dongsha Island **A***Diretmoidesveriginae*, 157.03 mm SL**B***Diretmusargenteus*, 51.84 mm SL**C***Bregmacerosjaponicus*, 82.38 mm SL**D***Encheliophis* sp., 165.00 mm SL**E***Beryxmollis*, 118.03 mm SL**F***Hoplostethusmelanopus*, 174.52 mm SL**G***Hoplostethusroseus*, 110.34 mm SL**H***Hoplostethus* sp., 107.77 mm SL**I***Hoplostethusrobustispinus*, 213.97 mm SL. Scale bar: 3 cm.

**Figure 42.**
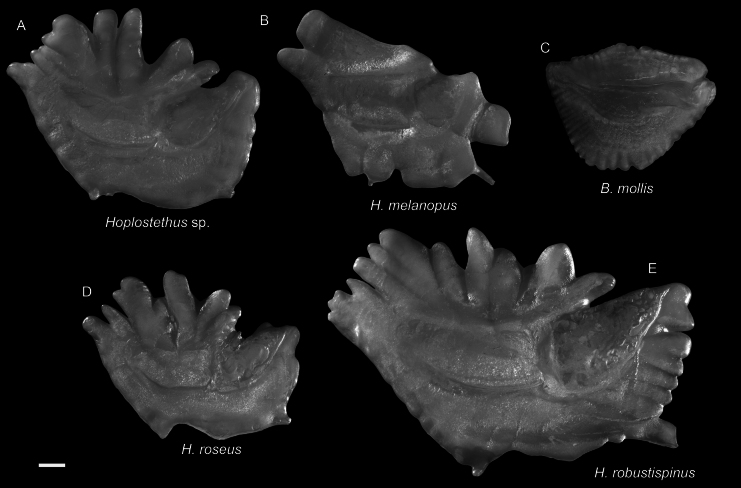
Otolith images of fresh specimens collected around Dongsha Island **A***Hoplostethus* sp., CHLOL 29039, 140.17 mm SL**B***Hoplostethusmelanopus*, CHLOL 20387, 157.22 mm SL**C***Beryxmollis*, CHLOL 20633, 122.49 mm SL**D***Hoplostethusroseus*, CHLOL 22715, 110.74 mm SL**E***Hoplostethusrobustispinus*, CHLOL 22731, 213.97 mm SL. Scale bar: 1 mm.

***Glyptophidiumargenteum* Alcock, 1889**

Figs 43I, 44G

This species has a broad distribution in the Indo-West Pacific. It is rare around Taiwan and Dongsha Island, where only one specimen was collected.

**Figure 43.**
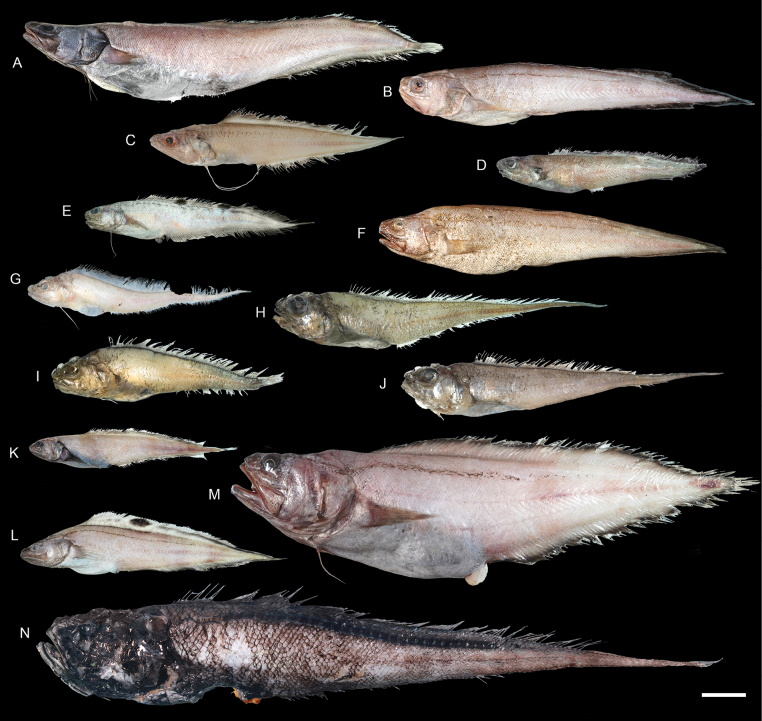
Images of fresh specimens collected around Dongsha Island **A***Luciobrotulabartschi*, 133.17 mm PAL**B***Hoplobrotulaarmata*, 81.73 mm PAL**C***Homostolusacer*, 68.27 PAL**D***Monomitopus* sp., 58.58 mm PAL**E***Neobythitesbimaculatus*, 50.38 mm PAL**F***Neobythitesunimaculatus*, 96.75 mm PAL**G***Glyptophidiumjaponicum*, 56.51 mm PAL**H***Glyptophidiumlucidum*, 74.65 mm PAL**I***Glyptophidiumargenteum*, 65.90 mm PAL**J***Glyptophidiumoceanium*, 77.92 mm PAL**K***Dicrolenetristis*, 51.33 PAL**L***Neobythiteslongipes*, 51.33 mm PAL**M***Pycnocraspedummicrolepis*, 142.31 mm PAL**N***Lamprogrammusbrunswigi*, 440.00 mm TL. Scale bar: 3 cm.

**Figure 44.**
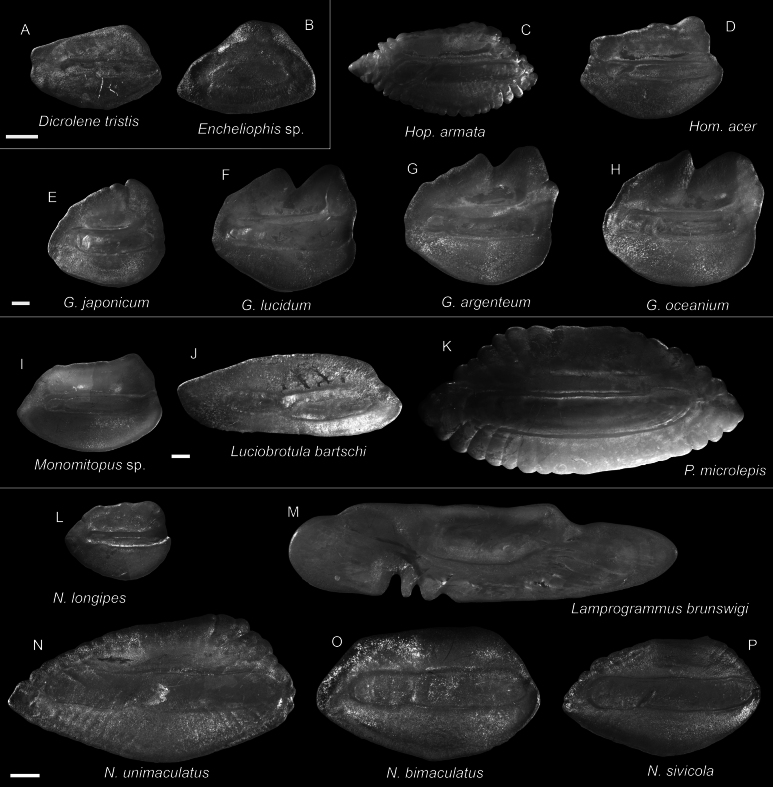
Otolith images of fresh specimens collected around Dongsha Island **A***Dicrolenetristis*, CHLOL 20052, 63.84 mm PAL**B***Encheliophis* sp., CHLOL 4101, 165.00 mm TL**C***Hoplobrotulaarmata*, CHLOL 24857, 196.93 mm SL**D***Homostolusacer*, CHLOL 20168, 73.17 mm PAL**E***Glyptophidiumjaponicum*, CHLOL 29128, 56.51 mm PAL**F***Glyptophidiumlucidum*, CHLOL 23426, 58.25 mm PAL**G***Glyptophidiumargenteum*, CHLOL 22281, 65.90 mm PAL**H***Glyptophidiumoceanium*, CHLOL 19990, 77.92 mm PAL. **I***Monomitopus* sp., CHLOL 22272, 59.58 mm PAL**J***Luciobrotulabartschi*, CHLOL 20829, 231.74 mm SL**K***Pycnocraspedummicrolepis*, CHLOL 15786, 142.31 mm PAL. **L***Neobythiteslongipes*, CHLOL 22758, 68.89 mm SL**M***Lamprogrammusbrunswigi*, CHLOL 17964, 440.00 mm TL**N***Neobythitesunimaculatus*, CHLOL 6266, 88.86 mm PAL**O***Neobythitesbimaculatus*, CHLOL 9881, 57.53 mm PAL**P***Neobythitessivicola*, CHLOL 27431, 48.80 mm PAL. Scale bars: 1 mm.

***Glyptophidiumjaponicum* Kamohara, 1936**

Figs 43G, 44E

Similar to the congeners, this species can be found in the Indo-West Pacific. It appears more common than the other sympatric congeners around Dongsha Island and Taiwan.

***Glyptophidiumoceanium* Smith & Radcliffe in Radcliffe, 1913**

Figs 43J, 44H

As in other congeners, this species is widespread in the Indo-West Pacific. It is rare around Dongsha Island and around Taiwan.

***Neobythitesbimaculatus* Nielsen, 1997**

Figs 43E, 44O

Being widespread in the central Indo-West Pacific, this species is very common around Dongsha Island, but less common around Taiwan. It is especially similar to *N.macrops* Günther, 1887 described from the Philippines. However, *N.macrops* has not been reported in the South China Sea before. Therefore, all specimens are identified as *N.bimaculatus*.

***Neobythitesunimaculatus* Smith & Radcliffe in Radcliffe, 1913**

Figs 43F, 44N

This species is widespread in the central Indo-West Pacific. It is known only from one specimen around Dongsha Island but appears to be more common around the shallower waters of Taiwan. The depth record around Dongsha Island appears to be the lower depth limit of this species.

***Cataetyxlepidogenys* (Smith & Radcliffe, 1913)**

Figs 45F, 46G

This species is restricted to the northwestern Pacific. While it is common off southwestern Taiwan, we only collected a few specimens around Dongsha Island.

**Figure 45.**
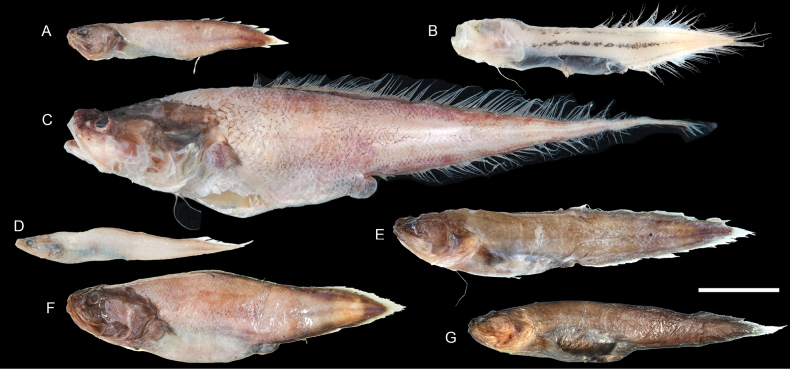
Images of fresh specimens collected around Dongsha Island **A**Bythitidae indet., 42.50 mm PAL**B***Barathronusmaculatus*, 69.18 mm PAL**C***Diplacanthopoma* sp., 103.86 mm PAL**D***Pseudonussquamiceps*, 41.47 mm PAL**E***Saccogasterhorrida*, 58.20 mm PAL**F***Cataetyxlepidogenys*, 71.10 mm PAL**G***Saccogastertuberculata*, 59.34 mm PAL. Scale bar: 3 cm.

**Figure 46.**
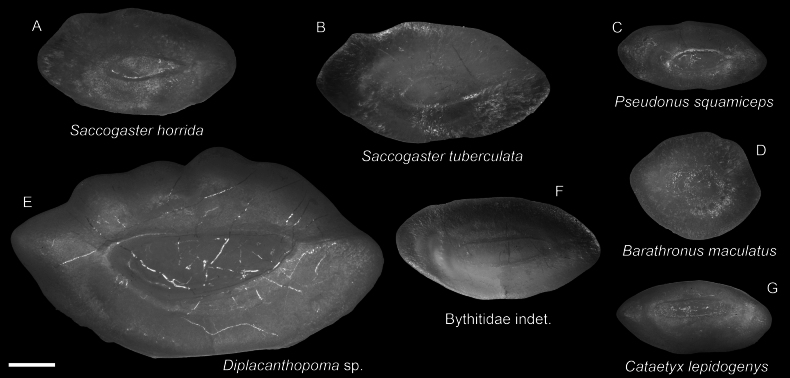
Otolith images of fresh specimens collected around Dongsha Island **A***Saccogasterhorrida*, CHLOL 18127, 55.20 mm PAL**B***Saccogastertuberculata*, CHLOL 22284, 59.34 mm PAL**C***Pseudonussquamiceps*, CHLOL 19987, 37.55 mm PAL**D***Barathronusmaculatus*, CHLOL 25717, 69.18 mm SL**E***Diplacanthopoma* sp., CHLOL 19402, 76.62 mm PAL**F**Bythitidae indet., CHLOL 19985, 42.50 mm PAL (R) **G***Cataetyxlepidogenys*, CHLOL 18149, 71.10 mm PAL. Scale bar: 1 mm.

***Pseudonussquamiceps* (Lloyd, 1907)**

Figs 45D, 46C

This species is found in the central Indo-West Pacific. Although poorly presented in museums, we found that it is not uncommon around Dongsha Island.

***Saccogasterhorrida* Nielsen, Schwarzhans & Cohen, 2012**

Figs 45E, 46A

This species was known only from western Australia. The sole specimen collected around Dongsha Island in the present study suggests a potential broad distribution in the central Indo-West Pacific.

***Barathronusmaculatus* Shcherbachev, 1976**

Figs 45B, 46D

This species is widespread in the Indo-West Pacific. It is rare around Dongsha Island, as it usually occurs in much deeper waters (e.g. Nielsen and Machida 1985; Yeh et al. 2009).

**Figure 47.**
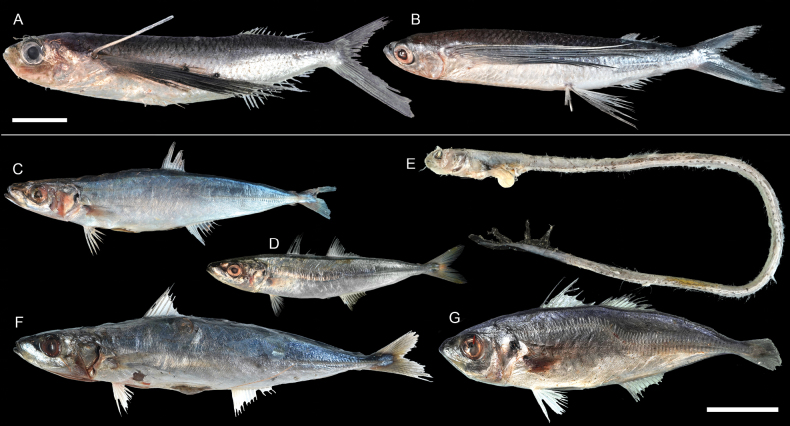
Images of fresh specimens collected around Dongsha Island **A***Exocoetusmonocirrhus*, 144.25 mm SL**B***Hirundichthysoxycephalus*, 211.01 mm TL**C***Decapterusmacrosoma*, 124.06 mm SL**D***Decapterustabl*, 91.56 mm SL**E***Xiphasiasetifer*, 312.83 mm TL**F***Decapterusmacarellus*, 174.18 mm TL**G***Trachurusjaponicus*, 125.57 mm SL. Scale bars: 3 cm.

**Figure 48.**
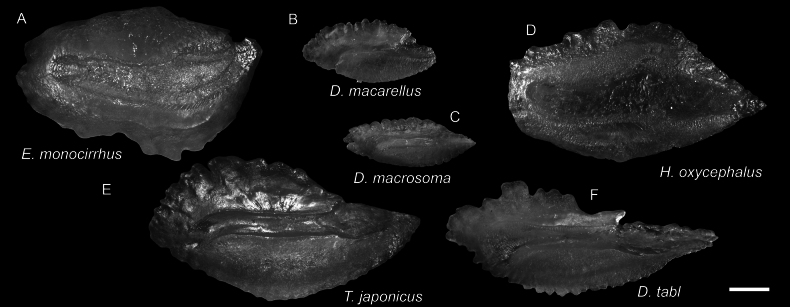
Otolith images of fresh specimens collected around Dongsha Island **A***Exocoetusmonocirrhus*, CHLOL 19408, 144.25 mm SL**B***Decapterusmacarellus*, CHLOL 20653, 174.18 mm TL**C***Decapterusmacrosoma*, CHLOL 17765, 124.06 mm SL (R) **D***Hirundichthysoxycephalus*, CHLOL 15698, 211.01 mm TL**E***Trachurusjaponicus*, CHLOL 24117, 104.79 mm SL**F***Decapterustabl*, CHLOL 22730, 251.70 mm SL. Scale bar: 1 mm.

**Figure 49.**
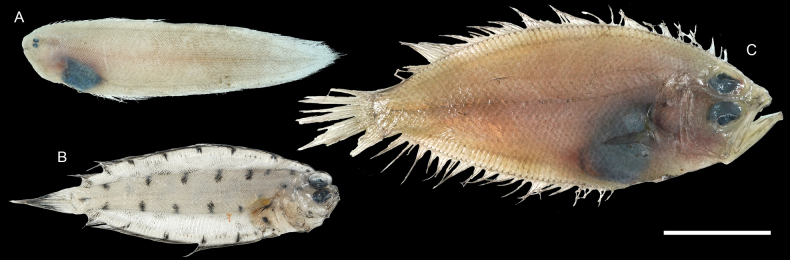
Images of fresh specimens collected around Dongsha Island **A***Symphurusorientalis*, 85.62 mm SL**B***Poecilopsettaplinthus*, 75.68 mm SL**C***Lepidoblepharonophthalmolepis*, 108.77 mm SL. Scale bar: 3 cm.

**Figure 50.**
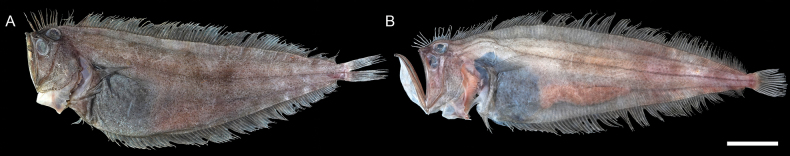
Images of fresh specimens collected around Dongsha Island **A***Chascanopsettalugubris*, 315.02 mm SL**B***Chascanopsettaprognatha*, 334.43 mm SL. Scale bar: 5 cm.

**Figure 51.**
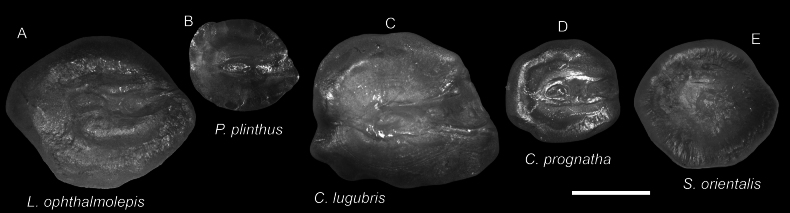
Otolith images of fresh specimens collected around Dongsha Island **A***Lepidoblepharonophthalmolepis*, CHLOL 22379, 67.13 mm SL**B***Poecilopsettaplinthus*, CHLOL 23359, 69.60 mm SL**C***Chascanopsettalugubris*, CHLOL 14976, 230.17 mm SL**D***Chascanopsettaprognatha*, CHLOL 15675, 194.29 mm SL**E***Symphurusorientalis*, CHLOL 18684, 87.69 mm SL. Scale bar: 1 mm.

**Figure 52.**
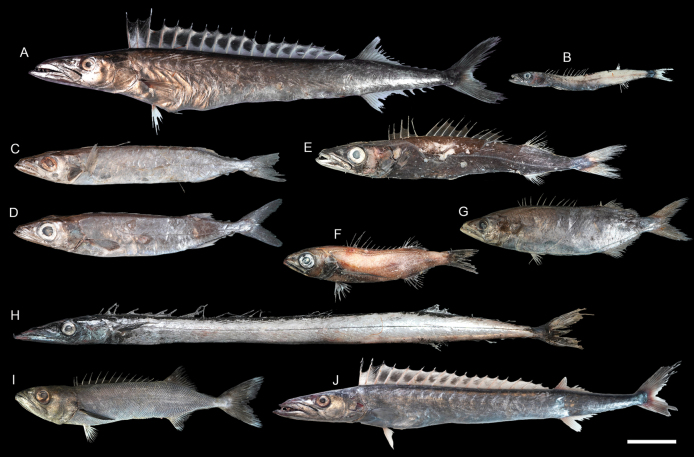
Images of fresh specimens collected around Dongsha Island **A***Nesiarchusnasutus*, 430.94 mm SL**B***Nealotustripes*, 143.09 mm SL**C***Rexeabengalensis*, 241.14 mm SL**D***Rexeaprometheoides*, 235.74 mm SL**E***Promethichthysprometheus*, 267.82 mm SL**F***Scombrolabraxheterolepis*, 161.77 mm SL**G***Neoepinnulaorientalis*, 193.94 mm SL**H***Gempylusserpens*, 506.87 mm SL**I***Ruvettuspretiosus*, 211.29 mm SL**J***Thyrsitoidesmarleyi*, 386.84 mm SL. Scale bar: 5 cm.

**Figure 53.**
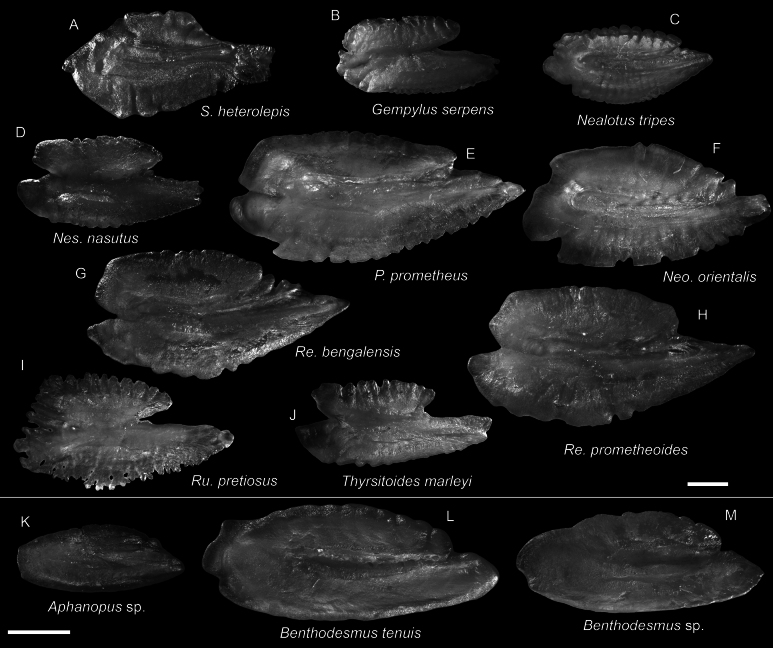
Otolith images of fresh specimens collected around Dongsha Island **A***Scombrolabraxheterolepis*, CHLOL 21499, 161.77 mm SL**B***Gempylusserpens*, CHLOL 21292, 506.87 mm SL**C***Nealotustripes*, CHLOL 18041, 143.09 mm SL**D***Nesiarchusnasutus* CHLOL 23266, 389.39 mm SL**E***Promethichthysprometheus*, CHLOL 21502, 292.37 mm SL**F***Neoepinnulaorientalis*, CHLOL 20027, 185.78 mm SL**G***Rexeabengalensis* CHLOL 5395, 241.14 mm SL**H***Rexeaprometheoides*, CHLOL 5394, 235.80 mm SL**I***Ruvettuspretiosus*, CHLOL 22035, 292.42 mm SL**J***Thyrsitoidesmarleyi*, CHLOL 24722, 390.60 mm SL**K***Aphanopus* sp., CHLOL 28066, 437.53 mm SL (R) **L***Benthodesmustenuis*, CHLOL 22846, 510.72 mm SL**M***Benthodesmus* sp., CHLOL 16539, 665.63 mm SL. Scale bars: 1 mm.

**Figure 54.**
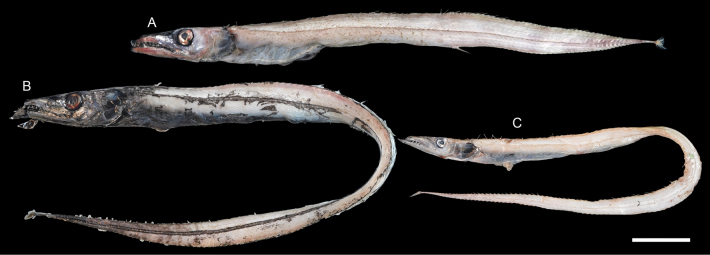
Images of fresh specimens collected around Dongsha Island **A***Aphanopus* sp., 437.53 mm SL**B***Benthodesmus* sp., 665.63 mm SL**C***Benthodesmustenuis*, 523.03 mm TL. Scale bar: 5 cm.

***Cubicepspauciradiatus* Günther, 1872**

Figs 55D, 56D

This species has circumglobal distribution in tropical and temperate oceans. It is very rare around Dongsha Island, known only from one specimen.

**Figure 55.**
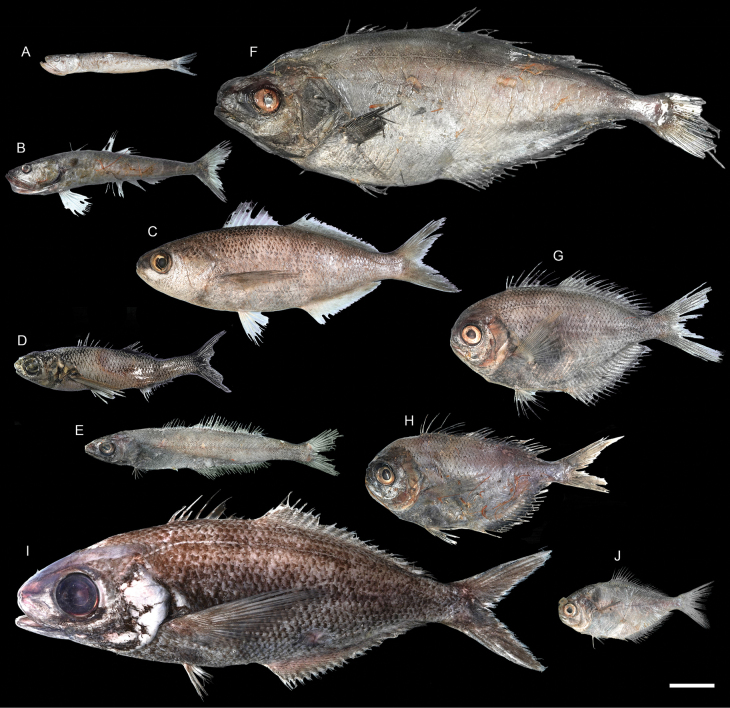
Images of fresh specimens collected around Dongsha Island **A***Champsodonsnyderi*, 90.06 mm SL**B***Champsodonlongipinnis*, 127.54 mm SL**C***Cubicepswhiteleggii*, 163.50 mm SL**D***Cubicepspauciradiatus*, 115.74 mm SL**E***Amarsipuscarlsbergi*, 146.27 mm SL**F***Psenespellucidus*, 229.58 mm SL**G***Psenescyanophrys*, 141.89 mm SL**H***Psenesarafurensis*, 123.44 mm SL**I***Cubicepsbaxteri*, 265.12 mm SL**J***Psenopsisanomala*, 76.07 mm SL. Scale bar: 3 cm.

**Figure 56.**
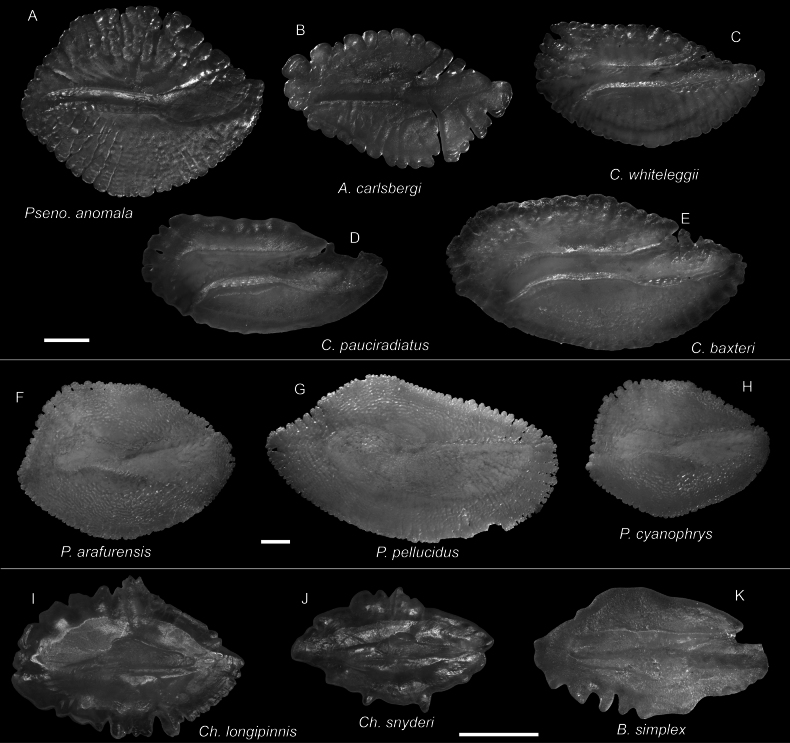
Otolith images of fresh specimens collected around Dongsha Island **A***Psenopsisanomala*, CHLOL 26057, 76.07 mm SL**B***Amarsipuscarlsbergi*, CHLOL 29706, 146.27 mm SL**C***Cubicepswhiteleggii*, CHLOL 21285, 112.40 mm SL**D***Cubicepspauciradiatus*, CHLOL 24988, 115.74 mm SL**E***Cubicepsbaxteri*, CHLOL 20390, 119.10 mm SL**F***Psenesarafurensis*, CHLOL 24971, 132.51 mm SL**G***Psenespellucidus*, CHLOL 20622, 213.19 mm SL**H***Psenescyanophrys*, CHLOL 27368, 123.32 mm SL. **I***Champsodonlongipinnis*, CHLOL 24121, 127.54 mm SL**J***Champsodonsnyderi*, CHLOL 21520, 90.06 mm SL**K***Bathysphyraenopssimplex*, CHLOL 15774, 69.53 mm SL. Scale bars: 1 mm.

***Cubicepswhiteleggii* (Waite, 1894)**

Figs 55C, 56C

Although widely distributed in the Indo-West Pacific, this species has not been recorded around Dongsha Island. Here, we found that this species is very abundant in the area, outnumbering the congeners.

***Champsodonlongipinnis* Matsubara & Amaoka, 1964**

Figs 55B, 56I

This species is widespread in the Indo-West Pacific, and is common off southwestern Taiwan. However, only one specimen was collected around Dongsha Island, which suggests that this species may prefer shallower waters.

***Malakichthyselegans* Matsubara & Yamaguti, 1943**

Figs 57D, 58H

This species is widespread in the central Indo-West Pacific, and is one of the most abundant species in bottom trawl fisheries in Taiwan. However, only one specimen was collected around Dongsha Island, possibly due to a much deeper sampling depth.

**Figure 57.**
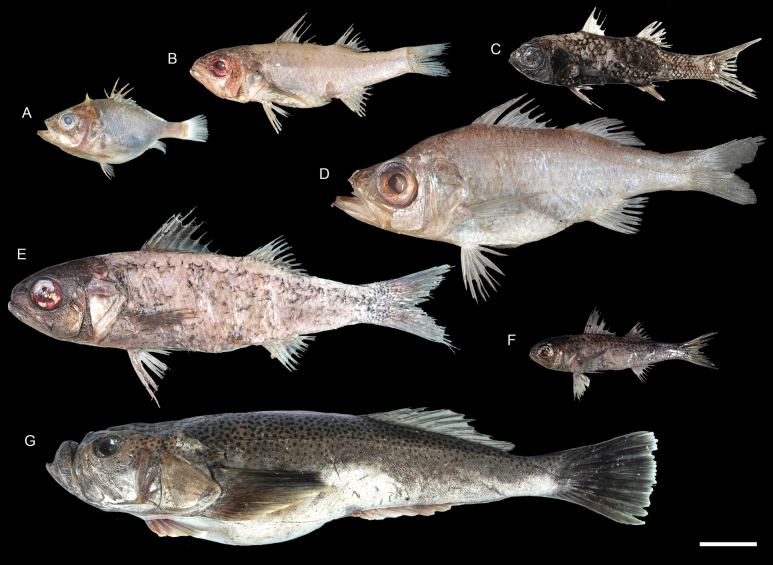
Images of fresh specimens collected around Dongsha Island **A***Ostracoberyxdorygenys*, 73.53 mm SL**B***Parascombropsserratospinosus*, 107.83 mm SL**C***Bathysphyraenopssimplex*, 69.53 mm SL**D***Malakichthyselegans*, 170.51 mm SL**E***Synagropsjaponicus*, 220.42 mm SL**F***Synagropsatrumoris*, 132.91 mm SL**G***Xenocephaluselongatus*, 219.48 mm SL. Scale bar: 5 cm.

**Figure 58.**
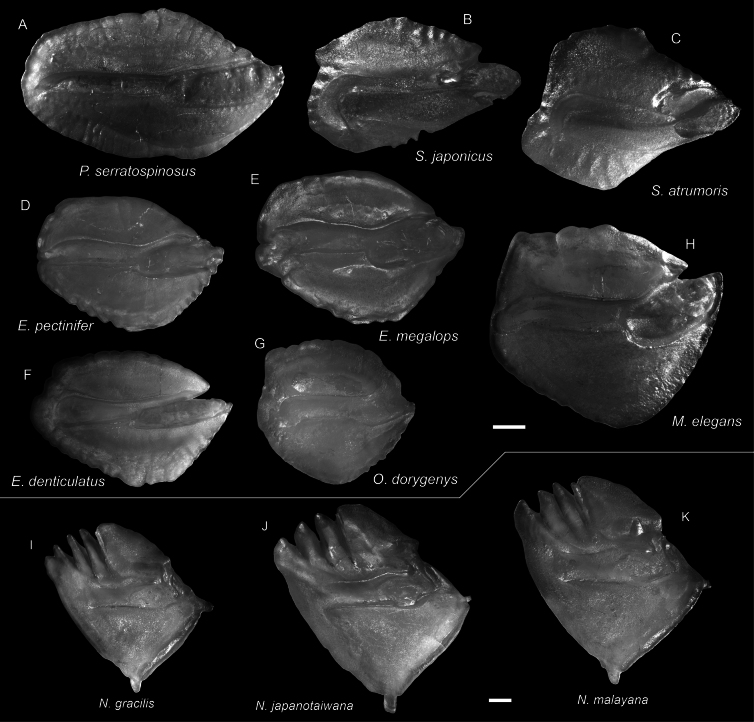
Otolith images of fresh specimens collected around Dongsha Island **A***Parascombropsserratospinosus*, CHLOL 5589, 92.81 mm SL**B***Synagropsjaponicus*, CHLOL 24379, 203.28 mm SL**C***Synagropsatrumoris*, CHLOL 26169, 129.07 mm SL**D***Epigonuspectinifer*, CHLOL 22798, 97.02 mm SL**E***Epigonusmegalops*, CHLOL 22100, 102.02 mm SL (R) **F***Epigonusdenticulatus*, CHLOL 27481, 103.79 mm SL**G***Ostracoberyxdorygenys*, CHLOL 28712, 103.95 mm SL**H***Malakichthyselegans*, CHLOL 24712, 122.29 mm SL (R). **I***Neobathyclupeagracilis*, CHLOL 17470, 159.61 mm SL**J***Neobathyclupeajapanotaiwana*, CHLOL 22536, 194.90 mm SL**K***Neobathyclupeamalayana*, CHLOL 22087, 187.50 mm SL. Scale bars: 1 mm.

***Parascombropsserratospinosus* (Smith & Radcliffe, 1912)**

Figs 57B, 58A

This species is found in the western Pacific. Although common off southwestern Taiwan, the species is very rare around Dongsha Island, which suggests that it usually occurs in shallower waters.

***Epigonusdenticulatus* Dieuzeide, 1950**

Figs 58F, 59L

This species has a circumglobal distribution in tropical and temperate oceans. It is uncommon around Dongsha Island and Taiwan.

**Figure 59.**
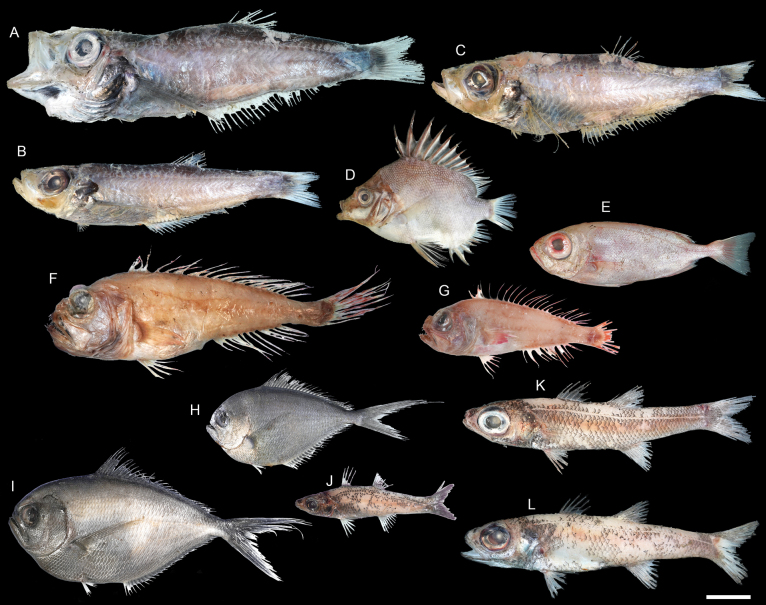
Images of fresh specimens collected around Dongsha Island **A***Neobathyclupeamalayana*, 229.75 mm SL**B***Neobathyclupeagracilis*, 179.90 mm SL**C***Neobathyclupeajapanotaiwana*, 181.32 mm SL**D***Pentacerosjaponicus*, 100.59 mm SL**E***Priacanthuszaiserae*, 124.10 mm SL**F***Owstoniagrammodon*, 183.01 mm SL**G***Owstoniaaurora*, 111.14 mm SL**H***Bramaorcini*, 102.62 mm SL**I***Bramadussumieri*, 142.96 mm SL**J***Epigonuspectinifer*, 94.61 mm SL**K***Epigonusmegalops*, 161.37 mm SL**L***Epigonusdenticulatus*, 169.85 mm SL. Scale bar: 3 cm.

***Epigonusmegalops* (Smith & Radcliffe, 1912)**

Figs 58E, 59K

This species is restricted to the Philippines. The sole specimen we recognized around Dongsha Island reflects a broad distribution in the South China Sea.

***Epigonuspectinifer* Mayer, 1974**

Figs 58D, 59J

Although widespread circumglobally in tropical and temperate oceans, this species is rare around Dongsha Island and Taiwan.

***Neobathyclupeagracilis* (Fowler, 1938)**

Figs 58I, 59B

This genus was established by Prokofiev (2014), and the species were extensively revised. It is widespread in the Indo-West Pacific, and is the most abundant bathyclupeid species around Dongsha Island and Taiwan.

***Neobathyclupeajapanotaiwana* (Prokofiev, 2014)**

Figs 58J, 59C

This species has a narrower distribution than its congeners, known only from Japan and Taiwan. However, this species is not uncommon around Dongsha Island, suggesting a broad distribution in the northwestern Pacific.

***Neobathyclupeamalayana* (Weber, 1913)**

Figs 58K, 59A

Although widespread in the Indo-West Pacific, this species is rare around Dongsha Island. It is also more prominent in body size than the two sympatric congeners.

***Bramadussumieri* Cuvier, 1831**

Figs 59I, 61B

This species can be found in tropical and warm temperate oceans. The two specimens from our collection are the first record of this species around Dongsha Island.

***Bramaorcini* Cuvier, 1831**

Figs 59H, 61C

This species is widespread in the Indo-West Pacific. It is common around Taiwan, but only one specimen was collected around Dongsha Island.

***Owstoniaaurora* Liao, Reyes & Shao, 2022**

Figs 59G, 61G

This species was recently described from the Philippines. Our present record, represented by one specimen around Dongsha Island, suggests its broad distribution in the South China Sea.

***Owstoniagrammodon* (Fowler, 1934)**

Figs 59F, 61F

This species has scattered records in Indonesia and Taiwan. The two specimens collected around Dongsha Island confirm its broader distribution in the tropical Indo-West Pacific.

***Lythrichthyscypho* (Fowler, 1938)**

Figs 60H, 62F

This species was recently revised by Wada et al. (2021). It is widely distributed in the Indo-West Pacific, and is common around Dongsha Island.

**Figure 60.**
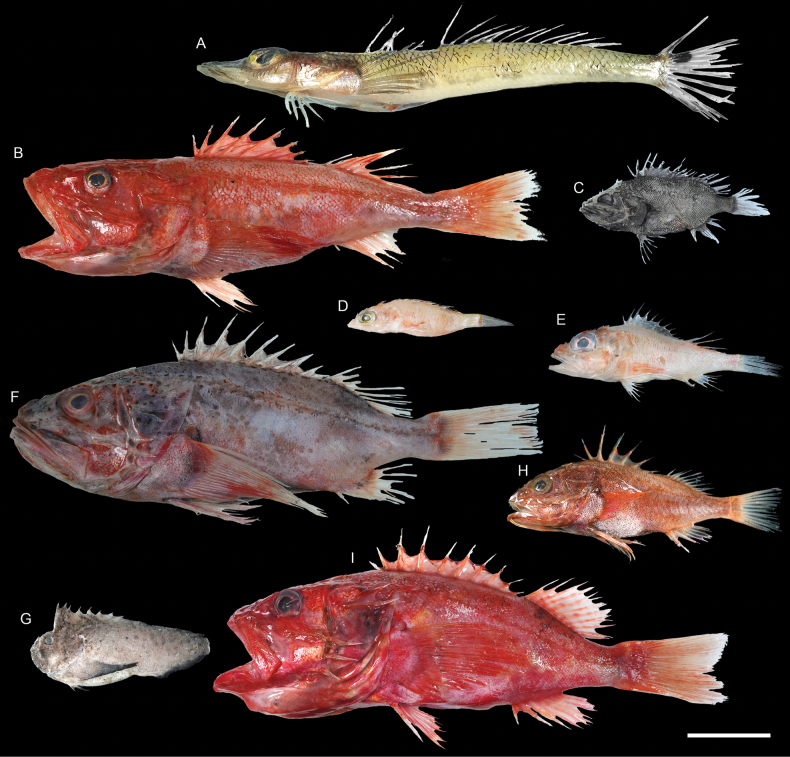
Images of fresh specimens collected around Dongsha Island **A***Bembropscaudimacula*, 165.04 mm SL**B***Lioscorpiuslongiceps*, 139.94 mm SL**C***Ectreposebastesimus*, 56.56 mm SL**D***Plectrogeniumkamoharai*, 43.27 mm SL**E***Phenacoscorpiusmegalops*, 65.03 mm SL**F***Setarchesguentheri*, 126.15 mm SL**G***Erisphexpottii*, 62.07 mm SL**H***Lythrichthyscypho*, 53.30 mm SL**I***Lythrichthyseulabes*, 137.08 mm SL. Scale bar: 3 cm.

**Figure 61.**
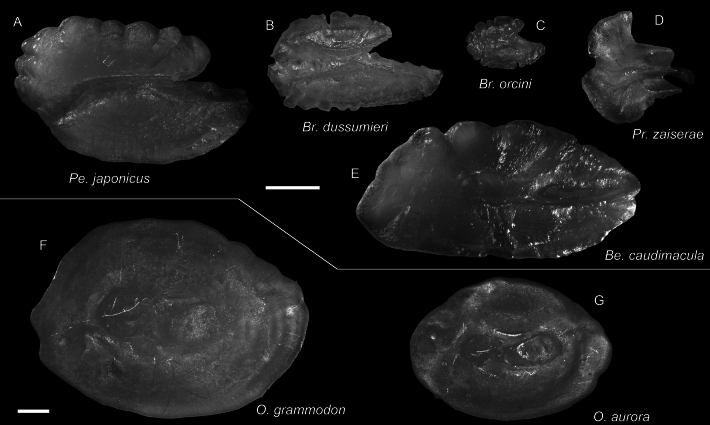
Otolith images of fresh specimens collected around Dongsha Island **A***Pentacerosjaponicus*, CHLOL 26254, 100.59 mm SL**B***Bramadussumieri*, CHLOL 25658, 142.96 mm SL**C***Bramaorcini*, CHLOL 15403, 56.19 mm SL**D***Priacanthuszaiserae*, CHLOL 5243, 124.10 mm SL (R) **E***Bembropscaudimacula*, CHLOL 22142, 165.04 mm SL**F***Owstoniagrammodon*, CHLOL 24036, 183.01 mm SL**G***Owstoniaaurora*, CHLOL 20621, 111.14 mm SL. Scale bars: 1 mm.

**Figure 62.**
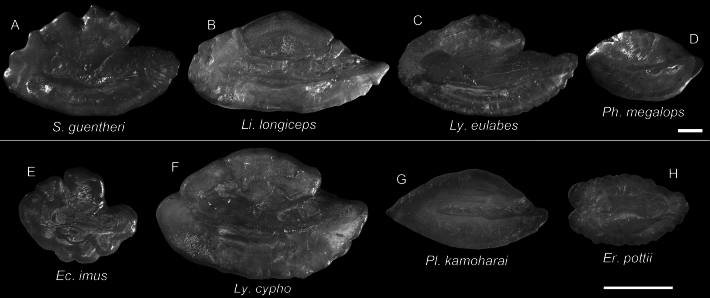
Otolith images of fresh specimens collected around Dongsha Island **A***Setarchesguentheri*, CHLOL 24292, 131.63 mm SL**B***Lioscorpiuslongiceps*, CHLOL 22373, 136.03 mm SL**C***Lythrichthyseulabes*, CHLOL 24508, 120.40 mm SL**D***Phenacoscorpiusmegalops*, CHLOL 24396, 62.60 mm SL**E***Ectreposebastesimus*, CHLOL 28795, 47.71 mm SL**F***Lythrichthyscypho*, CHLOL 24502, 47.83 mm SL**G***Plectrogeniumkamoharai*, CHLOL 9880, 49.42 mm SL**H***Erisphexpottii*, CHLOL 29164, 62.07 mm SL. Scale bars: 1 mm.

***Lythrichthyseulabes* Jordan & Starks, 1904**

Figs 60I, 62C

Being revised together with *L.cypho*, this species is also widespread in the Indo-West Pacific. It is very common around Dongsha Island and Taiwan.

***Setarchesguentheri* Johnson, 1862**

Figs 60F, 62A

This species has circumglobal distribution in tropical and subtropical oceans. It is not uncommon around Dongsha Island and Taiwan.

***Lepidotriglapectoralis* Fowler, 1938**

Figs 63F, 64F

This species is possibly endemic to the northwestern Pacific, and is rare around Dongsha Island.

**Figure 63.**
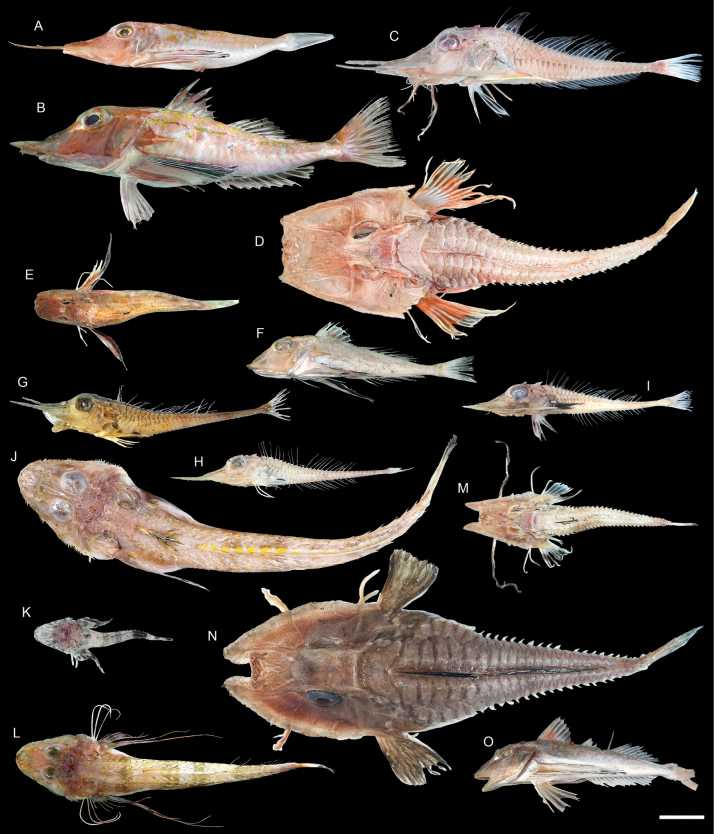
Images of fresh specimens collected around Dongsha Island **A***Pterygotriglamacrorhynchus*, 179.11 mm SL**B***Pterygotriglacajorarori*, 225.51 mm SL**C***Paraheminodusmurrayi*, 193.98 mm SL**D***Heminodusphilippinus*, 248.83 mm SL**E***Lepidotrigla* sp., 115.30 mm SL**F***Lepidotriglapectoralis*, 127.46 mm SL**G***Peristedionriversandersoni*, 158.52 mm SL**H***Peristedionorientale*, 128.99 mm SL**I***Scalicushians*, 165.47 mm SL**J***Hoplichthysgilberti*, 288.42 mm SL**K***Hoplichthysfasciatus*, 78.74 mm SL**L***Hoplichthysfilamentosus*, 184.54 mm SL**M***Scalicusorientalis*, 129.56 mm SL**N***Satyrichthysmilleri*, 265.63 mm SL**O***Chelidonichthysspinosus*, 132.61 mm. Scale bar: 3 cm.

**Figure 64.**
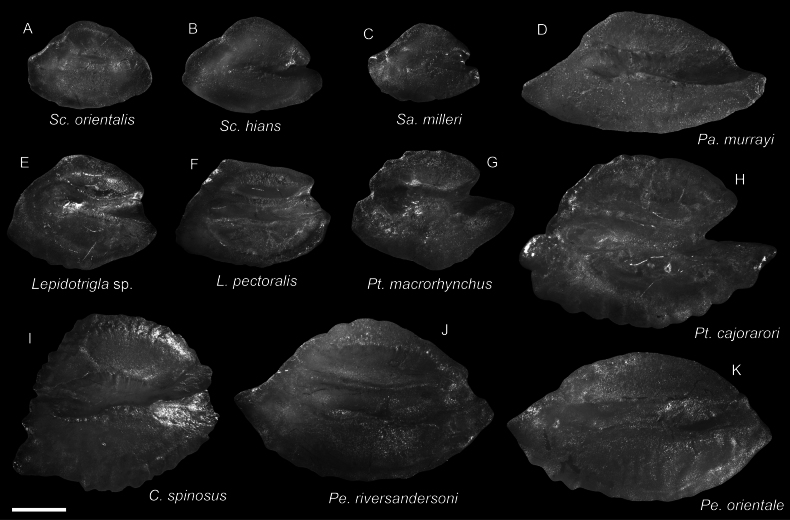
Otolith images of fresh specimens collected around Dongsha Island **A***Scalicusorientalis*, CHLOL 27465, 128.73 mm SL**B***Scalicushians*, CHLOL 29478, 165.47 mm SL**C***Satyrichthysmilleri*, CHLOL 20905, 103.55 mm SL**D***Paraheminodusmurrayi*, CHLOL 20864, 245.67 mm SL**E***Lepidotrigla* sp., CHLOL 23056, 115.30 mm SL**F***Lepidotriglapectoralis*, CHLOL 29737, 124.66 mm SL**G***Pterygotriglamacrorhynchus*, CHLOL 8397, 155.76 mm SL**H***Pterygotriglacajorarori*, CHLOL 23058, 171.52 mm SL**I***Chelidonichthysspinosus*, CHLOL 29166, 212.35 mm SL (R) **J***Peristedionriversandersoni*, CHLOL 23051, 164.13 mm SL**K***Peristedionorientale*, CHLOL 21074, 151.57 mm SL. Scale bar: 1 mm.

***Pterygotriglacajorarori* Richards & Yato, 2012**

Figs 63B, 64H

Although widespread in the Indo-West Pacific, this species appears to be uncommon around Dongsha Island.

***Paraheminodusmurrayi* (Günther, 1880)**

Figs 63C, 64D

This species is widely distributed in the Indo-West Pacific, and is common around Dongsha Island, but rare around Taiwan.

***Satyrichthysmilleri* Kawai, 2013**

Figs 63N, 64C

This species is widely distributed in the central Indo-West Pacific. It is common off southwestern Taiwan but rare around Dongsha Island.

***Hoplichthysfilamentosus* Matsubara & Ochiai, 1950**

Figs 63L, 66C

This species is widespread in the central Indo-West Pacific. It is common around Taiwan, but rare around Dongsha Island.

***Stlengisdistoechus* Bolin, 1936**

Figs 65B, 66E

This species was formerly restricted to Japan. The present record around Dongsha Island suggests its broader distribution in the northwestern Pacific.

***Psychrolutesmacrocephalus* (Gilchrist, 1904)**

Figs 65C, 66D

This species has a widespread distribution in the Indo-West Pacific. Only a few specimens were collected around Dongsha Island.

**Figure 65.**
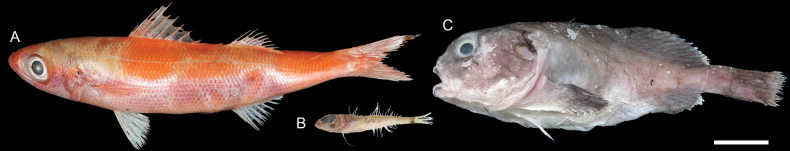
Images of fresh specimens collected around Dongsha Island **A***Erythroclesschlegelii*, 177.46 mm SL**B***Stlengisdistoechus*, 48.45 mm SL**C***Psychrolutesmacrocephalus*, 155.12 mm SL. Scale bar: 3 cm.

**Figure 66.**
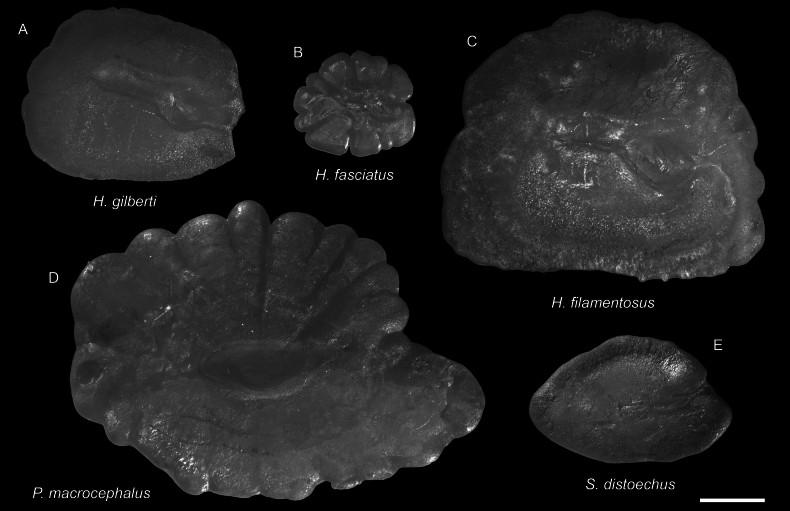
Otolith images of fresh specimens collected around Dongsha Island **A***Hoplichthysgilberti*, CHLOL 9890, 89.80 mm SL**B***Hoplichthysfasciatus*, CHLOL 24449, 78.74 mm SL**C***Hoplichthysfilamentosus*, CHLOL 22098, 204.66 mm SL**D***Psychrolutesmacrocephalus*, CHLOL 25008, 172.17 mm SL**E***Stlengisdistoechus*, CHLOL 24045, 48.77 mm SL. Scale bar: 1 mm.

***Lophiodesiwamotoi* Ho, Séret & Shao, 2011**

Figs 67A, 68B

This species was originally described from off Society Islands. A few specimens were collected around Dongsha Island, representing a substantial range extension.

**Figure 67.**
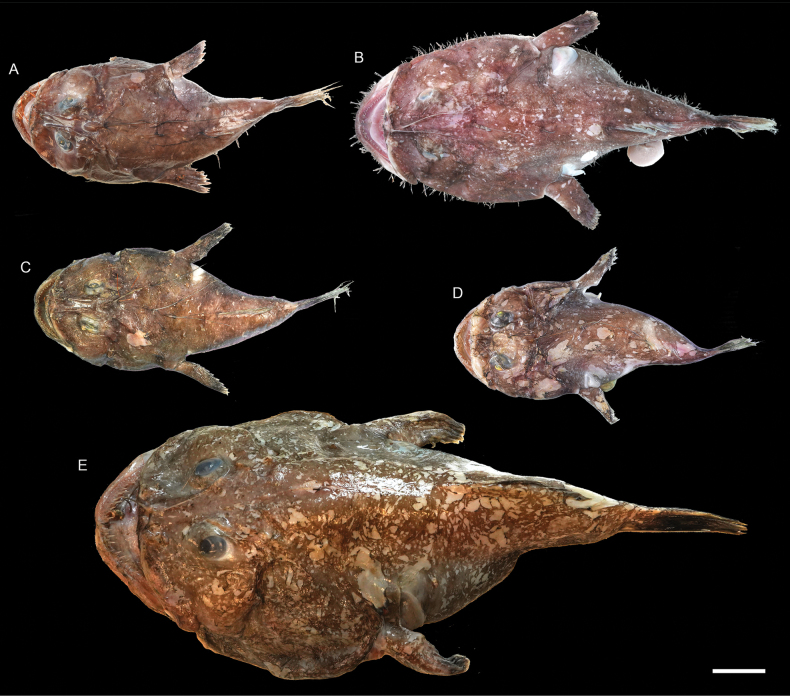
Images of fresh specimens collected around Dongsha Island **A***Lophiodesiwamotoi*, 228.40 mm SL**B***Lophiodeslugubris*, 313.96 mm SL**C***Lophiodesnaresi*, 231.87 mm SL**D***Lophiodesmutilus*, 180.04 mm SL**E***Lophiodestriradiatus*, 513.12 mm SL. Scale bar: 5 cm.

**Figure 68.**
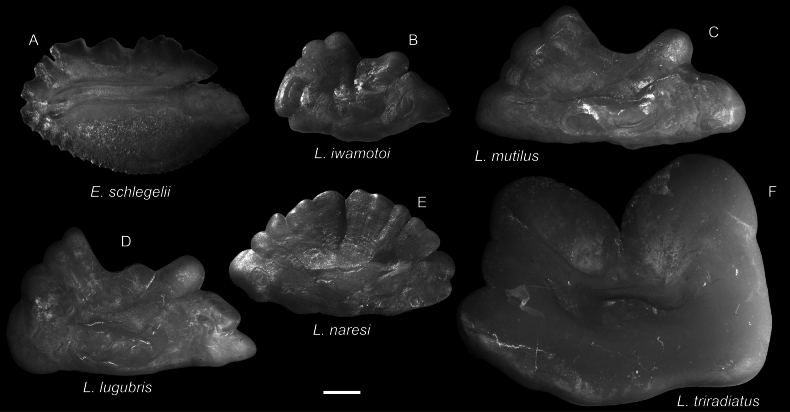
Otolith images of fresh specimens collected around Dongsha Island **A***Erythroclesschlegelii*, CHLOL 21498, 177.46 mm SL (R) **B***Lophiodesiwamotoi*, CHLOL 23849, 109.84 mm SL**C***Lophiodesmutilus*, CHLOL 23149, 250.73 mm SL**D***Lophiodeslugubris*, CHLOL 23262, 265.64 mm SL**E***Lophiodesnaresi*, CHLOL 9768, 185.84 mm SL**F***Lophiodestriradiatus*, CHLOL 20515, 437.17 mm SL. Scale bar: 1 mm.

***Chaunaxapus* Lloyd, 1909**

Figs 69D, 71B

This species is widespread in the Indo-West Pacific. Although not previously recorded from Dongsha Island, we found it very common in the area but rare around Taiwan.

**Figure 69.**
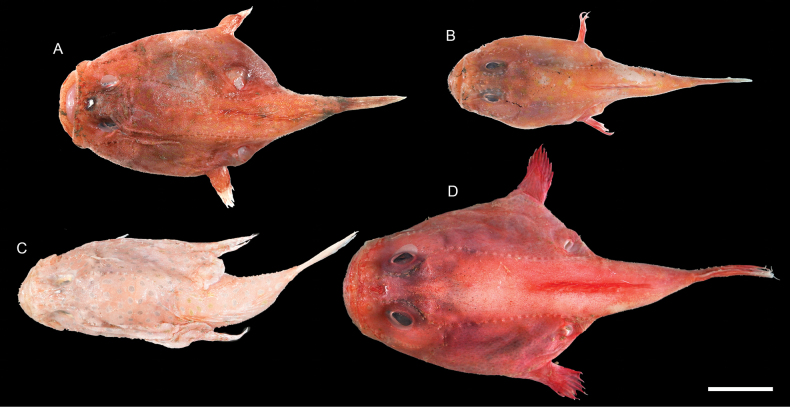
Images of fresh specimens collected around Dongsha Island **A***Chaunaxpenicillatus*, 108.89 mm SL**B***Chaunax* sp., 103.01 mm SL**C***Chaunaxbreviradius*, 131.88 mm SL**D***Chaunaxapus*, 124.73 mm SL. Scale bar: 3 cm.

***Chaunaxbreviradius* Le Danois, 1978**

Figs 69C, 71C

This species is common around Taiwan and is restricted to the South China Sea. It has never been recorded around Dongsha Island before, until the present study.

***Halieutopsisechinoderma* Ho, 2021**

Figs 70A, 71K

This species was recently described from Taiwan and the Coral Sea, and one specimen was collected around Dongsha Island, suggesting its broad distribution in the central Indo-West Pacific.

**Figure 70.**
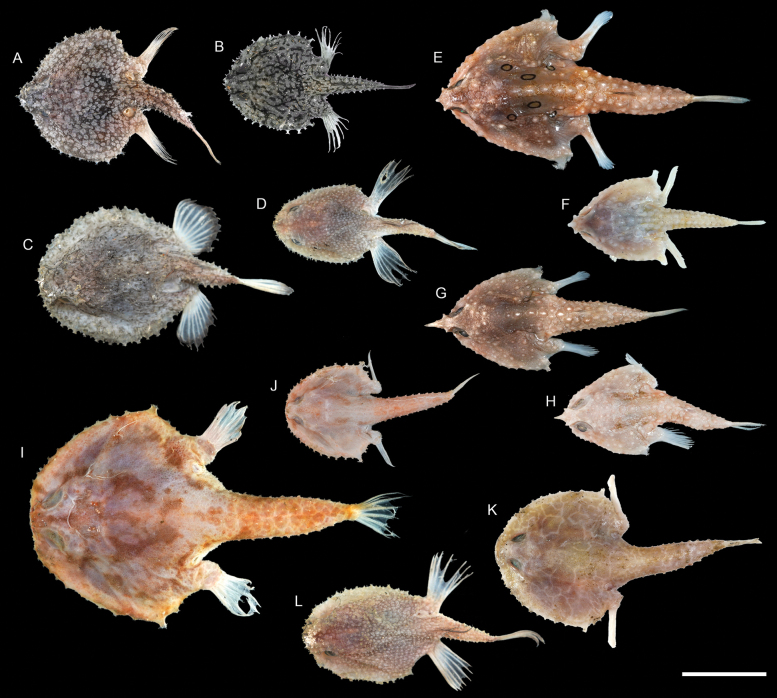
Images of fresh specimens collected around Dongsha Island **A***Halieutopsisechinoderma*, 64.26 mm SL**B***Halieutopsisnasuta*, 54.01 mm SL**C***Halieutaeacoccinea*, 65.84 mm SL**D***Halieutopsis* sp., 53.07 mm SL**E***Malthopsisannulifera*, 85.54 mm SL**F***Malthopsismitrigera*, 55.29 mm SL**G***Malthopsiskobayashii*, 75.44 mm SL**H***Malthopsistiarella*, 66.21 mm SL**I***Halicmetusruber*, 86.79 mm SL**J**Halicmetuscf.ruber, 56.29 mm SL**K***Halicmetusreticulatus*, 77.90 mm SL**L***Coelophrysmicropus*, 64.00 mm SL. Scale bar: 3 cm.

***Halieutopsisnasuta* Alcock, 1891**

Figs 70B, 71L

Although being widespread in the Indo-West Pacific, this species is very rare around Dongsha Island, known only from a few specimens. The present record confirms its appearance in the South China Sea.

**Figure 71.**
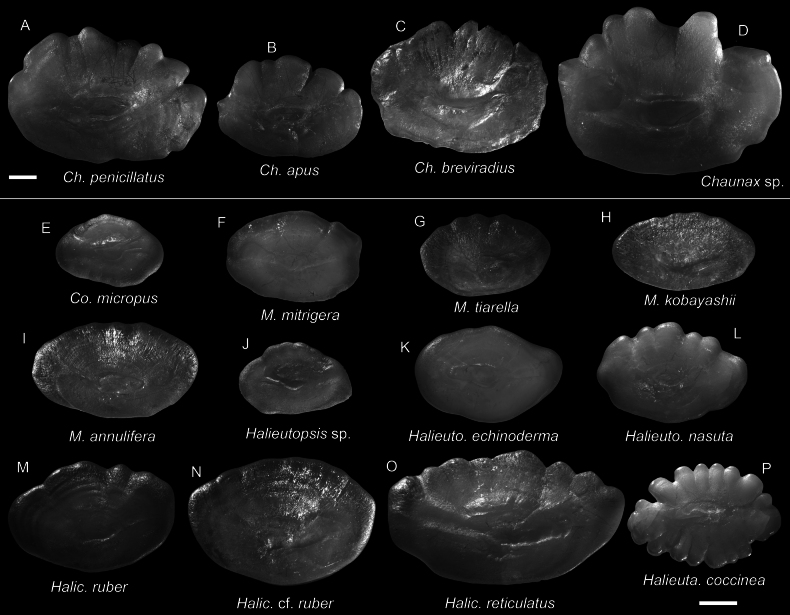
Otolith images of fresh specimens collected around Dongsha Island **A***Chaunaxpenicillatus*, CHLOL 20309, 127.92 mm SL**B***Chaunaxapus*, CHLOL 20308, 95.51 mm SL**C***Chaunaxbreviradius*, CHLOL 5399, 131.88 mm SL**D***Chaunax* sp., CHLOL 15746, 160.97 mm SL**E***Coelophrysmicropus*, CHLOL 27454, 64.00 mm SL**F***Malthopsismitrigera*, CHLOL 17434, 55.29 mm SL**G***Malthopsistiarella*, CHLOL 4394, 58.70 mm SL**H***Malthopsiskobayashii*, CHLOL 6852, 61.09 mm SL**I***Malthopsisannulifera*, CHLOL 3678, 85.54 mm SL**J***Halieutopsis* sp., CHLOL 18748, 46.37 mm SL**K***Halieutopsisechinoderma*, CHLOL 29043, 64.26 mm SL**L***Halieutopsisnasuta*, CHLOL 25666, 73.65 mm SL**M***Halicmetusruber*, CHLOL 21065, 65.15 mm SL**N**Halicmetuscf.ruber, CHLOL 24204, 64.74 mm SL**O***Halicmetusreticulatus*, CHLOL 20910, 101.23 mm SL**P***Halieutaeacoccinea*, CHLOL 27444, 65.84 mm SL. Scale bars: 1 mm.

**Figure 72.**
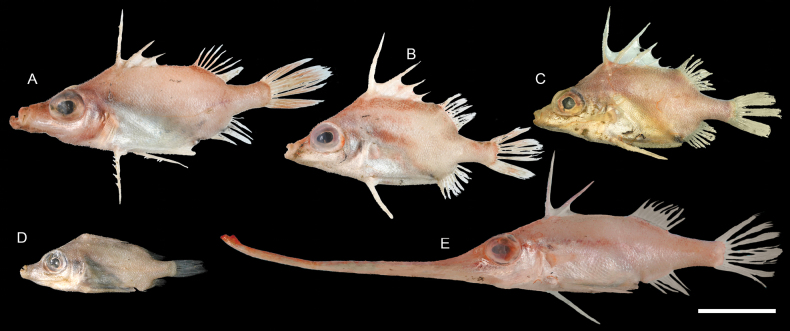
Images of fresh specimens collected around Dongsha Island **A***Tydemanianavigatoris*, 95.54 mm SL**B***Triacanthodesanomalus*, 76.52 mm SL**C***Paratriacanthodesretrospinis*, 70.23 mm SL**D***Bathyphylaxbombifrons*, 58.07 mm SL**E***Halimochirurgusalcocki*, 185.04 mm SL. Scale bar: 3 cm.

**Figure 73.**
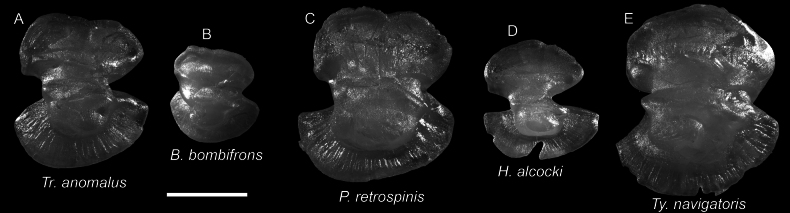
Otolith images of fresh specimens collected around Dongsha Island **A***Triacanthodesanomalus*, CHLOL 22706, 73.57 mm SL**B***Bathyphylaxbombifrons*, CHLOL 12732, 43.93 mm SL**C***Paratriacanthodesretrospinis*, CHLOL 23527, 90.30 mm SL (R) **D***Halimochirurgusalcocki*, CHLOL 22698, 143.53 mm SL (R) **E***Tydemanianavigatoris*, CHLOL 22702, 110.20 mm SL (R). Scale bar: 1 mm.

***Malthopsiskobayashii* Tanaka, 1916**

Figs 70G, 71H

This species was originally described from Japan and resurrected by Ho and Shao (2010), with additional information provided in Ho and Koeda (2019). Although it is widespread in the Indo-West Pacific and common around Taiwan, it is rare around Dongsha Island.

### ﻿Species that were not identified to specific level

***Okamejei* sp.**

Fig. 3G

This sole neonate specimen is light yellow on the dorsal side and white on the ventral side, without any blotches or patterns. This species is under investigation by SLN.

***Mobula* spp.**

Several large specimens were observed at the landing site but were dissected, making specific identification impossible. Nevertheless, this record shows the existence of *Mobula* species in the deep waters around Dongsha Island.

***Neenchelys* sp.**

Fig. 9B

This sole specimen belongs to *Neenchelys* but morphologically does not resemble any members.

***Ophichthus* spp.**

Figs 9D, 10C

These specimens belong to *Ophichthus*. More specimens without precise identification are found in the collection.

***Bathycongrus* sp.**

Figs 12E, 13C

This species has certain differences in morphology from other congeners. It is common around Dongsha Island.

**Bathyurocongercf.vicinus**

Figs 13F, 14F

The sole specimen resembles *B.vicinus*, but has a longer dorsal fin and more elongated body.

***Bathyuroconger* sp.**

Figs 13I, 14J

This species is most similar to *B.albus*, but its otoliths are distinctly different from those of the congeners.

***Argyripnus* sp.**

Figs 17F, 18D

This species represents the first record of the genus in the South China Sea. However, the specimen was partially damaged and was not retained.

***Lestidiops* sp.**

Figs 25C, 26B

One specimen with a partially damaged body was identified at a generic level. This species is the only representative of the genus from our collection.

***Magnisudis* sp.**

Figs 25J, 26I

This species is uncommon around Dongsha Island. Most of the individuals are severely damaged.

**Stemonosudiscf.siliquiventer**

Figs 25A, 26G

This species is rare around Dongsha Island but common around Taiwan (Ho et al. 2019a). The morphology of these specimens is slightly different from the Atlantic population.

***Sudis* sp.**

Figs 25H, 26J

A single specimen was collected from Dongsha Island. Although both species under *Sudis* were predicted to be circumglobal, our specimen represents the first record of the genus in the South China Sea.

***Neoscopelus* sp.**

Figs 27D, 29D

This species is currently known only around Dongsha Island, and is abundant. It differs from other congeners by the unique photophore pattern and the pinkish coloration and is recognized as undescribed.

***Bolinichthys* spp.**

Figs 27I, 29F

The specimens are easily recognizable as *Bolinichthys* by having a whitish crescent on the posterior edge of the eye. However, almost all specimens are partially damaged and lack diagnostic photophores; thus, species-level identification is impossible. They are very common around Dongsha Island.

***Ceratoscopelus* sp.**

Figs 27K, 29J

*Ceratoscopeluswarmingii* (Lütken, 1892) is the only species previously reported in the South China Sea (Randall and Lim 2000). However, almost all specimens are partially damaged, lacking the diagnostic photophores, making them difficult to identify. In addition, the relationship between *C.warmingii* and the circumglobal *C.townsendi* (Eigenmann & Eigenmann, 1889) is also unclear (Badcock and Araújo 1988; dos Santos et al. 2024). This species is common around Dongsha Island.

***Lampanyctus* spp.**

Figs 27J, 32K

This genus has a complicated taxonomic history and is especially difficult to identify. All specimens are conservatively identified at a generic level. This genus is uncommon around Dongsha Island.

***Zenion* sp.**

Figs 30H, 32C

This common species is currently known only from Dongsha Island. It is similar to *Z.japonicum*, but differs in the dorsal-fin morphology and some body morphometrics. It is recognized as undescribed. and is currently under investigation by YTL in the special issue.

**Xenolepidichthyscf.dalgleishi**

Figs 30E, 32L

These specimens are similar to *X.dalgleishi* in all aspects, but without the conspicuous black blotches on the body.

***Coelorinchus* sp.**

Figs 33F, 34D

This species is similar to *C.smithi*, but differs from the morphology of the occipital scale and body morphometrics. It is the most abundant macrourid species collected around the studied area and is possibly restricted to the South China Sea.

***Mataeocephalus* sp.**

Figs 36H, 37J

This species differs from the two sympatric species, *M.cristatus* Sazonov, Shcherbachev & Iwamoto, 2003 and *M.hyostomus* (Smith & Radcliffe, 1912), by having the underside of snout largely naked (Iwamoto et al. 2015). However, as most specimens are small and not intact, a generic-level identification is given conservatively. It is common around Dongsha Island.

**Pseudocetonuruscf.septifer**

Figs 35E, 36A

The sole specimen collected around Dongsha Island resembles the Taiwanese specimen listed in Nakayama (2020). This species may be an undescribed species.

**Ventrifossacf.longibarbata**

Figs 37G, 38E

This species differs from *V.longibarbata* by having fewer transverse scale rows, body morphometrics, and having distinct black blotch on the first dorsal fin. The status of the species is currently under investigation by SLN. It is common around Dongsha Island.

***Physiculus* sp. 1**

Figs 39B, 40B

This species resembles the *Physiculus* sp. 1 in Koeda and Ho (2019), which is uncommon around Taiwan and rare around Dongsha Island.

***Physiculus* sp. 2**

Figs 39E, 40C

This species resembles the *Physiculus* sp. 3 in Koeda and Ho (2019), which is common in southwestern Taiwan. We only collected one specimen around Dongsha Island.

***Hoplostethus* sp.**

Figs 41H, 42A

This species is similar to *H.roseus*, but differs from the latter by having the gular region covered with scales and the oral cavity pale (Su et al. 2022).

***Encheliophis* sp.**

Figs 41D, 44B

Known only from two specimens in rather poor condition around Dongsha Island. We conservatively identified the specimens to a generic level. This is the first record of this genus in the area.

***Monomitopus* sp.**

Figs 43D, 44I

Members of this genus are exceptionally similar to each other, thus making it difficult to identify. A comprehensive taxonomic revision of all the members is needed. This species is common around Dongsha Island.

**Bythitidae indet.**

Figs 45A, 46F

This specimen resembles members of the genus *Cataetys*, but it has a more slender body. Due to the limited knowledge of the taxonomy of the bythihtids in this region, we conservatively assigned this specimen to the familial level.

***Diplacanthopoma* sp.**

Figs 45C, 46E

This genus is poorly studied, and it is uncertain how many species are valid. As a result, all specimens are identified to a generic level. This species is uncommon around Dongsha Island.

***Aphanopus* sp.**

Figs 53K, 54A

The sole specimen represents the first record of the genus *Aphanopus* around Taiwan and the South China Sea. The status of the species if under investigation by YTL.

***Benthodesmus* sp.**

Figs 53M, 54B

This species is uncommon around Dongsha Island. It is similar to *B.tenuis* in morphological aspects but with a dense black body coloration and a shorter orbital length.

***Lepidotrigla* sp.**

Figs 63E, 64E

This species resembles *Lepidotrigla* sp. sensu Yamada and Yagishita (2013) in morphological aspects. It is rare around Dongsha Island.

***Chaunax* sp.**

Figs 69B, 71D

This species is morphologically distinct from other known *Chaunax* species. It is not uncommon around Taiwan but rare around Dongsha Island.

**Halicmetuscf.ruber**

Figs 70J, 71N

This species is similar to, yet distinct from *H.ruber*. This is also common around Dongsha Island.

***Halieutopsis* sp.**

Figs 70D, 71J

This species, represented by a few specimens, is recognized as undescribed.

## ﻿Discussion

The earliest record of the ichthyofauna around Dongsha Island was a report by Chen et al. (1995), which was later incorporated by Randall and Lim (2000) into a comprehensive checklist of South China Sea fishes. Despite updates by Shao et al. (2008, 2011) in their checklists for southern Taiwan and the northern South China Sea, there has until now been no concerted effort to synthesize new data from subsequent studies. Our study presents the most exhaustive inventory to date, utilizing existing literature and newly sampled fish collections conducted between 2021 and 2024. More importantly, this is the first attempt to target samples derived from commercial fisheries, which operate with much higher fishing effort, generate larger catches using bigger fishing gear, and more broadly cover deeper and remote areas compared to typical scientific research vessels. Thus, our results provide novel insights and deeper knowledge of the fish fauna around Dongsha Island, especially for the less explored western waters off the atoll.

In this study, we cataloged a total of 1087 species across 167 families, and our recent efforts alone identified 337 species from 93 families (Suppl. material 1). Thirteen species have been described from 2021 to 2024 around Dongsha Island, and 89 species have been newly recorded in this study, with 35 species still undescribed and requiring further investigation. We revised the scientific names of previously reported species in literature following Fricke et al. (2024) from the families Torpedinidae, Rajidae, Dasyatidae, Congridae, Apogonidae, Gobiidae, Pomacentridae, Exocoetidae, Belonidae, Carangidae, Syngnathidae, Nomeidae, Mullidae, Kyphosidae, Anthiadidae, Epinephelidae, Haemulidae, Plectrogeniidae, Peristediidae, Platycephalidae, and Ostraciidae. We also categorized the families of each taxon following Fricke et al. (2024). Additionally, this study provides actual images and otoliths of the fishes from Dongsha Island, offering a tool for species identification.

The updated checklist not only enriches our understanding of marine biodiversity but also serves as a critical resource for future research and conservation initiatives. Notably, based on the IUCN Red List of Threatened Species (IUCN 2012), there were two fish species recorded in Dongsha Island that were considered Critically Endangered: the Reticulated Swellshark (*Cephaloscylliumfasciatum*) and the Oceanic Whitetip Shark (*Carcharhinuslongimanus*). Nine species are listed as Endangered: *Aetobatusnarinari*, *Centrophorusgranulosus*, *Centrophorussquamosus*, *Centrophorustessellatus*, *Cheilinusundulatus*, *Eptatretustaiwanae*, *Negaprionacutidens*, *Squalusjaponicus*, and *Squatinanebulosa*; ten as Vulnerable: *Benthobatisyangi*, *Carcharhinuslimbatus*, *Chimaeraphantasma*, *Cirrhoscylliumformosanum*, *Eptatretusfernholmi*, *Istiophorusplatypterus*, *Nebriusferrugineus*, *Pateobatisfai*, *Squalusmontalbani*, and *Taeniuropsmeyeni*; and six as Near Threatened: *Deaniacalcea*, *Dipturusgigas*, *Dipturustengu*, *Eptatretusburgeri*, *Heptranchiasperlo*, and *Hydrolagusmitsukurii*. However, most species were categorized as Least Concern or Data Deficient, highlighting the need for continued research and conservation efforts to ensure sustainable fishing practices in the region.

## ﻿Additional information

### Author ORCIDs

Shing-Lai Ng https://orcid.org/0000-0001-6914-9100

Hsin-Wei Liu https://orcid.org/0009-0001-7650-0883

Dominique P. Mediodia https://orcid.org/0000-0002-7305-6588

Yen-Ting Lin https://orcid.org/0009-0002-3641-1947

Chieh-Hsuan Lee https://orcid.org/0009-0001-8492-8556

Shih-Pin Huang https://orcid.org/0000-0002-5398-5646

Siao-Man Wu https://orcid.org/0009-0004-3702-1996

Chih-Ren Tung https://orcid.org/0009-0001-7647-3627

Hsuan-Ching Ho https://orcid.org/0000-0003-1154-601X

Chien-Hsiang Lin https://orcid.org/0000-0002-9843-9729

### Data availability

All of the data that support the findings of this study are available in the main text or Supplementary Information.

## Supplementary materials

Supplementary material 1 Checklist of fishes around Dongsha Island Data type docx Explanation note **table S1.** Checklist of fishes around Dongsha Island based on the literature and our collection. Species recorded and described around Dongsha Island for the first time (in this study) are in bold font. https://binary.pensoft.net/file/1194236This dataset is made available under the Open Database License (http://opendatacommons.org/licenses/odbl/1.0/). The Open Database License (ODbL) is a license agreement intended to allow users to freely share, modify, and use this Dataset while maintaining this same freedom for others, provided that the original source and author(s) are credited.Shing-Lai Ng, Hsin-Wei Liu, Dominique P. Mediodia, Yen-Ting Lin, Chieh-Hsuan Lee, Ching-Feng Lin, Shih-Pin Huang, Siao-Man Wu, Chih-Ren Tung, Hsuan-Ching Ho, Chien-Hsiang Lin
