# Supplementary material for: ﻿An updated checklist of fishes of Dongsha Island, Taiwan, northern South China Sea
Source: Zookeys. 2024 Dec 9;1220:175–242. doi: 10.3897/zookeys.1220.131100 (PMC11650212; doi:10.3897/zookeys.1220.131100)
Supplement: Supplementary material 1 — Checklist of fishes around Dongsha Island [file zookeys-1220-175_article-131100__-s001.docx]

**Supplementary Table 1.** Checklist of fishes around Dongsha Island based on the literature and our collection. Species recorded and described around Dongsha Island for the first time (in this study) are in bold font.

| **Family** | **Species** | **Chen et al. 1995** | **Shao et al. 2008** | **Shao et al. 2011** | **Chang et al. 2012** | **Ebert et al. 2013** | **Xu et al. 2019** | **This study** |
| --- | --- | --- | --- | --- | --- | --- | --- | --- |
| Myxinidae | *Eptatretus burgeri* |  | + |  |  |  |  |  |
|  | *Eptatretus fernholmi* |  |  |  |  |  |  | 2C |
|  | *Eptatretus okinoseanus* |  | + |  |  |  |  | 2A |
|  | ***Eptatretus sheni*** |  |  |  |  |  |  | 2B |
|  | ***Eptatretus taiwanae*** |  |  |  |  |  |  | 2D |
|  | *Eptatretus yangi* |  | + |  |  |  |  |  |
|  | ***Rubicundus rubicundus*** |  |  |  |  |  |  | 2E |
| Chimaeridae | *Chimaera phantasma* |  | + |  |  |  |  | 3A |
|  | *Hydrolagus mitsukurii* |  |  |  |  |  |  | 3B |
| Parascylliidae | *Cirrhoscyllium formosanum* |  |  |  |  |  |  | 4A |
| Ginglymostomatidae | *Nebrius ferrugineus* | + | + | + |  |  |  |  |
| Alopidae | *Alopias pelagicus* |  |  |  |  |  |  |  |
|  | *Alopias supercilious* |  |  |  |  |  |  |  |
|  | *Alopias vulpinus* |  |  |  |  |  |  |  |
| Carcharhinidae | *Carcharhinus limbatus* | + | + | + |  |  |  |  |
|  | *Carcharhinus longimanus* | + | + | + |  |  |  |  |
|  | *Carcharhinus melanopterus* |  |  | + |  |  |  |  |
|  | *Negaprion acutidens* |  | + | + | + |  |  |  |
| Dichichthyidae | *Dichichthys melanobranchus* |  |  |  |  |  |  | 4B |
| Pentanchidae | *Apristurus herklotsi* |  |  |  |  |  |  | 4C |
|  | *Apristurus macrostomus* |  |  |  |  |  |  | 4D |
|  | ***Apristurus nakayai*** |  |  |  |  |  |  | 4E |
|  | *Galeus eastmani* |  |  |  |  |  |  | 4F |
|  | *Galeus nipponensis* |  |  |  |  |  |  | 4G |
|  | *Galeus sauteri* |  |  |  |  |  |  | 4H |
| Scyliorhinidae | *Cephaloscyllium fasciatum* |  | + |  |  |  |  | 4I |
| Triakidae | ***Iago garricki*** |  |  |  |  |  |  | 4J |
| Hexanchidae | *Heptranchias perlo* |  |  |  |  |  |  | 5G |
| Centrophoridae | *Centrophorus granulosus* |  |  |  |  |  |  |  |
|  | *Centrophorus squamosus* |  |  |  |  |  |  |  |
|  | ***Centrophorus tessellatus*** |  |  |  |  |  |  | 5H |
|  | *Deania calcea* |  |  |  |  |  |  |  |
| Etmopteridae | ***Etmopterus bigelowi*** |  |  |  |  |  |  | 5A |
|  | *Etmopterus brachyurus* |  |  |  |  |  |  | 5B |
|  | *Etmopterus decacuspidatus* |  |  |  |  |  |  | 5C |
|  | ***Etmopterus lii*** |  |  |  |  |  |  | 5D |
|  | *Etmopterus* cf. *molleri* |  |  |  |  |  |  | 5E |
|  | *Etmopterus lucifer* |  |  |  |  |  |  | 5F |
|  | *Etmopterus pusillus* |  | + |  |  |  |  |  |
| Somniosidae | *Zameus squamulosus* |  |  |  |  |  |  | 5K |
| Squalidae | *Squalus japonicus* |  |  |  |  |  |  | 5J |
|  | ***Squalus montalbani*** |  |  |  |  |  |  | 5I |
| Squatinidae | *Squatina nebulosa* |  |  |  |  |  |  |  |
| Pristiophoridae | *Pristiophorus japonicus* |  | + |  |  | + |  | 5L |
| Torpedinidae | *Tetronarce tokionis* |  | + |  |  |  |  | 3J |
| Anacanthobatidae | *Sinobatis borneensis* |  |  |  |  |  |  | 3C |
| Arhynchobatidae | *Notoraja tobitukai* |  |  |  |  | + |  | 3D |
| Narcinidae | *Benthobatis yangi* |  |  |  |  |  |  |  |
| Rajidae | *Dipturus gigas* |  |  |  |  |  |  | 3E |
|  | *Dipturus tengu* |  | + |  |  |  |  | 3F |
|  | *Dipturus wuhanlingi* |  |  |  |  |  |  | 3I |
|  | ***Okamejei picta*** |  |  |  |  |  |  | 3H |
|  | ***Okamejei* sp.** |  |  |  |  |  |  | 3G |
| Hexatrygonidae | *Hexatrygon bickelli* |  | + |  |  |  |  |  |
| Mobulidae | ***Mobula* spp.** |  |  |  |  |  |  |  |
| Plesiobatidae | *Plesiobatis daviesi* |  |  |  |  |  |  | 3K |
| Dasyatidae | *Taeniurops meyeni* |  | + | + |  |  |  |  |
|  | *Pateobatis fai* |  |  | + |  |  |  |  |
| Myliobatidae | *Aetobatus narinari* |  | + | + | + |  |  |  |
| Halosauridae | *Aldrovandia affinis* |  |  |  |  |  |  | 6A, 7A |
| Synaphobranchidae | *Ilyophis brunneus* |  |  | + |  |  |  |  |
|  | ***Atractodenchelys brevitrunca*** |  |  |  |  |  |  | 6I, 7I |
|  | *Dysomma anguillare* |  |  |  |  |  |  | 6C, 7E |
|  | *Dysomma dolichosomatum* |  |  |  |  |  |  | 6E, 7F |
|  | ***Dysomma polycatodon*** |  |  |  |  |  |  | 6B, 7G |
|  | *Dysommina orientalis* |  |  |  |  |  |  | 6D, 7H |
|  | *Synaphobranchus affinis* |  |  |  |  |  |  | 6H, 7D |
|  | *Synaphobranchus kaupii* |  |  |  |  |  |  | 6F, 7C |
|  | ***Synaphobranchus oligolepis*** |  |  |  |  |  |  | 6G, 7B |
| Muraenidae | *Echidna nebulosa* |  |  | + |  |  |  |  |
|  | *Echidna polyzona* |  |  | + |  |  |  |  |
|  | *Gymnomuraena zebra* |  |  | + |  |  |  |  |
|  | *Gymnothorax berndti* |  |  | + |  |  |  |  |
|  | *Gymnothorax eurostus* |  |  | + |  |  |  |  |
|  | *Gymnothorax favagineus* |  |  | + |  |  |  |  |
|  | *Gymnothorax fimbriatus* |  |  | + |  |  |  |  |
|  | *Gymnothorax flavimarginatus* |  |  | + |  |  |  |  |
|  | *Gymnothorax javanicus* |  |  | + |  |  |  |  |
|  | *Gymnothorax margaritophorus* |  |  | + |  |  |  |  |
|  | *Gymnothorax meleagris* |  |  | + |  |  |  |  |
|  | *Gymnothorax thyrsoideus* |  |  | + |  |  |  |  |
|  | *Gymnothorax undulatus* |  |  | + |  |  |  |  |
| Colocongridae | ***Coloconger maculatus*** |  |  |  |  |  |  | 8A, 10H |
|  | ***Coloconger raniceps*** |  |  |  |  |  |  | 8C, 10I |
|  | *Coloconger scholesi* |  |  |  |  |  |  | 8B, 10J |
| Ophichthidae | *Myrichthys colubrinus* |  |  | + |  |  |  |  |
|  | ***Neenchelys* sp.** |  |  |  |  |  |  | 9B |
|  | ***Ophichthus kusanagi*** |  |  |  |  |  |  | 9G |
|  | ***Ophichthus megaops*** |  |  |  |  |  |  | 9H, 10B |
|  | ***Ophichthus obtusus*** |  |  |  |  |  |  | 9C, 10E |
|  | ***Ophichthus pratasensis*** |  |  |  |  |  |  | 9E, 10A |
|  | *Ophichthus urolophus* |  |  |  |  |  |  | 9F, 10D |
|  | *Pisodonophis boro* |  |  |  |  |  |  | 9A, 10F |
|  | ***Ophichthus* spp.** |  |  |  |  |  |  | 9D, 10C |
| Muraenesocidae | *Muraenesox bagio* |  | + |  |  |  |  |  |
| Nettastomatidae | *Nettastoma parviceps* |  | + |  |  |  |  |  |
|  | *Nettastoma solitarium* |  |  |  |  |  |  | 9I, 10G |
| Congridae | ***Acromycter nezumi*** |  |  |  |  |  |  | 11F, 14A |
|  | ***Ariosoma emmae*** |  |  |  |  |  |  | 11J, 12I |
|  | *Ariosoma majus* |  | + |  |  |  |  |  |
|  | *Ariosoma meeki* |  |  |  |  |  |  | 11I, 12G |
|  | ***Bathycongrus bimaculatus*** |  |  |  |  |  |  | 13D |
|  | ***Bathycongrus bleekeri*** |  |  |  |  |  |  | 12D, 13E |
|  | *Bathycongrus retrotinctus* |  | + |  |  |  |  | 12F, 13A |
|  | *Bathycongrus wallacei* |  |  |  |  |  |  | 12C, 13B |
|  | ***Bathycongrus* sp.** |  |  |  |  |  |  | 12E, 13C |
|  | *Bathyuroconger albus* |  |  |  |  |  |  | 13H, 14H |
|  | *Bathyuroconger fowleri* |  |  |  |  |  |  | 13J, 14G |
|  | ***Bathyuroconger parvibranchialis*** |  |  |  |  |  |  | 13G, 14I |
|  | ***Bathyuroconger* cf. *vicinus*** |  |  |  |  |  |  | 13F, 14F |
|  | ***Bathyuroconger* sp.** |  |  |  |  |  |  | 13I, 14J |
|  | ***Blachea xenobranchialis*** |  |  |  |  |  |  | 11G, 14C |
|  | *Conger cinereus* | + | + | + |  |  |  |  |
|  | ***Congriscus maldivensis*** |  |  |  |  |  |  | 11B, 12B |
|  | ***Congriscus megastoma*** |  |  |  |  |  |  | 11A, 12A |
|  | *Gavialiceps taiwanensis* |  |  |  |  |  |  | 11K, 14B |
|  | *Gnathophis ginanago* |  | + |  |  |  |  |  |
|  | *Gnathophis heterognathos* |  |  | + |  |  |  | 11H, 14E |
|  | *Japonoconger sivicolus* |  | + |  |  |  |  |  |
|  | ***Macrocephenchelys brachialis*** |  |  |  |  |  |  | 11D, 14D |
|  | *Macrocephenchelys brevirostris* |  |  |  |  |  |  | 11E, 12H |
| Nemichthyidae | *Nemichthys scolopaceus* |  |  |  |  |  |  | 11C, 16A |
| Engraulidae | *Encrasicholina punctifer* |  | + |  |  |  | + |  |
| Clupeidae | *Spratelloides gracilis* |  |  | + |  |  |  |  |
| Alepocephalidae | ***Rouleina squamilatera*** |  |  |  |  |  |  | 15E, 16D |
|  | *Rouleina watasei* |  |  |  |  |  |  | 15D, 16E |
|  | *Xenodermichthys nodulosus* |  | + |  |  |  |  |  |
| Chanidae | *Chanos chanos* | + | + | + | + |  |  |  |
| Gonorynchidae | *Gonorynchus abbreviatus* |  |  | + |  |  |  |  |
| Plotosidae | *Plotosus lineatus* | + | + | + |  |  |  |  |
| Argentinidae | *Argentina kagoshima* |  | + |  |  |  |  |  |
| Microstomatidae | *Nansenia ardesiaca* |  | + |  |  |  |  | 15B, 16F |
| Gonostomatidae | ***Diplophos vicinia*** |  |  |  |  |  |  | 15C, 16C |
|  | *Sigmops elongatus* |  |  |  |  |  |  | 15A, 16B |
| Sternoptychidae | ***Argyripnus* sp.** |  |  |  |  |  |  | 17F, 18D |
|  | *Argyropelecus affinis* |  |  |  |  |  |  | 17J, 18E |
|  | *Argyropelecus aculeatus* |  | + |  |  |  |  |  |
|  | *Argyropelecus gigas* |  | + |  |  |  |  |  |
|  | ***Polyipnus matsubarai*** |  |  |  |  |  |  | 17A, 18A |
|  | ***Polyipnus spinifer*** |  |  |  |  |  |  | 17B, 18B |
|  | *Polyipnus stereope* |  |  |  |  |  |  | 17C, 18C |
|  | *Sternoptyx diaphana* |  |  |  |  |  |  | 17E, 20A |
|  | *Sternoptyx pseudobscura* |  |  |  |  |  |  | 17D |
| Phosichthyidae | ***Polymetme corythaeola*** |  |  |  |  |  |  | 17I, 18H |
|  | *Polymetme elongata* |  |  |  |  |  | + | 17H, 18F |
|  | ***Polymetme surugaensis*** |  |  |  |  |  |  | 17G, 18G |
| Stomiidae | *Astronesthes chrysophekadion* |  |  |  |  |  |  | 19C, 22E |
|  | *Astronesthes indica* |  |  |  |  |  |  | 19B, 22C |
|  | *Astronesthes indopacifica* |  |  |  |  |  |  | 19D, 22B |
|  | *Astronesthes lucifer* |  |  |  |  |  |  | 19A, 22D |
|  | *Astronesthes splendidus* |  | + |  |  |  |  |  |
|  | *Astronesthes trifibulata* |  |  |  |  |  |  | 19E, 22A |
|  | ***Borostomias elucens*** |  |  |  |  |  |  | 21A, 22G |
|  | ***Borostomias pacificus*** |  |  |  |  |  |  | 19I, 22H |
|  | *Chauliodus sloani* |  | + |  |  |  |  | 19F, 20D |
|  | *Heterophotus ophistoma* |  | + |  |  |  |  |  |
|  | *Idiacanthus fasciola* |  | + |  |  |  |  |  |
|  | *Leptostomias robustus* |  | + |  |  |  |  | 21B, 22F |
|  | *Melanostomias melanops* |  |  |  |  |  |  |  |
|  | *Photonectes albipennis* |  |  |  |  |  |  | 19J, 20B |
|  | ***Photostomias tantillux*** |  |  |  |  |  |  | 19G, 20C |
|  | *Stomias nebulosus* |  |  |  |  |  |  | 19H, 20E |
| Ateleopodidae | *Ateleopus japonicus* |  | + |  |  |  |  | 23D, 24D |
|  | *Ijimaia dofleini* |  |  |  |  |  |  | 23E, 24E |
| Synodontidae | *Saurida gracilis* | + | + | + |  |  |  |  |
|  | *Synodus binotatus* |  |  | + |  |  |  |  |
|  | *Synodus jaculum* |  |  | + |  |  |  |  |
|  | *Synodus* sp. |  |  | + |  |  |  |  |
|  | *Synodus variegatus* | + | + | + |  |  |  |  |
| Ipnopidae | *Bathytyphlops marionae* |  |  | + |  |  |  |  |
| Chlorophthalmidae | *Chlorophthalmus acutifrons* |  |  |  |  |  |  | 23A, 24A |
|  | ***Chlorophthalmus pectoralis*** |  |  |  |  |  |  | 23B, 24B |
|  | *Chlorophthalmus* sp. |  |  |  |  |  | + |  |
| Scopelarchidae | *Rosenblattichthys alatus* |  |  |  |  |  |  | 23C, 24C |
| Evermannellidae | *Evermannella indica* |  | + |  |  |  |  |  |
| Paralepididae | ***Dolichosudis fuliginosa*** |  |  |  |  |  |  | 25I, 26A |
|  | ***Lestidiops* sp.** |  |  |  |  |  |  | 25C, 26B |
|  | ***Lestidium longilucifer*** |  |  |  |  |  |  | 25D, 26E |
|  | ***Lestidium orientale*** |  |  |  |  |  |  | 25F, 26C |
|  | ***Lestidium prolixum*** |  |  |  |  |  |  | 25E, 26D |
|  | ***Lestrolepis philippina*** |  |  |  |  |  |  | 25G, 26F |
|  | ***Magnisudis* sp.** |  |  |  |  |  |  | 25J, 26I |
|  | ***Stemonosudis* cf. *siliquiventer*** |  |  |  |  |  |  | 25A, 26G |
|  | ***Stemonosudis rothschildi*** |  |  |  |  |  |  | 25B, 26H |
|  | ***Sudis* sp.** |  |  |  |  |  |  | 25H, 26J |
| Neoscopelidae | *Neoscopelus macrolepidotus* |  |  |  |  |  |  | 27C, 29A |
|  | *Neoscopelus microchir* |  |  |  |  |  | + | 27B, 29B |
|  | *Neoscopelus porosus* |  |  |  |  |  |  | 27A, 29C |
|  | ***Neoscopelus* sp.** |  |  |  |  |  |  | 27D, 29D |
| Myctophidae | *Myctophum spinosum* |  | + |  |  |  |  |  |
|  | *Benthosema fibulatum* |  |  |  |  |  |  | 27L, 29E |
|  | ***Bolinichthys* spp.** |  |  |  |  |  |  | 27I, 29F |
|  | ***Ceratoscopelus* sp.** |  |  |  |  |  |  | 27K, 29J |
|  | *Dasyscopelus asper* |  |  |  |  |  |  | 27F, 29H |
|  | *Dasyscopelus obtusirostris* |  |  |  |  |  |  | 27E, 29G |
|  | ***Dasyscopelus selenops*** |  |  |  |  |  |  | 27G, 29I |
|  | ***Diaphus adenomus*** |  |  |  |  |  |  | 28K, 32A |
|  | *Diaphus fragilis* |  |  |  |  |  |  | 28C, 29S |
|  | *Diaphus garmani* |  |  |  |  |  |  | 28F, 29K |
|  | *Diaphus lucidus* |  |  |  |  |  |  | 28B, 29P |
|  | *Diaphus luetkeni* |  |  |  |  |  |  | 28I, 29M |
|  | *Diaphus parri* |  |  |  |  |  |  | 28E, 29Q |
|  | *Diaphus perspicillatus* |  |  |  |  |  |  | 28D, 29R |
|  | *Diaphus problematicus* |  |  |  |  |  |  | 28J, 29O |
|  | *Diaphus splendidus* |  |  |  |  |  |  | 28A, 29L |
|  | *Diaphus suborbitalis* |  |  |  |  |  |  | 28G, 29N |
|  | *Diaphus watasei* |  |  |  |  |  |  | 28H, 29T |
|  | *Electrona risso* |  |  |  |  |  |  | 27H, 32G |
|  | *Lampadena luminosa* |  |  |  |  |  |  | 27M, 32B |
|  | ***Lampanyctus* spp.** |  |  |  |  |  |  | 27J, 32F |
| Trachipteridae | *Trachipterus ishikawae* |  | + |  |  |  |  |  |
|  | *Zu cristatus* |  |  |  |  |  |  | 30J |
| Polymixiidae | *Polymixia berndti* |  |  |  |  |  |  | 30C, 32D |
|  | *Polymixia longispina* |  |  |  |  |  |  | 30B, 32E |
| Parazenidae | *Cyttopsis rosea* |  |  |  |  |  |  | 30G, 32K |
|  | *Parazen pacificus* |  | + |  |  |  | + | 30A, 32O |
| Zeniontidae | *Cyttomimus affinis* |  |  |  |  |  |  | 30D, 32I |
|  | *Zenion hololepis* |  | + |  |  |  |  |  |
|  | ***Zenion japonicum*** |  |  |  |  |  |  | 30I, 32N |
|  | ***Zenion* sp.** |  |  |  |  |  |  | 30H, 32C |
| Grammicolepididae | *Grammicolepis brachiusculus* |  | + |  |  |  |  | 31A, 32J |
|  | *Xenolepidichthys dalgleishi* |  |  |  |  |  |  | 30F, 32M |
|  | ***Xenolepidichthys* cf. *dalgleishi*** |  |  |  |  |  |  | 30E, 32L |
| Zeidae | *Zenopsis nebulosa* |  |  |  |  |  |  | 31B, 32H |
|  | *Zenopsis stabilispinosa* |  | + |  |  |  |  |  |
| Bathygadidae | *Gadomus colletti* |  |  |  |  |  |  | 33A, 34A |
| Macrouridae | *Coelorinchus brevirostris* |  |  |  |  |  |  | 33D, 34E |
|  | *Coelorinchus cingulatus* |  |  |  |  |  |  | 33B, 34F |
|  | *Coelorinchus longissimus* |  |  |  |  |  | + | 33G, 34G |
|  | ***Coelorinchus macrorhynchus*** |  |  |  |  |  |  | 33E, 34H |
|  | ***Coelorinchus sheni*** |  |  |  |  |  |  | 33C, 34J |
|  | ***Coelorinchus smithi*** |  |  |  |  |  |  | 33H, 34I |
|  | ***Coelorinchus* sp.** |  |  |  |  |  |  | 33F, 34D |
|  | *Hymenocephalus lethonemus* |  |  |  |  |  |  | 35B, 36K |
|  | *Hymenocephalus longibarbis* |  |  |  |  |  |  | 35C, 36J |
|  | *Hymenocephalus striatissimus* |  |  |  |  |  |  | 35D, 36I |
|  | *Kumba gymnorhynchus* |  |  |  |  |  |  | 35F, 36G |
|  | *Kumba japonica* |  |  |  |  |  |  | 35G, 36F |
|  | *Kumba punctulata* |  |  |  |  |  |  | 35H, 36E |
|  | ***Kuronezumia bubonis*** |  |  |  |  |  |  | 34C, 37A |
|  | *Malacocephalus laevis* |  | + |  |  |  | + |  |
|  | ***Kuronezumia macronema*** |  |  |  |  |  |  | 34B, 37B |
|  | *Malacocephalus nipponensis* |  |  |  |  |  |  | 33I, 34K |
|  | ***Mataeocephalus* sp.** |  |  |  |  |  |  | 36H, 37J |
|  | *Nezumia condylura* |  |  |  |  |  |  | 36B, 37H |
|  | *Nezumia spinosa* |  |  |  |  |  |  | 36C, 37I |
|  | ***Pseudocetonurus* cf. *septifer*** |  |  |  |  |  |  | 35E, 36A |
|  | *Sphagemacrurus pumiliceps* |  |  |  |  |  |  | 35A, 36D |
|  | ***Spicomacrurus kuronumai*** |  |  |  |  |  |  | 36L, 37K |
|  | *Ventrifossa divergens* |  |  |  |  |  |  | 37F, 38C |
|  | ***Ventrifossa johnboborum*** |  |  |  |  |  |  | 37D, 38A |
|  | *Ventrifossa longibarbata* |  |  |  |  |  |  | 37C, 38B |
|  | ***Ventrifossa* cf. *longibarbata*** |  |  |  |  |  |  | 37G, 38E |
|  | *Ventrifossa sazonovi* |  |  |  |  |  |  | 37E, 38D |
| Moridae | ***Gadella jordani*** |  |  |  |  |  |  | 39D, 40A |
|  | ***Laemonema robustum*** |  |  |  |  |  |  | 39F |
|  | ***Physiculus chigodarana*** |  |  |  |  |  |  | 39A, 40D |
|  | *Physiculus japonicus* |  |  |  |  |  |  | 39C, 40E |
|  | ***Physiculus* sp. 1** |  |  |  |  |  |  | 39B, 40B |
|  | ***Physiculus* sp. 2** |  |  |  |  |  |  | 39E, 40C |
| Bregmacerotidae | *Bregmaceros lanceolatus* |  | + |  |  |  |  |  |
|  | *Bregmaceros japonicus* |  |  |  |  |  |  | 40F, 41C |
| Holocentridae | *Myripristis adusta* | + | + | + |  |  |  |  |
|  | *Myripristis berndti* |  | + | + |  |  | + |  |
|  | *Myripristis botche* | + | + | + |  |  |  |  |
|  | *Myripristis kuntee* | + | + | + |  |  |  |  |
|  | *Myripristis murdjan* | + | + | + |  |  |  |  |
|  | *Myripristis violacea* | + | + | + |  |  |  |  |
|  | *Neoniphon opercularis* |  |  | + |  |  |  |  |
|  | *Neoniphon sammara* | + | + | + |  |  |  |  |
|  | *Sargocentron caudimaculatum* | + | + | + |  |  |  |  |
|  | *Sargocentron diadema* | + | + | + |  |  |  |  |
|  | *Sargocentron ittodai* | + | + | + |  |  |  |  |
|  | *Sargocentron melanospilos* | + | + | + |  |  |  |  |
|  | *Sargocentron praslin* |  |  | + |  |  |  |  |
|  | *Sargocentron rubrum* | + | + | + |  |  |  |  |
|  | *Sargocentron spiniferum* | + | + | + |  |  |  |  |
|  | *Sargocentron spinosissimum* | + | + | + |  |  |  |  |
| Diretmidae | *Diretmoides veriginae* |  |  |  |  |  |  | 40H, 41A |
|  | *Diretmus argenteus* |  |  |  |  |  |  | 40G, 41B |
| Trachichthyidae | *Hoplostethus melanopus* |  |  |  |  |  |  | 41F, 42B |
|  | *Hoplostethus robustispinus* |  |  |  |  |  |  | 41I, 42E |
|  | ***Hoplostethus roseus*** |  |  |  |  |  |  | 41G, 42D |
|  | ***Hoplostethus* sp.** |  |  |  |  |  |  | 41H, 42A |
| Berycidae | *Beryx mollis* |  |  |  |  |  |  | 41E, 42C |
| Carapidae | ***Encheliophis* sp.** |  |  |  |  |  |  | 41D, 44B |
|  | *Pyramodon ventralis* |  | + |  |  |  |  |  |
| Ophidiidae | *Brotula multibarbata* | + |  | + |  |  |  |  |
|  | *Dicrolene tristis* |  |  |  |  |  |  | 43K, 44A |
|  | ***Glyptophidium argenteum*** |  |  |  |  |  |  | 43I, 44G |
|  | ***Glyptophidium japonicum*** |  |  |  |  |  |  | 43G, 44E |
|  | *Glyptophidium lucidum* |  |  |  |  |  |  | 43H, 44F |
|  | ***Glyptophidium oceanium*** |  |  |  |  |  |  | 43J, 44H |
|  | *Homostolus acer* |  |  |  |  |  |  | 43C, 44D |
|  | *Hoplobrotula armata* |  |  |  |  |  |  | 43B, 44C |
|  | *Lamprogrammus brunswigi* |  |  |  |  |  |  | 43N, 44M |
|  | *Luciobrotula bartschi* |  |  |  |  |  |  | 43A, 44J |
|  | ***Monomitopus* sp.** |  |  |  |  |  |  | 43D, 44I |
|  | ***Neobythites bimaculatus*** |  |  |  |  |  |  | 43E, 44O |
|  | *Neobythites longipes* |  |  |  |  |  |  | 43L, 44L |
|  | *Neobythites sivicola* |  |  |  |  |  |  | 44P |
|  | ***Neobythites unimaculatus*** |  |  |  |  |  |  | 43F, 44N |
|  | *Pycnocraspedum microlepis* |  |  |  |  |  |  | 43M, 44K |
| Bythitidae | **Bythitidae indet.** |  |  |  |  |  |  | 45A, 46F |
|  | ***Cataetyx lepidogenys*** |  |  |  |  |  |  | 45F, 46G |
|  | ***Diplacanthopoma* sp.** |  |  |  |  |  |  | 45C, 46E |
|  | ***Pseudonus squamiceps*** |  |  |  |  |  |  | 45D, 46C |
|  | ***Saccogaster horrida*** |  |  |  |  |  |  | 45E, 46A |
|  | *Saccogaster tuberculata* |  |  |  |  |  |  | 45G, 46B |
|  | ***Barathronus maculatus*** |  |  |  |  |  |  | 45B, 46D |
| Apogonidae | *Apogon coccineus* | + |  | + |  |  |  |  |
|  | *Apogon doryssa* |  |  | + |  |  |  |  |
|  | *Apogonichthyoides melas* | + |  | + |  |  |  |  |
|  | *Apogonichthyoides timorensis* |  |  | + |  |  |  |  |
|  | *Cheilodipterus artus* |  |  | + |  |  |  |  |
|  | *Cheilodipterus intermedius* |  |  | + |  |  |  |  |
|  | *Cheilodipterus macrodon* |  | + | + |  |  |  |  |
|  | *Cheilodipterus quinquelineatus* |  |  | + |  |  |  |  |
|  | *Fibramia thermalis* |  |  | + |  |  |  |  |
|  | *Foa fo* |  |  | + |  |  |  |  |
|  | *Fowleria marmorata* |  |  | + |  |  |  |  |
|  | *Fowleria variegata* |  | + | + |  |  |  |  |
|  | *Gymnapogon* sp.4 |  |  | + |  |  |  |  |
|  | *Jaydia novaeguineae* |  | + |  |  |  |  |  |
|  | *Nectamia bandanensis* |  | + |  |  |  |  |  |
|  | *Nectamia fusca* |  | + | + |  |  |  |  |
|  | *Nectamia luxuria* |  | + |  |  |  |  |  |
|  | *Nectamia savayensis* |  | + |  |  |  |  |  |
|  | *Pristiapogon exostigma* |  | + | + |  |  |  |  |
|  | *Pristiapogon fraenatus* |  |  | + |  |  |  |  |
|  | *Pristiapogon kallopterus* |  |  | + |  |  |  |  |
|  | *Pristicon trimaculatus* |  | + | + |  |  |  |  |
|  | *Pseudamia gelatinosa* |  | + | + |  |  |  |  |
|  | *Pseudamiops gracilicauda* |  | + | + |  |  |  |  |
|  | *Ostorhinchus angustatus* |  |  | + |  |  |  |  |
|  | *Ostorhinchus apogonoides* |  |  | + |  |  |  |  |
|  | *Ostorhinchus aureus* |  |  | + |  |  |  |  |
|  | *Ostorhinchus cookii* |  | + | + |  |  |  |  |
|  | *Ostorhinchus cyanosoma* |  | + | + |  |  |  |  |
|  | *Ostorhinchus compressus* |  |  | + |  |  |  |  |
|  | *Ostorhinchus doederleini* |  |  | + |  |  |  |  |
|  | *Ostorhinchus nigrofasciatus* |  |  | + |  |  |  |  |
|  | *Ostorhinchus notatus* |  | + | + |  |  |  |  |
|  | *Ostorhinchus novemfasciatus* |  |  | + |  |  |  |  |
|  | *Ostorhinchus properuptus* |  |  | + |  |  |  |  |
|  | *Ostorhinchus taeniophorus* |  |  | + |  |  |  |  |
|  | *Nectamia savayensis* |  |  | + |  |  |  |  |
|  | *Taeniamia fucata* |  |  | + |  |  |  |  |
|  | *Siphamia fuscolineata* |  |  | + |  |  |  |  |
|  | *Siphamia majimai* |  | + | + |  |  |  |  |
|  | *Siphamia versicolor* |  | + | + |  |  |  |  |
|  | *Sphaeramia nematoptera* |  | + | + |  |  |  |  |
| Eleotridae | *Eleotris fusca* |  |  | + |  |  |  |  |
| Xenisthmidae | *Xenisthmus polyzonatus* |  | + | + |  |  |  |  |
|  | *Xenisthmus* sp. 2 |  |  | + |  |  |  |  |
|  | *Xenisthmus* sp. 3 |  |  | + |  |  |  |  |
| Gobiidae | *Amblyeleotris wheeleri* |  |  | + |  |  |  |  |
|  | *Amblygobius nocturnus* |  |  | + |  |  |  |  |
|  | *Amblygobius phalaena* | + | + | + |  |  |  |  |
|  | *Amblygobius sphynx* |  |  | + |  |  |  |  |
|  | *Asterropteryx semipunctata* | + | + | + |  |  |  |  |
|  | *Bathygobius fuscus* |  |  | + |  |  |  |  |
|  | *Bathygobius niger* |  |  | + |  |  |  |  |
|  | *Bryaninops yongei* | + | + | + |  |  |  |  |
|  | *Callogobius flavobrunneus* |  |  | + |  |  |  |  |
|  | *Callogobius hastatus* |  |  | + |  |  |  |  |
|  | *Callogobius maculipinnis* |  |  | + |  |  |  |  |
|  | *Callogobius sclateri* | + | + | + |  |  |  |  |
|  | *Cryptocentrus caeruleomaculatus* |  |  | + |  |  |  |  |
|  | *Cryptocentrus nigrocellatus* |  |  | + |  |  |  |  |
|  | *Cryptocentrus strigilliceps* |  |  | + |  |  |  |  |
|  | *Cryptocentrus* sp. |  |  | + |  |  |  |  |
|  | *Ctenogobiops feroculus* | + | + | + |  |  |  |  |
|  | *Ctenogobiops mitodes* |  |  | + |  |  |  |  |
|  | *Ctenogobiops tangaroai* |  |  | + |  |  |  |  |
|  | *Eviota afelei* |  | + | + |  |  |  |  |
|  | *Eviota albolineata* |  |  | + |  |  |  |  |
|  | *Eviota bifasciata* |  |  | + |  |  |  |  |
|  | *Eviota cometa* | + | + |  |  |  |  |  |
|  | *Eviota infulata* |  |  | + |  |  |  |  |
|  | *Eviota latifasciata* | + | + | + |  |  |  |  |
|  | *Eviota melasma* | + | + | + |  |  |  |  |
|  | *Eviota prasites* | + | + | + |  |  |  |  |
|  | *Eviota nigriventris* |  |  | + |  |  |  |  |
|  | *Eviota prasites* |  |  | + |  |  |  |  |
|  | *Eviota queenslandica* | + | + | + |  |  |  |  |
|  | *Eviota sebreei* | + | + | + |  |  |  |  |
|  | *Eviota sigillata* | + | + | + |  |  |  |  |
|  | *Eviota* sp. 1 |  |  | + |  |  |  |  |
|  | *Eviota* sp. 2 |  |  | + |  |  |  |  |
|  | *Eviota* sp. 3 |  |  | + |  |  |  |  |
|  | *Eviota* sp. 4 |  |  | + |  |  |  |  |
|  | *Exyrias belissimus* |  |  | + |  |  |  |  |
|  | *Favonigobius* sp. |  |  | + |  |  |  |  |
|  | *Fusigobius duospilus* | + | + | + |  |  |  |  |
|  | *Fusigobius neophytus* | + | + | + |  |  |  |  |
|  | *Fusigobius* sp. |  |  | + |  |  |  |  |
|  | *Favonigobius* sp. |  |  | + |  |  |  |  |
|  | *Gnatholepis anjerensis* |  |  | + |  |  |  |  |
|  | *Gnatholepis cauerensis* | + | + | + |  |  |  |  |
|  | *Gnatholepis ophthalmotaenia* |  |  | + |  |  |  |  |
|  | *Gobiodon albofasciatus* |  |  | + |  |  |  |  |
|  | *Gobiodon citrinus* | + | + | + |  |  |  |  |
|  | *Gobiodon multilineatus* | + | + | + |  |  |  |  |
|  | *Gobiodon oculolineatus* | + |  | + |  |  |  |  |
|  | *Gobiodon okinawae* | + |  | + |  |  |  |  |
|  | *Istigobius decoratus* |  |  | + |  |  |  |  |
|  | *Istigobius ornatus* | + |  | + |  |  |  |  |
|  | *Istigobius rigilius* | + | + | + |  |  |  |  |
|  | *Kraemeria* sp. |  |  | + |  |  |  |  |
|  | *Lotilia graciliosa* |  |  | + |  |  |  |  |
|  | *Lubricogobius exiguus* | + |  | + |  |  |  |  |
|  | *Leucopsarion petersii* | + | + |  |  |  |  |  |
|  | *Macrodontogobius wilburi* |  |  | + |  |  |  |  |
|  | *Nemateleotris magnifica* |  |  | + |  |  |  |  |
|  | *Oplopomus oplopomus* |  |  | + |  |  |  |  |
|  | *Paragobiodon modestus* |  |  | + |  |  |  |  |
|  | *Periophthalmus modestus* | + | + | + |  |  |  |  |
|  | *Pleurosicya bilobata* | + | + | + |  |  |  |  |
|  | *Pleurosicya micheli* |  |  | + |  |  |  |  |
|  | *Priolepis cincta* | + | + | + |  |  |  |  |
|  | *Priolepis inhaca* | + | + | + |  |  |  |  |
|  | *Priolepis semidoliata* |  |  | + |  |  |  |  |
|  | *Priolepis* sp. 1 |  |  | + |  |  |  |  |
|  | *Ptereleotris evides* |  |  | + |  |  |  |  |
|  | *Ptereleotris hanae* |  |  | + |  |  |  |  |
|  | *Ptereleotris heteroptera* |  |  | + |  |  |  |  |
|  | *Ptereleotris microlepis* |  |  | + |  |  |  |  |
|  | *Trimma caesiura* |  |  | + |  |  |  |  |
|  | *Trimma emeryi* |  | + | + |  |  |  |  |
|  | *Trimma macrophthalmus* | + | + | + |  |  |  |  |
|  | *Trimma naudei* |  |  | + |  |  |  |  |
|  | *Trimma okinawae* |  |  | + |  |  |  |  |
|  | *Trimma* sp. 1 |  |  | + |  |  |  |  |
|  | *Trimma* sp. 3 |  |  | + |  |  |  |  |
|  | *Valenciennea helsdingenii* |  |  | + |  |  |  |  |
|  | *Valenciennea longipinnis* |  |  | + |  |  |  |  |
|  | *Valenciennea puellaris* |  |  | + |  |  |  |  |
|  | *Valenciennea sexguttata* |  |  | + |  |  |  |  |
|  | *Valenciennea strigata* |  |  | + |  |  |  |  |
|  | *Vanderhorstia ambanoro* |  |  | + |  |  |  |  |
|  | *Vanderhorstia* sp. |  |  | + |  |  |  |  |
| Plesiopidae | *Plesiops coeruleolineatus* |  | + | + |  |  |  |  |
|  | *Plesiops oxycephalus* |  | + | + |  |  |  |  |
| Pomacentridae | *Abudefduf notatus* |  | + | + |  |  |  |  |
|  | *Abudefduf septemfasciatus* |  |  | + |  |  |  |  |
|  | *Abudefduf sexfasciatus* |  | + | + | + |  |  |  |
|  | *Abudefduf sordidus* | + | + | + |  |  |  |  |
|  | *Abudefduf vaigiensis* |  | + | + | + |  |  |  |
|  | *Amblyglyphidodon aureus* | + | + | + |  |  |  |  |
|  | *Amblyglyphidodon curacao* | + | + | + |  |  |  |  |
|  | *Amblyglyphidodon ternatensis* | + | + | + |  |  |  |  |
|  | *Amphiprion clarkii* | + | + | + |  |  |  |  |
|  | *Amphiprion frenatus* | + | + | + |  |  |  |  |
|  | *Amphiprion perideraion* | + | + | + |  |  |  |  |
|  | *Cheiloprion labiatus* |  |  | + |  |  |  |  |
|  | *Chromis atripectoralis* | + | + | + |  |  |  |  |
|  | *Chromis atripes* | + | + | + |  |  |  |  |
|  | *Chromis chrysura* | + | + | + |  |  |  |  |
|  | *Chromis margaritifer* | + | + | + |  |  |  |  |
|  | *Chromis ovatiformis* |  |  | + |  |  |  |  |
|  | *Chromis ternatensis* | + | + | + |  |  |  |  |
|  | *Chromis viridis* | + | + | + |  |  |  |  |
|  | *Chromis weberi* |  |  | + |  |  |  |  |
|  | *Chromis xanthura* |  |  | + |  |  |  |  |
|  | *Chrysiptera biocellata* |  |  | + |  |  |  |  |
|  | *Chrysiptera brownriggii* |  |  | + |  |  |  |  |
|  | *Chrysiptera chrysocephala* |  |  | + |  |  |  |  |
|  | *Chrysiptera flavifrons* |  | + |  |  |  |  |  |
|  | *Chrysiptera kuiteri* |  |  | + |  |  |  |  |
|  | *Chrysiptera starcki* |  |  | + |  |  |  |  |
|  | *Chrysiptera unimaculata* | + | + | + | + |  |  |  |
|  | *Dascyllus aruanus* | + | + | + |  |  |  |  |
|  | *Dascyllus melanurus* |  | + |  |  |  |  |  |
|  | *Dascyllus reticulatus* | + | + | + |  |  |  |  |
|  | *Dascyllus trimaculatus* | + | + | + |  |  |  |  |
|  | *Dischistodus prosopotaenia* |  | + | + | + |  |  |  |
|  | *Hemiglyphidodon plagiometopon* | + | + | + |  |  |  |  |
|  | *Neoglyphidodon melas* |  |  | + |  |  |  |  |
|  | *Neoglyphidodon nigroris* | + | + | + |  |  |  |  |
|  | *Plectroglyphidodon dickii* | + | + | + |  |  |  |  |
|  | *Plectroglyphidodon lacrymatus* | + | + | + |  |  |  |  |
|  | *Plectroglyphidodon fasciolatus* |  |  | + |  |  |  |  |
|  | *Plectroglyphidodon leucozona* |  |  | + |  |  |  |  |
|  | *Pomacentrus adelus* |  |  | + |  |  |  |  |
|  | *Pomacentrus amboinensis* | + | + | + |  |  |  |  |
|  | *Pomacentrus bankanensis* | + | + | + |  |  |  |  |
|  | *Pomacentrus brachialis* |  |  | + |  |  |  |  |
|  | *Pomacentrus chrysurus* |  |  | + |  |  |  |  |
|  | *Pomacentrus coelestis* | + | + | + |  |  |  |  |
|  | *Pomacentrus grammorhynchus* |  |  | + | + |  |  |  |
|  | *Pomacentrus lepidogenys* | + | + | + |  |  |  |  |
|  | *Pomacentrus moluccensis* | + | + | + |  |  |  |  |
|  | *Pomacentrus nagasakiensis* |  |  | + |  |  |  |  |
|  | *Pomacentrus pavo* |  |  | + | + |  |  |  |
|  | *Pomacentrus philippinus* | + | + | + |  |  |  |  |
|  | *Pomacentrus vaiuli* |  |  | + |  |  |  |  |
|  | *Pycnochromis vanderbilti* |  |  | + |  |  |  |  |
|  | *Stegastes lacrymatus* | + | + | + |  |  |  |  |
|  | *Stegastes lividus* | + | + | + |  |  |  |  |
|  | *Stegastes nigricans* | + | + | + |  |  |  |  |
| Pseudochromidae | *Pseudochromis cyanotaenia* |  |  | + |  |  |  |  |
|  | *Pseudochromis fuscus* | + | + | + | + |  |  |  |
|  | *Pseudoplesiops* sp. |  |  | + |  |  |  |  |
|  | *Pseudochromis* sp. |  |  | + |  |  |  |  |
| Mugilidae | *Ellochelon vaigiensis* |  |  | + |  |  |  |  |
|  | *Moolgarda seheli* |  |  | + |  |  |  |  |
|  | *Neomyxus leuciscus* |  |  | + |  |  |  |  |
|  | *Planiliza macrolepis* |  |  |  | + |  |  |  |
| Tripterygiidae | *Enneapterygius philippinus* |  |  | + |  |  |  |  |
|  | *Helcogramma striata* |  | + |  |  |  |  |  |
| Blenniidae | *Aspidontus dussumieri* |  |  | + |  |  |  |  |
|  | *Aspidontus taeniatus* |  |  | + |  |  |  |  |
|  | *Atrosalarias fuscus holomelas* | + | + | + |  |  |  |  |
|  | *Cirripectes castaneus* |  |  | + |  |  |  |  |
|  | *Cirripectes variolosus* |  |  | + |  |  |  |  |
|  | *Crossosalarias macrospilus* |  |  | + |  |  |  |  |
|  | *Ecsenius bathi* |  | + |  |  |  |  |  |
|  | *Ecsenius bicolor* | + | + | + |  |  |  |  |
|  | *Ecsenius lineatus* | + | + | + |  |  |  |  |
|  | *Ecsenius namiyei* | + | + | + |  |  |  |  |
|  | *Exallias brevis* | + | + | + |  |  |  |  |
|  | *Meiacanthus atrodorsalis* |  |  | + |  |  |  |  |
|  | *Meiacanthus grammistes* | + | + | + |  |  |  |  |
|  | *Petroscirtes breviceps* |  |  | + |  |  |  |  |
|  | *Petroscirtes mitratus* | + | + | + |  |  |  |  |
|  | *Plagiotremus rhinorhynchos* |  |  | + |  |  |  |  |
|  | *Plagiotremus tapeinosoma* | + | + | + |  |  |  |  |
|  | *Salarias fasciatus* | + | + | + |  |  |  |  |
|  | *Salarias guttatus* | + | + | + |  |  |  |  |
|  | *Xiphasia setifer* |  |  |  |  |  |  | 47E, 20F |
| Exocoetidae | *Cheilopogon pinnatibarbatus* |  | + | + |  |  |  |  |
|  | *Cheilopogon cyanopterus* |  |  |  |  |  | + |  |
|  | *Cheilopogon nigricans* |  |  |  |  |  | + |  |
|  | *Cheilopogon spilonotopterus* |  |  |  |  |  | + |  |
|  | *Exocoetus monocirrhus* |  |  |  |  |  | + | 47A, 48A |
|  | *Hirundichthys oxycephalus* |  |  |  |  |  |  | 47B, 48D |
| Belonidae | *Tylosurus acus* |  |  | + |  |  |  |  |
|  | *Tylosurus crocodilus* |  | + | + |  |  |  |  |
| Coryphaenidae | *Coryphaena equiselis* |  |  |  |  |  | + |  |
|  | *Coryphaena hippurus* | + |  | + |  |  | + |  |
| Echeneidae | *Echeneis naucrates* | + | + | + |  |  |  |  |
|  | *Phtheirichthys lineatus* |  |  |  |  |  | + |  |
| Carangidae | *Atropus hedlandensis* | + | + | + |  |  |  |  |
|  | *Craterognathus plagiotaenia* |  |  | + |  |  |  |  |
|  | *Ferdauia ferdau* |  |  | + |  |  |  |  |
|  | *Ferdauia orthogrammus* | + | + | + | + |  |  |  |
|  | *Flavocaranx bajad* |  |  | + |  |  |  |  |
|  | *Caranx ignobilis* | + | + | + |  |  |  |  |
|  | *Caranx lugubris* |  |  | + |  |  |  |  |
|  | *Caranx melampygus* | + | + | + |  |  |  |  |
|  | *Caranx sexfasciatus* | + | + | + |  |  | + |  |
|  | *Decapterus maruadsi* |  |  |  |  |  | + |  |
|  | *Decapterus macarellus* |  |  |  |  |  | + | 47F, 48B |
|  | *Decapterus macrosoma* |  |  |  |  |  | + | 47C, 48C |
|  | *Decapterus tabl* |  |  |  |  |  |  | 47D, 48F |
|  | *Elagatis bipinnulata* |  |  |  |  |  | + |  |
|  | *Gnathanodon speciosus* |  |  | + |  |  |  |  |
|  | *Kaiwarinus equula* | + |  | + |  |  |  |  |
|  | *Naucrates ductor* |  |  | + |  |  |  |  |
|  | *Scomberoides lysan* |  |  | + |  |  |  |  |
|  | *Selar crumenophthalmus* |  |  |  |  |  | + |  |
|  | *Trachinotus baillonii* | + | + | + |  |  |  |  |
|  | *Trachinotus blochii* |  |  | + |  |  |  |  |
|  | *Trachurus japonicus* |  |  |  |  |  |  | 47G, 48E |
|  | *Turrum fulvoguttatum* |  |  | + |  |  |  |  |
|  | *Uraspis secunda* |  |  |  |  |  | + |  |
| Sphyraenidae | *Sphyraena barracuda* | + |  | + | + |  |  |  |
|  | *Sphyraena flavicauda* |  |  | + |  |  |  |  |
| Istiophoridae | *Istiophorus platypterus* |  |  |  |  |  | + |  |
| Citharidae | *Lepidoblepharon ophthalmolepis* |  | + |  |  |  |  | 49C, 51A |
|  | *Lepidoblepharon* sp. |  |  |  |  |  | + |  |
| Bothidae | *Asterorhombus cocosensis* |  |  | + |  |  |  |  |
|  | *Bothus pantherinus* | + | + |  |  |  |  |  |
|  | *Chascanopsetta lugubris* |  | + |  |  |  |  | 50A, 51C |
|  | *Chascanopsetta prognatha* |  |  |  |  |  |  | 50B, 51D |
| Poecilopsettidae | *Poecilopsetta plinthus* |  |  |  |  |  |  | 49B, 51B |
|  | *Poecilopsetta colorata* |  |  |  |  |  | + |  |
| Cynoglossidae | *Symphurus orientalis* |  |  |  |  |  |  | 49A, 51E |
| Soleidae | *Soleichthys heterorhinos* |  |  | + |  |  |  |  |
| Syngnathidae | *Corythoichthys flavofasciatus* | + | + | + |  |  |  |  |
|  | *Corythoichthys haematopterus* |  |  | + |  |  |  |  |
|  | *Corythoichthys isigakius* |  |  | + |  |  |  |  |
|  | *Corythoichthys schultzi* |  |  | + |  |  |  |  |
|  | *Dunckerocampus dactyliophorus* |  |  | + |  |  |  |  |
|  | *Doryrhamphus melanopleura* |  |  | + |  |  |  |  |
|  | *Hippichthys spicifer* |  |  | + |  |  |  |  |
|  | *Halicampus spinirostris* |  |  | + |  |  |  |  |
|  | *Syngnathoides biaculeatus* |  |  | + |  |  |  |  |
| Aulostomidae | *Aulostomus chinensis* |  | + | + |  |  |  |  |
| Fistulariidae | *Fistularia petimba* | + | + |  |  |  |  |  |
|  | *Fistularia commersonii* |  |  | + |  |  |  |  |
| Centriscidae | *Aeoliscus strigatus* |  |  | + |  |  |  |  |
| Callionymidae | *Diplogrammus xenicus* |  |  | + |  |  |  |  |
|  | *Synchiropus ocellatus* |  | + |  |  |  |  |  |
|  | *Synchiropus splendidus* |  |  | + |  |  |  |  |
| Scombrolabracidae | *Scombrolabrax heterolepis* |  |  |  |  |  |  | 52F, 53A |
| Gempylidae | *Gempylus serpens* |  |  |  |  |  | + | 52H, 53B |
|  | *Nealotus tripes* |  |  |  |  |  |  | 52B, 53C |
|  | *Neoepinnula orientalis* |  |  |  |  |  |  | 52G, 53F |
|  | *Nesiarchus nasutus* |  |  |  |  |  |  | 52A, 53D |
|  | *Promethichthys prometheus* |  |  |  |  |  |  | 52E, 53E |
|  | *Rexea bengalensis* |  |  |  |  |  |  | 52C, 53G |
|  | *Rexea prometheoides* |  |  |  |  |  |  | 52D, 53H |
|  | *Ruvettus pretiosus* |  |  |  |  |  | + | 52I, 53I |
|  | *Thyrsitoides marleyi* |  |  |  |  |  |  | 52J, 53J |
| Trichiuridae | ***Aphanopus* sp.** |  |  |  |  |  |  | 53K, 54A |
|  | *Benthodesmus tenuis* |  | + |  |  |  |  | 53L, 54C |
|  | ***Benthodesmus* sp.** |  |  |  |  |  |  | 53M, 54B |
|  | *Trichiurus lepturus* |  |  |  |  |  | + |  |
|  | *Trichiurus japonicus* |  |  |  |  |  | + |  |
| Scombridae | *Acanthocybium solandri* |  |  | + |  |  |  |  |
|  | *Auxis rochei* |  |  |  |  |  | + |  |
|  | *Auxis thazard* |  |  |  |  |  | + |  |
|  | *Euthynnus affinis* |  |  |  |  |  | + |  |
|  | *Katsuwonus pelamis* |  |  |  |  |  | + |  |
|  | *Sarda orientalis* | + |  | + |  |  |  |  |
|  | *Scomberomorus commerson* | + |  | + |  |  |  |  |
|  | *Scomber japonicus* |  |  |  |  |  | + |  |
|  | *Thunnus albacares* |  |  |  |  |  | + |  |
| Amarsipidae | ***Amarsipus carlsbergi*** |  |  |  |  |  |  | 55E, 56B |
| Centrolophidae | *Psenopsis anomala* |  |  |  |  |  |  | 55J, 56A |
| Nomeidae | *Cubiceps baxteri* |  | + |  |  |  |  | 55I, 56E |
|  | ***Cubiceps pauciradiatus*** |  |  |  |  |  | + | 55D, 56D |
|  | ***Cubiceps whiteleggii*** |  |  |  |  |  | + | 55C, 56C |
|  | *Psenes arafurensis* |  |  |  |  |  |  | 55H, 56F |
|  | *Psenes cyanophrys* |  |  |  |  |  |  | 55G, 56H |
|  | *Psenes maculatus* |  | + |  |  |  |  |  |
|  | *Psenes pellucidus* |  |  |  |  |  |  | 55F, 56G |
| Champsodontidae | *Champsodon guentheri* |  | + |  |  |  |  |  |
|  | ***Champsodon longipinnis*** |  |  |  |  |  |  | 55B, 56I |
|  | *Champsodon snyderi* |  |  |  |  |  |  | 55A, 56J |
| Creediidae | *Limnichthys nitidus* |  |  | + |  |  |  |  |
| Pinguipedidae | *Parapercis clathrata* |  |  | + |  |  |  |  |
|  | *Parapercis cylindrica* | + | + | + |  |  |  |  |
|  | *Parapercis millepunctata* | + | + | + |  |  |  |  |
|  | *Parapercis multiplicata* |  |  | + |  |  |  |  |
|  | *Parapercis pacifica* | + | + |  |  |  |  |  |
|  | *Parapercis schauinslandii* |  |  | + |  |  |  |  |
|  | *Parapercis snyderi* | + | + | + |  |  |  |  |
|  | *Parapercis tetracantha* |  |  | + |  |  |  |  |
| Uranoscopidae | *Xenocephalus elongatus* |  |  |  |  |  |  | 57G |
| Labridae | *Anampses caeruleopunctatus* | + | + | + |  |  |  |  |
|  | *Anampses geographicus* | + | + | + |  |  |  |  |
|  | *Anampses melanurus* | + | + | + |  |  |  |  |
|  | *Anampses meleagrides* | + | + | + |  |  |  |  |
|  | *Anampses twistii* | + | + | + |  |  |  |  |
|  | *Bodianus axillaris* |  |  | + |  |  |  |  |
|  | *Bodianus bilunulatus* | + | + | + |  |  |  |  |
|  | *Bodianus bimaculatus* |  |  | + |  |  |  |  |
|  | *Bodianus dictynna* |  |  | + |  |  |  |  |
|  | *Bodianus mesothorax* | + | + | + |  |  |  |  |
|  | *Bodianus oxycephalus* |  |  | + |  |  |  |  |
|  | *Bodianus perditio* |  |  | + |  |  |  |  |
|  | *Calotomus carolinus* |  |  | + |  |  |  |  |
|  | *Calotomus spinidens* |  |  | + |  |  |  |  |
|  | *Calotomus japonicus* | + |  |  |  |  |  |  |
|  | *Cetoscarus bicolor* | + | + | + |  |  |  |  |
|  | *Cheilinus chlorourus* | + | + | + |  |  |  |  |
|  | *Cheilinus fasciatus* | + | + | + |  |  |  |  |
|  | *Cheilinus oxycephalus* |  |  | + |  |  |  |  |
|  | *Cheilinus trilobatus* | + | + | + |  |  |  |  |
|  | *Cheilinus undulatus* | + | + | + |  |  |  |  |
|  | *Cheilio inermis* | + | + | + |  |  |  |  |
|  | *Choerodon anchorago* | + | + | + |  |  |  |  |
|  | *Choerodon jordani* |  |  | + |  |  |  |  |
|  | *Choerodon schoenleinii* |  |  | + |  |  |  |  |
|  | *Chlorurus bowersi* |  |  | + |  |  |  |  |
|  | *Chlorurus microrhinos* | + | + | + |  |  |  |  |
|  | *Chlorurus sordidus* |  |  | + |  |  |  |  |
|  | *Chlorurus spilurus* | + | + | + |  |  |  |  |
|  | *Cirrhilabrus cyanopleura* | + | + | + |  |  |  |  |
|  | *Cirrhilabrus exquisitus* |  |  | + |  |  |  |  |
|  | *Cirrhilabrus lunatus* |  |  | + |  |  |  |  |
|  | *Cirrhilabrus rubrimarginatus* |  |  | + |  |  |  |  |
|  | *Cirrhilabrus temminckii* |  |  | + |  |  |  |  |
|  | *Coris aygula* |  |  | + |  |  |  |  |
|  | *Coris dorsomacula* |  |  | + |  |  |  |  |
|  | *Coris gaimard* | + | + | + |  |  |  |  |
|  | *Cymolutes torquatus* | + | + | + |  |  |  |  |
|  | *Epibulus insidiator* | + | + | + |  |  |  |  |
|  | *Gomphosus varius* | + | + | + |  |  |  |  |
|  | *Halichoeres argus* |  |  | + |  |  |  |  |
|  | *Halichoeres biocellatus* | + | + | + |  |  |  |  |
|  | *Halichoeres chrysus* |  |  | + |  |  |  |  |
|  | *Halichoeres hartzfeldii* |  |  | + |  |  |  |  |
|  | *Halichoeres hortulanus* | + | + | + |  |  |  |  |
|  | *Halichoeres margaritaceus* | + | + | + |  |  |  |  |
|  | *Halichoeres marginatus* | + | + | + |  |  |  |  |
|  | *Halichoeres miniatus* |  |  | + |  |  |  |  |
|  | *Halichoeres nebulosus* |  |  | + |  |  |  |  |
|  | *Halichoeres orientalis* |  | + | + |  |  |  |  |
|  | *Halichoeres scapularis* | + | + | + |  |  |  |  |
|  | *Halichoeres trimaculatus* | + | + | + |  |  |  |  |
|  | *Hemigymnus fasciatus* | + | + | + |  |  |  |  |
|  | *Hemigymnus melapterus* | + | + | + |  |  |  |  |
|  | *Hipposcarus longiceps* | + | + | + |  |  |  |  |
|  | *Hologymnosus annulatus* |  |  | + |  |  |  |  |
|  | *Hologymnosus doliatus* | + | + | + |  |  |  |  |
|  | *Hologymnosus rhodonotus* |  |  | + |  |  |  |  |
|  | *Iniistius dea* | + | + | + |  |  |  |  |
|  | *Iniistius pavo* | + | + | + |  |  |  |  |
|  | *Labrichthys unilineatus* | + | + | + |  |  |  |  |
|  | *Labroides bicolor* | + | + | + |  |  |  |  |
|  | *Labroides dimidiatus* | + | + | + |  |  |  |  |
|  | *Labropsis xanthonota* |  |  | + |  |  |  |  |
|  | *Leptoscarus vaigiensis* |  | + | + | + |  |  |  |
|  | *Macropharyngodon meleagris* | + | + | + |  |  |  |  |
|  | *Macropharyngodon negrosensis* |  |  | + |  |  |  |  |
|  | *Novaculichthys taeniourus* | + | + | + |  |  |  |  |
|  | *Oxycheilinus bimaculatus* | + | + | + |  |  |  |  |
|  | *Oxycheilinus celebicus* | + | + | + |  |  |  |  |
|  | *Oxycheilinus digramma* | + | + | + |  |  |  |  |
|  | *Oxycheilinus unifasciatus* | + | + | + |  |  |  |  |
|  | *Paracheilinus carpenteri* |  |  | + |  |  |  |  |
|  | *Pseudocheilinus evanidus* | + | + | + |  |  |  |  |
|  | *Pseudocheilinus hexataenia* | + | + | + |  |  |  |  |
|  | *Pseudocheilinus octotaenia* |  |  | + |  |  |  |  |
|  | *Pseudocoris yamashiroi* |  |  | + |  |  |  |  |
|  | *Pseudodax moluccanus* |  |  | + |  |  |  |  |
|  | *Pseudojuloides cerasinus* |  |  | + |  |  |  |  |
|  | *Pteragogus aurigarius* | + | + | + |  |  |  |  |
|  | *Scarus chameleon* |  |  | + |  |  |  |  |
|  | *Scarus dimidiatus* | + | + | + |  |  |  |  |
|  | *Scarus forsteni* | + | + | + |  |  |  |  |
|  | *Scarus frenatus* | + | + | + |  |  |  |  |
|  | *Scarus fuscocaudalis* |  |  | + |  |  |  |  |
|  | *Scarus ghobban* | + | + | + |  |  |  |  |
|  | *Scarus globiceps* |  |  | + |  |  |  |  |
|  | *Scarus hypselopterus* | + | + | + |  |  |  |  |
|  | *Scarus niger* | + | + | + |  |  |  |  |
|  | *Scarus oviceps* | + | + | + |  |  |  |  |
|  | *Scarus psittacus* | + | + | + |  |  |  |  |
|  | *Scarus rivulatus* | + | + | + |  |  |  |  |
|  | *Scarus rubroviolaceus* | + | + | + |  |  |  |  |
|  | *Scarus schlegeli* | + | + | + |  |  |  |  |
|  | *Scarus spinus* | + | + | + |  |  |  |  |
|  | *Stethojulis bandanensis* | + | + | + |  |  |  |  |
|  | *Stethojulis strigiventer* | + |  | + | + |  |  |  |
|  | *Stethojulis terina* | + | + | + |  |  |  |  |
|  | *Stethojulis trilineata* |  |  | + |  |  |  |  |
|  | *Thalassoma amblycephalum* | + | + | + |  |  |  |  |
|  | *Thalassoma hardwicke* | + | + | + |  |  |  |  |
|  | *Thalassoma jansenii* | + | + | + |  |  |  |  |
|  | *Thalassoma lunare* | + | + | + |  |  |  |  |
|  | *Thalassoma lutescens* | + | + | + |  |  |  |  |
|  | *Thalassoma purpureum* | + | + | + |  |  |  |  |
|  | *Thalassoma quinquevittatum* | + | + | + |  |  |  |  |
|  | *Wetmorella nigropinnata* |  |  | + |  |  |  |  |
| Latidae | *Psammoperca waigiensis* |  |  | + |  |  |  |  |
| Gerreidae | *Gerres oyena* | + |  | + | + |  |  |  |
| Howellidae | *Bathysphyraenops simplex* |  |  |  |  |  |  | 56K, 57C |
| Acropomatidae | *Acropoma hanedai* |  | + |  |  |  |  |  |
| Malakichthyidae | ***Malakichthys elegans*** |  |  |  |  |  |  | 57D, 58H |
|  | *Malakichthys* sp. |  |  |  |  |  | + |  |
| Ostracoberycidae | *Ostracoberyx dorygenys* |  | + |  |  |  |  | 57A, 58G |
| Synagropidae | ***Parascombrops serratospinosus*** |  |  |  |  |  |  | 57B, 58A |
|  | *Synagrops japonicus* |  |  |  |  |  | + | 57E, 58B |
|  | ***Synagrops atrumoris*** |  |  |  |  |  |  | 57F, 58C |
| Epigonidae | ***Epigonus denticulatus*** |  |  |  |  |  |  | 58F, 59L |
|  | ***Epigonus megalops*** |  |  |  |  |  |  | 58E, 59K |
|  | ***Epigonus pectinifer*** |  |  |  |  |  |  | 58D, 59J |
| Mullidae | *Mulloidichthys flavolineatus* | + | + | + |  |  |  |  |
|  | *Mulloidichthys vanicolensis* | + | + | + |  |  |  |  |
|  | *Parupeneus barberinoides* | + | + | + |  |  |  |  |
|  | *Parupeneus barberinus* | + | + | + |  |  |  |  |
|  | *Parupeneus chrysopleuron* | + | + | + |  |  |  |  |
|  | *Parupeneus ciliatus* | + | + | + |  |  | + |  |
|  | *Parupeneus crassilabris* |  |  | + |  |  |  |  |
|  | *Parupeneus cyclostomus* | + | + | + |  |  |  |  |
|  | *Parupeneus heptacantha* |  |  | + |  |  |  |  |
|  | *Parupeneus indicus* | + | + | + |  |  |  |  |
|  | *Parupeneus multifasciatus* | + | + | + |  |  |  |  |
|  | *Parupeneus pleurostigma* | + | + | + |  |  |  |  |
|  | *Parupeneus trifasciatus* | + | + |  |  |  |  |  |
|  | *Parupeneus spilurus* |  |  | + |  |  |  |  |
|  | *Upeneus tragula* |  |  | + |  |  |  |  |
| Pempheridae | *Pempheris oualensis* | + | + | + |  |  |  |  |
|  | *Parapriacanthus ransonneti* |  |  | + |  |  |  |  |
| Bathyclupeidae | ***Neobathyclupea gracilis*** |  |  |  |  |  |  | 58I, 59B |
|  | ***Neobathyclupea japanotaiwana*** |  |  |  |  |  |  | 58J, 59C |
|  | ***Neobathyclupea malayana*** |  |  |  |  |  |  | 58K, 59A |
| Kyphosidae | *Kyphosus bigibbus* | + | + | + |  |  |  |  |
|  | *Kyphosus cinerascens* | + | + | + |  |  |  |  |
|  | *Kyphosus sectatrix* |  |  | + |  |  |  |  |
|  | *Kyphosus vaigiensis* | + | + | + |  |  |  |  |
| Terapontidae | *Terapon jarbua* | + | + | + | + |  |  |  |
| Pentacerotidae | *Pentaceros japonicus* |  |  |  |  |  |  | 59D, 61A |
| Anthiadidae | *Plectranthias nanus* |  |  | + |  |  |  |  |
|  | *Mirolabrichthys pascalus* |  |  | + |  |  |  |  |
|  | *Nemanthias bicolor* |  |  | + |  |  |  |  |
|  | *Pseudanthias cooperi* |  |  | + |  |  |  |  |
|  | *Pseudanthias pleurotaenia* |  |  | + |  |  |  |  |
|  | *Pseudanthias squamipinnis* |  |  | + |  |  |  |  |
|  | *Pseudogramma polyacanthus* |  |  | + |  |  |  |  |
| Epinephelidae | *Aethaloperca rogaa* |  |  | + |  |  |  |  |
|  | *Cephalopholis argus* |  |  | + |  |  |  |  |
|  | *Cephalopholis miniata* |  |  | + |  |  |  |  |
|  | *Cephalopholis sexmaculata* |  |  | + |  |  |  |  |
|  | *Cephalopholis sonnerati* |  |  | + |  |  |  |  |
|  | *Cephalopholis spiloparaea* |  |  | + |  |  |  |  |
|  | *Cephalopholis urodeta* |  |  | + |  |  |  |  |
|  | *Chromileptes altivelis* |  |  | + |  |  |  |  |
|  | *Epinephelus coeruleopunctatus* |  |  | + |  |  |  |  |
|  | *Epinephelus cyanopodus* |  |  | + |  |  |  |  |
|  | *Epinephelus fasciatus* |  |  | + |  |  |  |  |
|  | *Epinephelus hexagonatus* |  |  | + |  |  |  |  |
|  | *Epinephelus maculatus* |  |  | + |  |  |  |  |
|  | *Epinephelus malabaricus* |  |  | + |  |  |  |  |
|  | *Epinephelus merra* |  |  | + |  |  |  |  |
|  | *Epinephelus quoyanus* |  |  | + |  |  |  |  |
|  | *Plectropomus leopardus* |  |  | + |  |  |  |  |
|  | *Variola louti* |  |  | + |  |  |  |  |
| Grammistidae | *Grammistes sexlineatus* |  |  | + |  |  | + |  |
| Bramidae | ***Brama dussumieri*** |  |  |  |  |  | + | 59I, 61B |
|  | *Brama japonica* |  |  |  |  |  | + |  |
|  | *Brama myersi* |  | + |  |  |  |  |  |
|  | ***Brama orcini*** |  |  |  |  |  |  | 59H, 61C |
| Monodactylidae | *Monodactylus argenteus* |  |  | + |  |  |  |  |
| Priacanthidae | *Heteropriacanthus cruentatus* |  |  | + |  |  | + |  |
|  | *Priacanthus hamrur* | + | + | + |  |  |  |  |
|  | *Priacanthus macracanthus* |  | + |  |  |  |  |  |
|  | *Priacanthus zaiserae* |  |  |  |  |  |  | 59E, 61D |
| Chaetodontidae | *Chaetodon auriga* | + | + | + |  |  |  |  |
|  | *Chaetodon auripes* | + | + | + |  |  | + |  |
|  | *Chaetodon baronessa* |  | + | + |  |  |  |  |
|  | *Chaetodon citrinellus* |  |  | + |  |  |  |  |
|  | *Chaetodon ephippium* |  | + | + |  |  |  |  |
|  | *Chaetodon guentheri* |  |  | + |  |  |  |  |
|  | *Chaetodon kleinii* |  | + | + |  |  |  |  |
|  | *Chaetodon lineolatus* |  | + | + |  |  |  |  |
|  | *Chaetodon lunula* |  | + | + | + |  |  |  |
|  | *Chaetodon lunulatus* |  |  | + |  |  |  |  |
|  | *Chaetodon melannotus* |  | + | + |  |  |  |  |
|  | *Chaetodon ornatissimus* |  | + | + |  |  |  |  |
|  | *Chaetodon plebeius* |  | + | + |  |  |  |  |
|  | *Chaetodon punctatofasciatus* |  | + | + |  |  |  |  |
|  | *Chaetodon rafflesii* |  | + | + |  |  |  |  |
|  | *Chaetodon speculum* |  | + | + |  |  |  |  |
|  | *Chaetodon trifascialis* |  | + | + |  |  |  |  |
|  | *Chaetodon trifasciatus* | + |  |  |  |  |  |  |
|  | *Chaetodon ulietensis* |  | + | + |  |  |  |  |
|  | *Chaetodon unimaculatus* |  | + |  |  |  |  |  |
|  | *Chaetodon vagabundus* |  | + | + | + |  |  |  |
|  | *Chaetodon wiebeli* | + | + | + |  |  |  |  |
|  | *Chaetodon xanthurus* | + | + | + |  |  |  |  |
|  | *Forcipiger flavissimus* |  |  | + |  |  |  |  |
|  | *Hemitaurichthys polylepis* |  |  | + |  |  |  |  |
|  | *Heniochus acuminatus* | + | + | + |  |  |  |  |
|  | *Heniochus chrysostomus* | + | + | + |  |  |  |  |
|  | *Heniochus monoceros* | + | + | + |  |  |  |  |
|  | *Heniochus singularius* | + | + | + |  |  |  |  |
|  | *Heniochus varius* | + | + | + |  |  |  |  |
| Pomacanthidae | *Apolemichthys trimaculatus* |  |  | + |  |  |  |  |
|  | *Centropyge bicolor* |  |  | + |  |  |  |  |
|  | *Centropyge bispinosa* | + | + | + |  |  |  |  |
|  | *Centropyge ferrugata* |  |  | + |  |  |  |  |
|  | *Centropyge fisheri* | + | + | + |  |  |  |  |
|  | *Centropyge heraldi* |  |  | + |  |  |  |  |
|  | *Centropyge tibicen* | + | + | + |  |  |  |  |
|  | *Centropyge vrolikii* | + | + | + |  |  |  |  |
|  | *Genicanthus watanabei* |  |  | + |  |  |  |  |
|  | *Pomacanthus imperator* | + | + | + |  |  |  |  |
|  | *Pomacanthus semicirculatus* | + | + | + |  |  |  |  |
|  | *Pomacanthus sexstriatus* | + | + | + |  |  |  |  |
|  | *Pygoplites diacanthus* | + | + | + |  |  |  |  |
| Malacanthidae | *Malacanthus brevirostris* | + | + | + |  |  |  |  |
|  | *Malacanthus latovittatus* | + | + | + |  |  |  |  |
| Haemulidae | *Diagramma pictum* |  |  | + |  |  |  |  |
|  | *Plectorhinchus chaetodonoides* | + | + | + |  |  |  |  |
|  | *Plectorhinchus cinctus* | + | + | + |  |  |  |  |
|  | *Plectorhinchus diagrammus* | + | + |  |  |  |  |  |
|  | *Plectorhinchus gibbosus* | + | + |  |  |  |  |  |
|  | *Plectorhinchus lessonii* | + | + | + |  |  |  |  |
|  | *Plectorhinchus lineatus* | + | + | + |  |  |  |  |
|  | *Plectorhinchus pica* | + | + | + |  |  |  |  |
|  | *Plectorhinchus vittatus* |  |  | + |  |  |  |  |
| Lutjanidae | *Aphareus rutilans* | + | + | + |  |  |  |  |
|  | *Aprion virescens* | + | + | + |  |  |  |  |
|  | *Caesio caerulaurea* |  |  | + |  |  |  |  |
|  | *Caesio teres* | + | + |  |  |  |  |  |
|  | *Lutjanus argentimaculatus* |  |  | + | + |  |  |  |
|  | *Lutjanus bohar* |  |  | + |  |  |  |  |
|  | *Lutjanus boutton* |  |  | + |  |  |  |  |
|  | *Lutjanus decussatus* |  |  | + |  |  |  |  |
|  | *Lutjanus fulviflamma* | + | + | + | + |  |  |  |
|  | *Lutjanus fulvus* | + | + | + |  |  |  |  |
|  | *Lutjanus gibbus* | + | + | + |  |  |  |  |
|  | *Lutjanus johnii* | + | + | + |  |  |  |  |
|  | *Lutjanus kasmira* | + | + | + |  |  |  |  |
|  | *Lutjanus monostigma* | + | + | + |  |  |  |  |
|  | *Lutjanus rivulatus* | + | + | + |  |  |  |  |
|  | *Lutjanus russellii* |  | + | + |  |  |  |  |
|  | *Lutjanus vitta* |  | + | + |  |  |  |  |
|  | *Macolor niger* |  | + | + |  |  |  |  |
|  | *Paracaesio xanthurus* |  |  | + |  |  |  |  |
|  | *Pterocaesio digramma* | + | + | + |  |  |  |  |
|  | *Pterocaesio marri* |  |  | + |  |  |  |  |
|  | *Pterocaesio tile* | + | + | + |  |  |  |  |
| Cirrhitidae | *Cirrhitichthys aprinus* |  |  | + |  |  |  |  |
|  | *Cirrhitichthys falco* |  |  | + |  |  |  |  |
|  | *Cirrhitichthys oxycephalus* |  |  | + |  |  |  |  |
|  | *Cirrhitus pinnulatus* |  |  | + |  |  |  |  |
|  | *Cyprinocirrhites polyactis* |  |  | + |  |  |  |  |
|  | *Paracirrhites arcatus* | + | + |  |  |  |  |  |
|  | *Paracirrhites forsteri* | + | + |  |  |  |  |  |
| Cepolidae | ***Owstonia aurora*** |  |  |  |  |  |  | 59G, 61G |
|  | ***Owstonia grammodon*** |  |  |  |  |  |  | 59F, 61F |
| Bempropidae | *Bembrops caudimacula* |  |  |  |  |  |  | 60A, 61E |
| Siganidae | *Siganus argenteus* | + | + | + |  |  |  |  |
|  | *Siganus fuscescens* | + |  | + |  |  |  |  |
|  | *Siganus guttatus* |  |  | + |  |  |  |  |
|  | *Siganus puellus* | + | + | + |  |  |  |  |
|  | *Siganus punctatissimus* | + | + | + |  |  |  |  |
|  | *Siganus punctatus* | + | + |  |  |  |  |  |
|  | *Siganus spinus* |  |  | + |  |  |  |  |
|  | *Siganus virgatus* | + | + | + |  |  |  |  |
| Scorpaenidae | *Caracanthus maculatus* | + | + | + |  |  |  |  |
|  | *Dendrochirus biocellatus* |  |  | + |  |  |  |  |
|  | *Dendrochirus zebra* |  |  | + |  |  |  |  |
|  | *Ectreposebastes imus* |  |  |  |  |  |  | 60C, 62E |
|  | *Lioscorpius longiceps* |  |  |  |  |  |  | 60B, 62B |
|  | ***Lythrichthys cypho*** |  |  |  |  |  |  | 60H, 62F |
|  | ***Lythrichthys eulabes*** |  |  |  |  |  |  | 60I, 62C |
|  | *Parascorpaena mcadamsi* | + | + | + |  |  |  |  |
|  | *Parascorpaena mossambica* | + | + | + |  |  |  |  |
|  | *Phenacoscorpius megalops* |  |  |  |  |  |  | 60E, 62D |
|  | *Pterois antennata* | + | + | + |  |  |  |  |
|  | *Pterois volitans* | + | + | + |  |  |  |  |
|  | *Scorpaena neglecta* |  |  | + |  |  |  |  |
|  | *Scorpaenodes kelloggi* | + | + | + |  |  |  |  |
|  | *Scorpaenodes varipinnis* |  |  | + |  |  |  |  |
|  | *Scorpaenopsis cirrosa* | + | + | + |  |  |  |  |
|  | *Scorpaenopsis diabolus* | + | + | + |  |  |  |  |
|  | *Sebastapistes cyanostigma* | + | + | + |  |  |  |  |
|  | *Sebastapistes strongia* |  | + | + |  |  |  |  |
|  | *Lythrichthys longimanus* |  | + |  |  |  |  |  |
|  | *Synanceia verrucosa* | + | + | + |  |  |  |  |
|  | ***Setarches guentheri*** |  |  |  |  |  |  | 60F, 62A |
| Plectrogeniidae | *Plectrogenium kamoharai* | + | + |  |  |  |  | 60D, 62G |
| Aploactinidae | *Erisphex pottii* |  |  |  |  |  |  | 60G, 62H |
| Triglidae | *Chelidonichthys spinosus* |  |  |  |  |  |  | 63O, 64I |
|  | ***Lepidotrigla pectoralis*** |  |  |  |  |  |  | 63F, 64F |
|  | ***Lepidotrigla* sp.** |  |  |  |  |  |  | 63E, 64E |
|  | *Lepidotrigla oglina* |  | + |  |  |  |  |  |
|  | ***Pterygotrigla cajorarori*** |  |  |  |  |  |  | 63B, 64H |
|  | *Pterygotrigla macrorhynchus* |  |  |  |  |  |  | 63A, 64G |
| Peristediidae | *Heminodus philippinus* |  |  |  |  |  |  | 63D |
|  | ***Paraheminodus murrayi*** |  |  |  |  |  |  | 63C, 64D |
|  | *Peristedion orientale* |  | + |  |  |  |  | 63H, 64K |
|  | *Peristedion riversandersoni* |  |  |  |  |  |  | 63G, 64J |
|  | ***Satyrichthys milleri*** |  |  |  |  |  |  | 63N, 64C |
|  | *Scalicus hians* |  |  |  |  |  |  | 63I, 64B |
|  | *Scalicus orientalis* |  |  |  |  |  |  | 63M, 64A |
|  | *Satyrichthys piercei* |  | + |  |  |  |  |  |
| Platycephalidae | *Cociella crocodilus* |  | + |  |  |  |  |  |
|  | *Cirrhitus pinnulatus* | + | + |  |  |  |  |  |
| Hoplichthyidae | *Hoplichthys fasciatus* |  |  |  |  |  |  | 63K, 66B |
|  | ***Hoplichthys filamentosus*** |  |  |  |  |  |  | 63L, 66C |
|  | *Hoplichthys gilberti* |  |  |  |  |  |  | 63J, 66A |
| Psychrolutidae | ***Stlengis distoechus*** |  |  |  |  |  |  | 65B, 66E |
|  | ***Psychrolutes macrocephalus*** |  |  |  |  |  |  | 65C, 66D |
| Ephippidae | *Platax boersii* |  |  | + |  |  |  |  |
|  | *Platax teira* |  |  | + |  |  |  |  |
|  | *Platax orbicularis* |  |  | + |  |  |  |  |
| Emmelichthyidae | *Erythrocles schlegelii* |  |  |  |  |  |  | 65A, 68A |
| Zanclidae | *Zanclus cornutus* | + | + | + | + |  | + |  |
| Acanthuridae | *Acanthurus bariene* | + | + | + |  |  |  |  |
|  | *Acanthurus dussumieri* | + | + | + |  |  |  |  |
|  | *Acanthurus japonicus* | + | + | + |  |  |  |  |
|  | *Acanthurus leucosternon* |  |  |  |  |  | + |  |
|  | *Acanthurus lineatus* | + | + | + |  |  |  |  |
|  | *Acanthurus maculiceps* | + | + | + |  |  |  |  |
|  | *Acanthurus mata* |  |  | + |  |  |  |  |
|  | *Acanthurus nigricans* |  |  | + |  |  |  |  |
|  | *Acanthurus nigrofuscus* | + | + | + |  |  |  |  |
|  | *Acanthurus olivaceus* | + | + | + |  |  |  |  |
|  | *Acanthurus pyroferus* |  |  | + |  |  |  |  |
|  | *Acanthurus thompsoni* | + | + | + |  |  |  |  |
|  | *Acanthurus triostegus* | + | + | + |  |  |  |  |
|  | *Acanthurus xanthopterus* | + | + | + |  |  |  |  |
|  | *Ctenochaetus binotatus* | + | + | + |  |  |  |  |
|  | *Ctenochaetus striatus* | + | + | + |  |  |  |  |
|  | *Naso annulatus* |  |  | + |  |  |  |  |
|  | *Naso brevirostris* | + | + | + |  |  |  |  |
|  | *Naso hexacanthus* |  |  | + |  |  |  |  |
|  | *Naso lituratus* | + | + | + |  |  |  |  |
|  | *Naso thynnoides* |  |  | + |  |  |  |  |
|  | *Naso unicornis* | + | + | + |  |  |  |  |
|  | *Naso vlamingii* |  |  | + |  |  |  |  |
|  | *Paracanthurus hepatus* |  |  | + |  |  |  |  |
|  | *Zebrasoma flavescens* |  |  | + |  |  |  |  |
|  | *Zebrasoma scopas* | + | + | + |  |  |  |  |
|  | *Zebrasoma veliferum* | + | + | + |  |  |  |  |
| Nemipteridae | *Pentapodus caninus* |  | + | + |  |  |  |  |
|  | *Scolopsis bilineata* | + | + | + |  |  |  |  |
|  | *Scolopsis lineata* | + | + | + |  |  |  |  |
|  | *Scolopsis monogramma* | + | + | + |  |  |  |  |
|  | *Scolopsis trilineata* | + | + | + | + |  |  |  |
|  | *Scolopsis xenochrous* |  |  | + |  |  |  |  |
| Lethrinidae | *Gnathodentex aureolineatus* |  | + | + |  |  |  |  |
|  | *Gymnocranius griseus* |  |  | + |  |  |  |  |
|  | *Lethrinus atkinsoni* |  |  | + |  |  |  |  |
|  | *Lethrinus erythracanthus* | + | + | + |  |  |  |  |
|  | *Lethrinus harak* | + | + | + | + |  |  |  |
|  | *Lethrinus lentjan* |  |  | + |  |  |  |  |
|  | *Lethrinus nebulosus* |  |  | + | + |  |  |  |
|  | *Lethrinus obsoletus* |  |  | + |  |  |  |  |
|  | *Lethrinus ornatus* | + | + | + |  |  |  |  |
|  | *Lethrinus reticulatus* | + | + | + |  |  |  |  |
|  | *Lethrinus rubrioperculatus* |  |  | + |  |  |  |  |
|  | *Lethrinus variegatus* | + | + | + |  |  |  |  |
|  | *Monotaxis grandoculis* | + |  | + |  |  |  |  |
|  | *Wattsia mossambica* |  | + |  |  |  |  |  |
| Lophiidae | ***Lophiodes iwamotoi*** |  |  |  |  |  |  | 67A, 68B |
|  | ***Lophiodes lugubris*** |  |  |  |  |  |  | 67B, 68D |
|  | *Lophiodes mutilus* |  |  |  |  |  |  | 67D, 68C |
|  | *Lophiodes naresi* |  |  |  |  |  |  | 67C, 68E |
|  | ***Lophiodes triradiatus*** |  |  |  |  |  |  | 67E, 68F |
| Antennariidae | *Antennarius pictus* |  | + | + |  |  |  |  |
| Chaunacidae | ***Chaunax apus*** |  |  |  |  |  |  | 69D, 71B |
|  | ***Chaunax breviradius*** |  |  |  |  |  |  | 69C, 71C |
|  | *Chaunax penicillatus* |  |  |  |  |  |  | 69A, 71A |
|  | ***Chaunax* sp.** |  |  |  |  |  |  | 69B, 71D |
| Ogcocephalidae | *Coelophrys micropus* |  |  |  |  |  |  | 70L, 71E |
|  | *Halicmetus reticulatus* |  | + |  |  |  |  | 70K, 71O |
|  | *Halicmetus ruber* |  |  |  |  |  |  | 70I, 71M |
|  | ***Halicmetus* cf. *ruber*** |  |  |  |  |  |  | 70J, 71N |
|  | *Halieutaea coccinea* |  |  |  |  |  |  | 70C, 71P |
|  | ***Halieutopsis echinoderma*** |  |  |  |  |  |  | 70A, 71K |
|  | ***Halieutopsis nasuta*** |  |  |  |  |  |  | 70B, 71L |
|  | ***Halieutopsis* sp.** |  |  |  |  |  |  | 70D, 71J |
|  | *Halieutopsis simula* |  | + |  |  |  |  |  |
|  | *Malthopsis annulifera* |  |  |  |  |  |  | 70E, 71I |
|  | ***Malthopsis kobayashii*** |  |  |  |  |  |  | 70G, 71H |
|  | *Malthopsis mitrigera* |  |  |  |  |  |  | 70F, 71F |
|  | *Malthopsis tiarella* |  |  |  |  |  |  | 70H, 71G |
| Ceratiidae | *Ceratias holboelli* |  | + |  |  |  |  |  |
| Triacanthodidae | *Bathyphylax bombifrons* |  | + |  |  |  | + | 72D, 73B |
|  | *Halimochirurgus alcocki* |  | + |  |  |  |  | 72E, 73D |
|  | *Halimochirurgus centriscoides* |  | + |  |  |  |  |  |
|  | *Macrorhamphosodes uradoi* |  | + |  |  |  |  |  |
|  | *Paratriacanthodes retrospinis* |  | + |  |  |  |  | 72C, 73C |
|  | *Triacanthodes anomalus* |  |  |  |  |  |  | 72B, 73A |
|  | *Tydemania navigatoris* |  | + |  |  |  |  | 72A, 73E |
| Ostraciidae | *Ostracion cubicum* | + | + | + |  |  |  |  |
|  | *Ostracion meleagris* |  |  | + |  |  |  |  |
| Balistidae | *Abalistes stellatus* |  |  | + |  |  |  |  |
|  | *Balistapus undulatus* | + | + | + |  |  |  |  |
|  | *Balistoides conspicillum* | + | + | + |  |  |  |  |
|  | *Balistoides viridescens* | + | + | + |  |  |  |  |
|  | *Canthidermis macrolepis* |  |  |  |  |  | + |  |
|  | *Melichthys vidua* | + |  | + |  |  |  |  |
|  | *Odonus niger* | + | + | + |  |  |  |  |
|  | *Pseudobalistes flavimarginatus* |  |  | + |  |  |  |  |
|  | *Pseudobalistes fuscus* |  |  | + |  |  |  |  |
|  | *Rhinecanthus aculeatus* | + | + | + |  |  |  |  |
|  | *Rhinecanthus rectangulus* | + | + | + |  |  |  |  |
|  | *Sufflamen bursa* | + | + | + |  |  |  |  |
|  | *Sufflamen chrysopterum* | + | + | + |  |  |  |  |
|  | *Sufflamen fraenatum* | + | + | + |  |  |  |  |
|  | *Thamnaconus modestus* | + |  |  |  |  |  |  |
|  | *Xanthichthys auromarginatus* |  |  | + |  |  | + |  |
| Monacanthidae | *Acreichthys tomentosus* |  |  | + |  |  |  |  |
|  | *Cantherhines dumerilii* |  |  | + |  |  |  |  |
|  | *Cantherhines fronticinctus* |  |  | + |  |  |  |  |
|  | *Cantherhines pardalis* |  |  | + |  |  |  |  |
|  | *Paraluteres prionurus* |  |  | + |  |  |  |  |
|  | *Pervagor janthinosoma* |  |  | + |  |  |  |  |
|  | *Pervagor melanocephalus* |  |  | + |  |  |  |  |
|  | *Rudarius excelsus* |  |  | + |  |  |  |  |
|  | *Thamnaconus modestus* |  |  | + |  |  |  |  |
| Tetraodontidae | *Arothron hispidus* | + | + | + | + |  | + |  |
|  | *Arothron manilensis* | + | + | + |  |  |  |  |
|  | *Arothron mappa* |  |  | + |  |  |  |  |
|  | *Arothron nigropunctatus* | + | + | + |  |  |  |  |
|  | *Arothron stellatus* |  |  | + |  |  |  |  |
|  | *Canthigaster axiologus* |  |  | + |  |  |  |  |
|  | *Canthigaster bennetti* |  |  | + |  |  |  |  |
|  | *Canthigaster epilampra* |  |  | + |  |  |  |  |
|  | *Canthigaster janthinoptera* | + | + | + |  |  |  |  |
|  | *Canthigaster valentini* | + | + | + |  |  |  |  |
|  | *Lagocephalus lagocephalus* |  |  |  |  |  | + |  |
|  | *Sphoeroides pachygaster* | + | + |  |  |  |  |  |
|  | *Takifugu poecilonotus* | + | + |  |  |  |  |  |
| Diodontidae | *Diodon hystrix* | + | + | + |  |  |  |  |
|  | *Diodon liturosus* | + | + | + |  |  |  |  |
